# Network Meta-analysis on Disconnected Evidence Networks When Only Aggregate Data Are Available: Modified Methods to Include Disconnected Trials and Single-Arm Studies while Minimizing Bias

**DOI:** 10.1177/0272989X221097081

**Published:** 2022-05-07

**Authors:** Howard Thom, Joy Leahy, Jeroen P. Jansen

**Affiliations:** Bristol Medical School, University of Bristol, UK (HT); Clifton Insight, Bristol, UK; National Centre for Pharmacoeconomic, Dublin, Ireland; School of Pharmacy, University of California, San Francisco, USA

**Keywords:** disconnected evidence networks, evidence synthesis, indirect treatment comparison, network meta-analysis

## Abstract

**Background:**

Network meta-analysis (NMA) requires a connected network of randomized controlled trials (RCTs) and cannot include single-arm studies. Regulators or academics often have only aggregate data. Two aggregate data methods for analyzing disconnected networks are random effects on baseline and aggregate-level matching (ALM). ALM has been used only for single-arm studies, and both methods may bias effect estimates.

**Methods:**

We modified random effects on baseline to separate RCTs connected to and disconnected from the reference and any single-arm studies, minimizing the introduction of bias. We term our modified method *reference prediction*. We similarly modified ALM and extended it to include RCTs disconnected from the reference. We tested these methods using constructed data and a simulation study.

**Results:**

In simulations, bias for connected treatments for ALM ranged from −0.0158 to 0.051 and for reference prediction from −0.0107 to 0.083. These were low compared with the true mean effect of 0.5. Coverage ranged from 0.92 to 1.00. In disconnected treatments, bias of ALM ranged from −0.16 to 0.392 and of reference prediction from −0.102 to 0.40, whereas coverage of ALM ranged from 0.30 to 0.82 and of reference prediction from 0.64 to 0.94. Under fixed study effects for disconnected evidence, bias was similar, but coverage was 0.81 to 1.00 for reference prediction and 0.18 to 0.76 for ALM. Trends of similar bias but greater coverage for reference prediction with random study effects were repeated in constructed data.

**Conclusions:**

Both methods with random study effects seem to minimize bias in treatment connected to the reference. They can estimate treatment effects for disconnected treatments but may be biased. Reference prediction has greater coverage and may be recommended overall.

**Highlights:**

- Two methods were modified for network meta-analysis on disconnected networks and for including single-arm observational or interventional studies in network meta-analysis using only aggregate data and for minimizing the bias of effect estimates for treatments only in trials connected to the reference.
- Reference prediction was developed as a modification of random effects on baseline that keeps analyses of trials connected to the reference separately from those disconnected from the reference and from single-arm studies. The method was further modified to account for correlation in trials with more than 2 arms and, under random study effects, to estimate variance in heterogeneity separately in connected and disconnected evidence.
- Aggregate-level matching was extended to include trials disconnected from the reference, rather than only single-arm studies. The method was further modified to separately estimate treatment effects and heterogeneity variance in the connected and disconnected evidence and to account for the correlation between arms in trials with more than 2 arms.
- Performance was assessed using a constructed data example and simulation study.
- The methods were found to have similar, and sometimes low, bias when estimating the relative effects for disconnected treatments, but reference prediction with random study effects had the greatest coverage.
- The use of reference prediction with random study effects for disconnected networks is recommended if no individual patient data or alternative real-world evidence is available.

## Introduction

**Figure 1:**
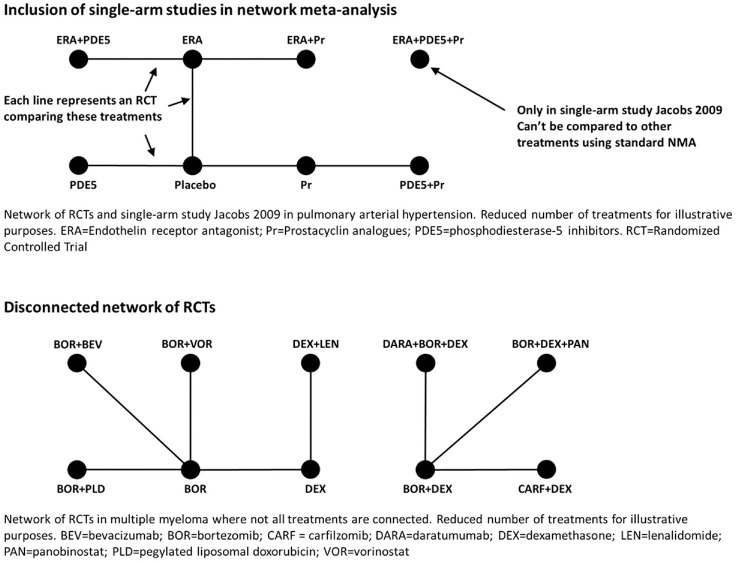
Example of single-arm studies in a network meta-analysis in pulmonary arterial hypertension from Thom in 2015^4^ and of a disconnected network of randomized controlled trials in multiple myeloma from Schmitz in 2018.^6^

Network meta-analysis (NMA) is a method endorsed by the UK National Institute for Health and Care Excellence (NICE) and international scientific societies for estimating relative treatment effects between interventions based on randomized controlled trials (RCTs) in which each study compares a subset of the interventions of interest.^1,2^ NMA requires treatments to be connected by a network of RCTs to benefit from randomization and minimize the risk of biased relative to treatment effect estimates.^3^ Disconnected networks are common in rare diseases, for which RCT recruitment is challenging.^4–6^ Disconnected networks can also arise if analyzing rarely reported outcomes or splitting treatments by doses or time points. Furthermore, traditional NMA is restricted to RCTs and cannot incorporate single-arm observational or interventional studies, which are again common in rare disease or in cases in which a control arm would be unethical or there has been a breakthrough in treatment efficacy and RCTs are not judged necessary.^7–9^ Single-arm studies and disconnected RCT networks are illustrated with examples from the literature in Figure 1.

There are many methods available to overcome disconnected RCT networks or to incorporate single-arm studies, and these have been described in a recent thorough review.^5^ Real-world evidence, such as registry or insurance claims data, can be used to fill RCT evidence gaps and connect networks or to replace the RCT network entirely, as has been done in hip replacement surgery.^10,11^ Real-world evidence is not always available, is likely focused on only established treatments, and may be subject to implicit bias.^12^ If individual patient data are available from all RCTs and single-arm studies, propensity matching or regression adjustment techniques could be used to conduct comparisons.^13^ Even if individual patient data are available from only 1 RCT or 1 single-arm study, matching-adjusted indirect comparison, simulated treatment comparison, and multilevel network meta-regression could be used to conduct unanchored indirect comparisons on disconnected networks.^14,15^ However, individual patient data from commercial RCTs or single-arm studies are frequently not available to academics or to authors of national clinical guidelines. Furthermore, it should be stated that these methods are not as reliable as connected networks of RCTs are.^15^

If only aggregate data are available, methods are still available to connect networks. Model-based NMA models dose-response relationships and can be used to connect disconnected networks if studies on multiple doses are available and if the dose-response relationship can be correctly specified.^16,17^ A class effect NMA can be used to connect networks if disconnected treatments have a similar mechanism of action or molecular structure to a connected treatment.^5^ If interventions in a disconnected network are composed of common components, component NMA has been proposed to reconnect networks.^18,19^ Modeling the relation between treatment effects on different scales, multiple outcomes NMA can leverage connected networks on 1 outcome to connect disconnected networks, or strengthen sparse networks, on another outcome.^20^ All of these rely on special circumstances and strong assumptions.

Random effects on baseline can be used to connect a disconnected network when only aggregate data are available.^4^ Random effects on baseline assumes the response to control treatments are exchangeable across studies and thus removes the need for a connected network with the price of interfering with randomization in RCTs already connected to the intervention of interest.^21–23^ Another method termed *aggregate-level matching* (ALM) can include single-arm studies by matching them with an arm of a selected RCT that is connected to the intervention of interest, creating a new RCT. However, this method has not yet been extended to RCTs disconnected from the intervention of interest.^24^

We first introduce the standard method of contrast-based NMA with independent baselines in the next section (“Contrast-Based Network Meta-analysis Model for a Connected Network with Independent Baseline”) before giving more detail on the issues with random effects in subsequent section (“Network Meta-analysis Model with Random Effects on Baseline and Its Problems”). In the “Modified Evidence Synthesis Models for Disconnected Networks with Aggregate-Level Data” section, we present our modified methods for the analysis of disconnected networks using only aggregate data and prevent bias in the RCTs already connected to the target comparator. We test these on an artificially disconnected network based on a real literature review in atrial fibrillation and in a simulation study in the sections “Assessment by Constructed Data” and “Assessment by Simulation Study,” respectively.^25,26^ We close by drawing conclusions and making methodology recommendations.

## Existing Evidence Synthesis Models

### Contrast-Based Network Meta-analysis Model for a Connected Network with Independent Baseline

We consider only NMA on binomial data with a logistic link function, but the methods we present can be applied to any generalized linear model for any type of data.

If each arm k of trial i reports $r_{ik}$ events out of $n_{ik}$ patients, and patients on this arm receive treatment $t_{ik}$ , the likelihood is binomial

$$r_{ik}\sim Binomial\left(p_{ik},n_{ik}\right)$$

The probability of event $p_{ik}$ is modeled via

$$logit\left(p_{ik}\right)=\mu_{i}+\delta_{ik}$$

The log odds of an event on the baseline treatment $\delta_{i1}$ in trial i is modeled as $\mu_{i}$ . If using random study effects, the treatment effect $\delta_{ik}$ is modeled as

$$\delta_{ik}\sim Normal\left(d_{t_{i1}t_{ik}},\sigma^{2}\right)$$

The $\sigma^{2}$ is some estimated heterogeneity variance $\sigma^{2}$ representing variation in treatment effects across trials. If using fixed effects, the relation is

$$\delta_{ik}=d_{t_{i1}t_{ik}}$$

The $d_{t_{\mathrm{ib}}t_{ik}}$ are related to the basic parameters

$$d_{t_{i1}t_{ik}}=d_{1t_{ik}}-d_{1t_{i1}}$$

The treatment denoted 1 is the so-called “reference” treatment, which is common across the network of evidence and relative to which the basic log odds ratios $d_{1x}$ for all treatments x are defined. This reference treatment is distinct from the baseline treatment $t_{i1}$ in study 1.

The baseline effects $\mu_{i}$ are nuisance parameters that must be canceled out and on which we assume a vague prior

$$\mu_{i}\sim N\left(0,10^{2}\right)$$

They are thus assumed to be independent of each other, so information on the relative treatment effects $d_{t_{i1}t_{ik}}$ is coming only within and not across trials.

This independent baseline model preserves randomization within trials. Comparison of any 2 treatments x and y is through the consistency equation

$$d_{xy}=d_{1y}-d_{1x}$$

However, this requires a connected network of RCT evidence between treatments *x* and y . Also, as treatment effects are estimated only relative to an RCT-specific baseline, single-arm evidence cannot be used.

Under random effects in which $\delta_{ik}\sim Normal\left(d_{t_{i1}t_{ik}},\sigma^{2}\right),$ trials with more than 2 arms have more than 1 $\delta_{ik}$ , and they are correlated as they are both relative to the same baseline arm in trial i . The correction for this is described in the Appendix.

### Network Meta-analysis Model with Random Effects on Baseline and Its Problems

The model described by Thom et al.^4^ and Beliveau et al.^22^ and generally called the “random effects on baseline” puts a random effect model on reference treatment 1 :

$$\mu_{1,i}\sim N\left(m_{1},\sigma_{\mu }\right)$$

This models the log odds of the event on the reference treatment, even in trials that do not include the reference treatment. For any arm of a connected RCT, disconnected RCT, or single-arm study, the probability is modeled as

$$logit\left(p_{ik}\right)=\mu_{1,i}+\delta_{ik}$$

The treatment effects $\delta_{ik}$ for treatment $t_{ik}$ are relative to overall reference treatment 1 rather than to trial-specific baseline treatment $\delta_{ik}t_{i1}$ . Depending on the fixed or random study effects, we therefore use either

$$\delta_{ik}=d_{1t_{ik}}$$

or

$$\delta_{ik}\sim Normal\left(d_{1t_{ik}},\sigma^{2}\right)$$

This thus allows the inclusion of RCTs disconnected from the reference or single-arm studies. Unlike the independent baseline model, this approach assumes exchangeability of reference log odds $\mu_{1,i}$ across trials. Arm-based NMA is similar but models all arms independently and thus breaks randomization, but we do consider this method further.

**Figure 2:**
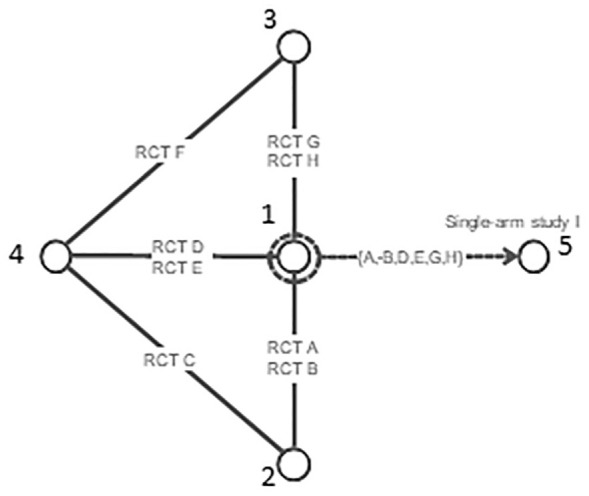
Using random effects on baseline to predict response on reference treatment A using studies that included A. However, the random effects on baseline model interferes with randomization in randomized controlled trials on treatments 2, 3, and 4.

The downside to using random effects on baseline is that treatment effects $\delta_{ik}$ in RCTs already connected to the reference are biased by the across-study information on $\mu_{1,i}$ .^27,28^ For example, a common finding across clinical areas is that response on placebo, often the reference treatment in NMA, is improving over time, commonly called “placebo creep.”^21^ This would suggest greater $\mu_{1,i}$ the later RCT i was conducted. Allowing later RCTs to influence the placebo effect in earlier RCTs would pull placebo effects up in these earlier RCTs, thus biasing against treatments studied in such RCTs and vice versa for the impact on later treatments in later RCTs. This would bias treatment effects on treatments 2, 3, and 4 in Figure 2. This bias has been discussed at length in the literature in the context of arm-based NMA, which models all RCT arms independently and breaks randomization.^28,29^ Recent work has shown this bias to be limited, but its potential to cause problems remains, as not every scenario has been explored in simulation or practice.^22^

Typically, for random effects models, the same heterogeneity variance $\sigma^{2}$ is used for the RCTs connected and disconnected from the reference, but this could lead to influence of the latter on $\sigma^{2}$ and thus interfere with randomization in the RCTs connected to the reference.

## Modified Evidence Synthesis Models for Disconnected Networks with Aggregate-Level Data

We propose modifications and extensions of random effects on baseline and ALM to minimize bias when analyzing disconnected networks using only aggregate data.

### Reference Prediction

Our method of reference prediction predicts outcomes on the reference treatment in RCTs disconnected from the reference and single-arm studies. This involves two modifications of random effects on baseline. The first is to analyze RCTs connected to the reference separately from those disconnected from the reference and from single-arm studies. We also modify the model under random study effects to correctly account for RCTs with more than 2 arms and to estimate the heterogeneity variance separately in RCTs connected to the reference and those disconnected from the reference. In the section titled “Reference Prediction with Covariates,” we further modify the method to include covariate effects and improve the prediction of the reference response.

To avoid interfering with randomization in RCTs connected to the reference, we propose splitting them into $i=1,\ldots ,n_{s}$ RCTs that are connected to the reference and $i\prime =1,\ldots ,n\prime_{s}$ RCT that are disconnected from the reference. The total number of RCTs is $n_{s}+n\prime_{s}$ , and the method is identifiable so long as $n_{s}\geq 2$ and $n\prime_{s}\geq 1$ . We furthermore label $i\prime \prime =1,\ldots ,n_{s}^{\prime \prime }$ with $n_{s}^{\prime \prime }\geq 1$ as single-arm (observational or interventional) studies. We model the trials $i=1,\ldots ,n_{s}$ using standard contrast-based NMA with independent baselines

$$logit\left(p_{ik}\right)=\mu_{i}+\delta_{ik}$$

$$\mu_{i}\sim N\left(0,10^{2}\right)$$

However, we make a second use of the $n_{s}^{ref}$ , where $n_{s}^{ref}\leq n_{s}$ , RCTs that include the reference treatment as one of their arms. We use these RCTs $i=1,\ldots ,\;n_{s}^{ref}$ to build a separate random effects meta-analysis model, with identifiability requirement $n_{s}^{ref}\geq 2$ , of outcomes on the reference treatment arms

$$r_{i1}^{ref}\sim Binomial\left(p_{i1}^{ref},n_{i1}^{ref}\right)$$

$$logit\left(p_{i1}^{ref}\right)=\mu_{i}^{ref}$$

$$\mu_{i}^{ref}\sim N\left(m_{1},\sigma_{\mu }\right)$$

We importantly note that there is no link in the model between $\mu_{i}^{ref}$ and either $\mu_{i}$ or any of the arm-specific treatment effects $\delta_{i,\mathrm{bk}}$ , although the $n_{i1}^{ref}=n_{ik}$ and $r_{i1}^{ref}=r_{ik}$ if $t_{ik}=1$ ; the connected network is thus not biased by this meta-analysis. In the OpenBUGS implementation provided in the Appendix, we achieve this by duplicating the data. This is the same approach as is often taken to generate absolute (e.g., probability) outcomes for cost-effectiveness models.^30^

This meta-analysis model is then used to generate predictions of the log odds of response on reference treatment 1 in RCTs disconnected from the reference i′

$$\mu_{1,i\prime }\sim N\left(m_{1},\sigma_{\mu }\right)$$

$$logit\left(p_{i\prime ,k}\right)=\mu_{1,i\prime }+\delta_{i\prime k}\;for\;allt_{i\prime k}$$

The meta-analysis is therefore called a “reference prediction” model. As in random effects on baseline, the $\delta_{i\prime k}$ are relative to overall reference treatment 1 rather than trial-specific baseline $t_{i\prime 1}$ . Similarly, in single-arm studies i″ where $\delta_{i\prime \prime }$ is relative to reference treatment 1 , we use

$$\mu_{1,i\prime \prime }\sim N\left(m_{1},\sigma_{\mu }\right)$$

$$logit\left(p_{i\prime \prime }\right)=\mu_{1,i\prime \prime }+\delta_{i\prime \prime }$$

As in the independent baseline model, fixed or random effects can be used for the treatment effect $\delta_{i\prime ,k}$ or $\delta_{i\prime \prime ,k}$ . However, under random effects, it is desirable to avoid the influence of the RCTs disconnected from the reference or single-arm studies on the heterogeneity variance $\sigma^{2}$ of the RCTs connected to the reference, as otherwise effects of treatments only in the latter RCTs will have biased estimates. Our second modification to previously published random effects on baseline models is to separate the $\sigma^{2}$ in the RCTs connected to the reference from ${\sigma^{2}}^{\prime }$ in the RCTs disconnected from the reference and ${\sigma^{2}}^{\prime \prime }$ in the single-arm studies. We further use σ as an informative prior on σ′ or σ″ , as there are often too few studies in the network from the reference to adequately estimate a separate heterogeneity variance. This prior is a normal centered on σ , standard deviation equal to that of the Markov Chain Monte Carlo samples of σ from the independent baselines NMA and truncated below at 0.

Finally, the reparameterization of $\delta_{i\prime ,1k}$ in RCTs disconnected from the reference to be relative to the reference treatment, and not the baseline b , requires a modified adjustment for multiarm trials under random study effects. We describe this novel modification in the Appendix.

### Reference Prediction with Covariates

A further modification for reference prediction models is to include covariates. We assume we have some vector of length $n_{c}$ of covariates $x_{\mathrm{ik}}$ for RCTs to the reference, $x_{i\prime k}^{\prime }$ for RCTs disconnected from the reference, and $x_{i}^{\prime \prime }$ for single-arm studies. These can be used in the reference prediction model

$$r_{i1}^{ref}\sim Binomial\left(p_{i1}^{ref},n_{i1}^{ref}\right)$$

$$logit\left(p_{i1}^{ref}\right)=\mu_{i}^{ref}$$

$$\mu_{i}^{ref}\sim N\left(m_{1}+{\beta x}_{i}^{ref},\sigma_{\mu }\right)$$

The β is a vector of $n_{c}$ regression coefficients each with vague priors

$$\beta_{l}\sim N\left(0,10^{2}\right)\mathrm{Where}\;l=1,\ldots ,n_{c}$$

Although the $\beta_{l}$ are fixed, there is still a random effect distribution on the $\mu_{i}^{ref}$ with standard deviation $\sigma_{\mu }$ and centered on $m_{1}+{\beta x}_{i}^{ref}$ . Note the identifiability constraint that $n_{s}^{ref}\geq 2+n_{c}$ so regression on covariates may not always be feasible. The distribution on $\mu_{i}^{ref}$ is then used to improve prediction of the reference response for the RCTs disconnected from the reference

$$\mu_{1,i\prime }\sim N\left(m_{1}+\beta x\prime_{i\prime k},\sigma_{\mu }\right)$$

$$logit\left(p_{i\prime ,k}\right)=\mu_{1,i\prime }+\delta_{i\prime ,k}\;for\;allt_{i\prime k}$$

For single-arm studies, we use

$$\mu_{1,i\prime \prime }\sim N\left(m_{1}+\beta x_{i\prime \prime },\sigma_{\mu }\right)$$

$$logit\left(p_{i\prime \prime }\right)=\mu_{1,i\prime \prime }+\delta_{i\prime \prime }$$

Note that we still assume independent $\delta_{i}$ in the RCTs to the reference with no use of the covariates, preserving randomization. Because of the potential for overfitting and convergence challenges, we recommend models without covariates a priori. Covariate-adjusted models should be used only if model fit statistics, such as Bayesian residual deviance or deviance information criterion (DIC), are substantially lower than models with no covariates.^31,32^ The rule of a ≥5-point difference in DIC being substantial has been recommended in the literature.^33^

### Modified ALM

Our modification to ALM extends the method to include RCTs disconnected from the reference. As for reference prediction, we also modify the method to keep the RCTs connected to the reference separate from those that are disconnected from the reference and from single-arm studies, thus minimizing the introduction of bias. Finally, we modify ALM on random study effects to correctly account for RCTs with more than 2 arms.

ALM, under contrast-based NMA with independent baselines, instead uses a $\mu_{i}^{matched}$ from a selected RCT as a prediction of response on that RCT’s baseline treatment $t_{i1}$ . The selected connected RCT is that with most similar patient and design characteristics to the disconnected RCT i′ or single-arm study i″ . The ALM estimator can also be viewed as a “plug-in” estimator as the $\mu_{i}^{matched}$ is plugged in to the disconnected RCT or single-arm study.

We model RCTs disconnected from the reference as

$$logit\left(p_{i\prime ,k}\right)=\mu_{i}^{matched}+\delta_{i\prime k}\;for\;allt_{i\prime k}$$

This ensures that the same $\mu_{i}^{matched}$ is used in connected RCT i and disconnected RCT i′ . The relative treatment effects are relative to the matched study’s baseline treatment $t_{i1}$ , so $\delta_{i\prime k}\sim Normal(d_{1t_{i\prime k}}-d_{1t_{i1}},{\sigma^{2}}^{\prime })$ or $\delta_{i\prime k}=d_{1t_{i\prime k}}-d_{1t_{i1}}$ , depending on random or fixed study effects, respectively. The disconnected RCT specific heterogeneity variance ${\sigma^{2}}^{\prime }$ is again potentially using $\sigma^{2}$ as an informative prior. There is no influence on $\mu_{i}^{matched}$ or on $\sigma^{2}$ for random study effects models, from the disconnected RCT, so randomization in connected RCT i is preserved. This procedure is therefore not equivalent to merging the connected and disconnected RCTs into a single RCT connected to the reference. Also, because the matching is performed at the level of the model, there is no multiple use of data from the RCT i as in reference prediction.

As the $\delta_{i\prime k}$ are relative to $t_{i1}$ and not the baseline of i′ , the correlation correction for RCTs with 3 or more arms under random study effects must be modified. We complete this modification in the appendix.

Similarity could be measured by the Euclidean distance ${\left(\sum_{l}^{n_{c}}\sqrt{x_{i\cdot l}-x_{i^{l}\cdot l}^{\prime }}\right)}^{2}$ over covariates $l=1,\ldots ,n_{c}$ , perhaps standardized by standard deviations to account for different scales, although any preferred distance measure can be used. Matching should also take account of similarity of endpoint definition, follow-up, permitted concomitant treatments, and other design characteristics, as in matching-adjusted indirect comparison.^34,35^ The same approach should be taken when matching RCTs already connected to the reference to single-arm studies.

**Figure 3:**
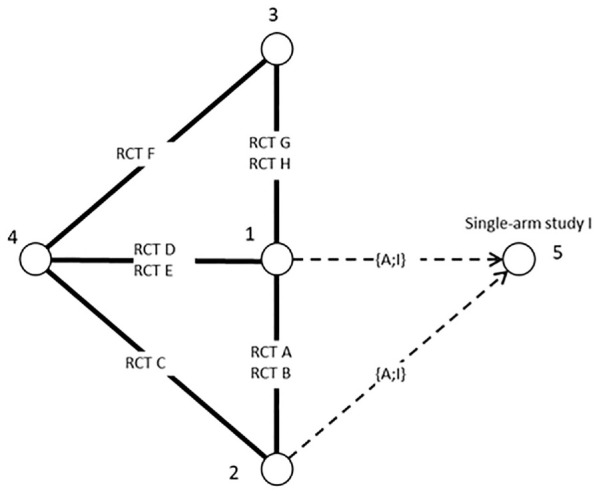
Using aggregate-level matching to connect a single-arm study to randomized controlled trials (RCTs) with similar design and patient characteristics. In this case, the baseline odds of RCT A is used to match to RCT I.

ALM has been previously applied to single-arm studies.7 As illustrated in Figure 3, the approach to single-arm studies i″ with the probability $p_{i\prime \prime }$ is similar, and we model as

$$logit\left(p_{i\prime \prime }\right)=\mu_{i}^{matched}+\delta_{i\prime \prime }$$

The connected RCT should have baseline characteristics $x_{i\cdot }$ , with · indicating an average overall arms k , most similar to the $x_{i\prime \cdot }^{\prime }$ , again averaged over all arms, of the disconnected RCT.

Unlike reference prediction, ALM requires only a single RCT connected to the reference to be identifiable, although more are recommended to ensure a sufficiently similar study is used for matching.

## Assessment by Constructed Data

### Methods of Constructed Data Example

Our constructed data example was created by artificially disconnecting a network drawn from a systematic literature review and contrast-based NMA on a connected network comparing treatments for the prevention of stroke, while minimizing the risk of clinically relevant bleeding, in atrial fibrillation.^25,36^ The advantage of artificially disconnecting a network is that we can compare the estimated of reference prediction and ALM to the results of the “true NMA” based on RCTs. Aligning with the methods presented in the sections “Existing Evidence Synthesis Models” and “Modified Evidence Synthesis Models for Disconnected Networks with Aggregate-Level Data,” we analyzed the binomial outcomes with a logistic link function. The NMA compared coumarin (international normalized ratio target range 2–3) with the oral anticoagulants apixaban (twice-daily 5 mg), dabigatran (twice-daily 150 mg), edoxaban (once-daily 60 mg), and rivaroxaban (once-daily 20 mg). This consists of 12 RCTs on 13 interventions reporting stroke and 17 RCTs on 24 interventions reporting clinically relevant bleeding.

**Figure 4:**
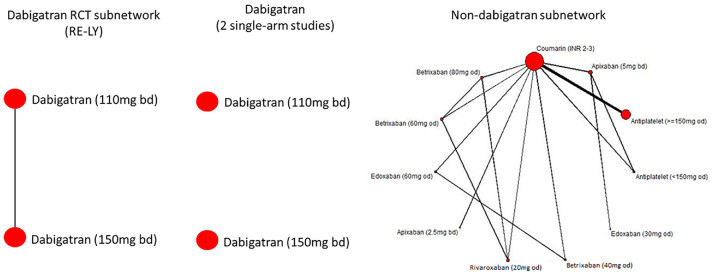
Disconnecting the ischemic stroke atrial fibrillation network. Twelve randomized controlled trials (RCTs) on 13 interventions are changed to either 1 RCT connected to the reference and 11 RCTs connected to the reference or to 2 single-arm studies and 11 RCTs to the reference.

We artificially disconnected the trials on dabigatran from those on other treatments in the evidence networks on stroke and clinically relevant bleeding. In the stroke network, there was 1 RCT on dabigatran, whereas in clinically relevant bleeding, there were 3 RCTs (details are given in the Appendix). In the first scenario, the coumarin arm was removed from the dabigatran trials, giving a disconnected network of 1 RCT on dabigatran 110 mg versus 150-mg doses in ischemic stroke and 3 RCTs on 9 different doses of dabigatran in clinically relevant bleeding. The second scenario consisting of splitting the RCTs on dabigatran into single-arm studies and excluding the coumarin control arms, giving 2 for ischemic stroke and 12 for clinically relevant bleeding. These disconnections are illustrated for ischemic stroke in Figure 4.

We then applied the reference prediction and ALM to these disconnected networks in an attempt to reproduce the results of the original connected network, the true NMA. For reference prediction, we used meta-regression to explore which, if any, of mean age, proportion male, and CHA_2_DS_2_-VASc score should be included in the baseline response model. Covariates were centered at the across-study mean, and missing values for individual studies were set to the mean (i.e., zero in the regression due to centering). All 3 covariates, with no standardization, were used to calculate the Euclidean distance ALM, with missing values omitted from the summation.

### Results of the Atrial Fibrillation Constructed Data Example

The random effects model with no covariates was selected as the regression model for reference prediction on ischemic stroke, because it had good total residual deviance (11.9 v. 11 data points), and its DIC of 67.14 was not substantially improved by including covariates (the DIC of the covariate models ranged from 66.96 to 67.93). All fixed effects had poor residual deviance (ranging from 68 to 79.8, which was substantially greater than the 11 data points) and worse DIC (ranging from 119 to 127.1) than the random effects models did. The same conclusions held for clinically relevant bleeding.

**Figure 5:**
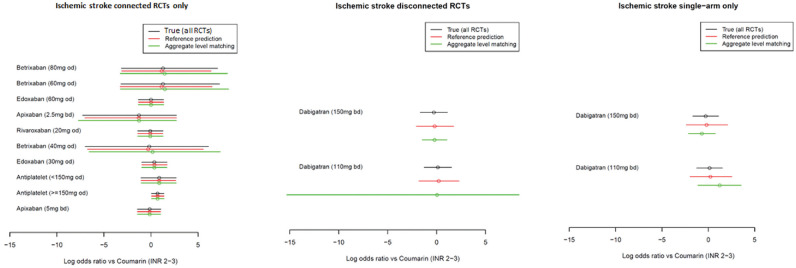
Comparison of the estimated log odds ratios using the true method (all randomized controlled trials), reference prediction, and aggregate-level matching under random study effects for the ischemic stroke outcome. Point estimates are means, and uncertainty intervals are 95% credible intervals.

**Figure 6:**
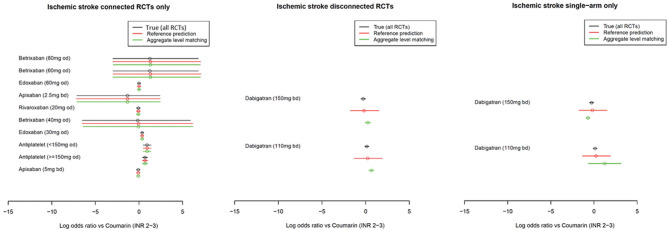
Comparison of the estimated log odds ratios using the true method (all randomized controlled trials), reference prediction, and aggregate-level matching under fixed study effects for the ischemic stroke outcome. Point estimates are means, and uncertainty intervals are 95% credible intervals.

The ischemic stroke results for the methods with random study effects are presented in Figure 5 and those for the fixed study effects are shown in Figure 6. The tabulated results are provided in the Appendix.

Using both the fixed and random study effects, we observed that the reference prediction and ALM reproduced the results of the true NMA (i.e., independent baselines using all RCTs) in the connected portion of the network. For example, the log odds ratio for edoxaban (30 mg every day) was 0.36 (95% credible interval [CrI]: −0.94, 1.64) using true NMA, 0.36 (−0.92, 1.63) using reference prediction, and 0.36 (−0.93, 1.61) using ALM.

For the reference prediction on RCTs disconnected from the reference and single-arm studies, the point estimates were close to those of the true NMA. For example, if in an RCT disconnected from the reference, the log odds ratios for dabigatran (150 mg) were −0.28 (−1.55, 0.99) under true NMA, −0.19 (−1.98, 1.71) using reference prediction, and −0.21 (−1.36, 0.94) using ALM. The 95% CrIs were similar under the random study effects but much wider under the fixed study effects; however, in both cases, they overlapped with the point estimate of the true NMA. ALM had poor performance under fixed study effects with different point estimates and narrow 95% credible intervals, which did not always overlap with the true NMA estimate; for example, if in RCTs disconnected from the reference dabigatran (110 mg), the log odds ratios were estimated as 0.13 (−0.11, 0.37) using true NMA and 0.22 (−1.34, 1.91) using reference prediction, but 0.66 (0.35, 0.96) using ALM. Under random study effects for single-arm studies, the point estimates appeared to be more different from the true NMA than the reference prediction but similar on the RCTs from the reference. The 95% CrIs also depend greatly on the matched study with, sometimes very wide CrIs; for example, in RCTs disconnected from the reference, ALM estimated the log odds ratio for dabigatran (110 mg) to be −0.0065 (−19.59, 19.68) but the true NMA estimated 0.13 (−1.14, 1.39) and reference prediction 0.23 (−1.70, 2.24).

Random study effects models for clinically relevant bleeding were less reassuring (results are given in the Appendix). Both reference prediction and ALM had 95% CrIs that often did not overlap with the true NMA effect for the PETRO study, which reported clinically relevant bleeding but not ischemic stroke, so only in the former outcome network. This is likely because PETRO was a small study with only 70 patients on coumarin, whereas RE-LY, in both outcome networks, included 6022 patients taking coumarin. Both methods performed better for dabigatran doses studied in RE-LY. In the disconnected RCTs scenario, ALM had point estimates closer to the truth than the reference prediction. Conversely, ALM estimates were further from the truth, and with 95% CrIs not overlapping the truth, for the single-arm scenario. Reference prediction appears to be “safer” in general. Again, fixed study effects models performed poorly.

## Assessment by Simulation Study

### Methods of Simulation Study

We used a simple simulation study to assess the bias and coverage of treatment effect estimates of ALM and reference prediction in the scenarios of disconnected RCTs (i.e., where there are RCTs disconnected from the reference) and single-arm studies. Methods with lower bias and greater coverage are preferred.

We merged all covariates for matching and regression in ALM and reference prediction, respectively, as a single covariate. We explain below how this does not reduce the generality of the simulations. We assumed the covariates were not treatment effect modifiers. Effect modifiers are a problem for both connected and disconnected networks, and our methods do not purport to overcome the imbalance in effect modifiers.

**Figure 7:**
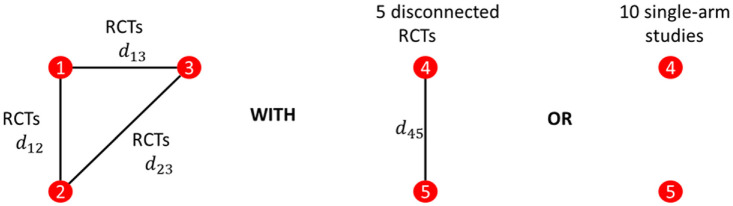
Network geometries considered in the simulation study. Either 5 randomized controlled trials (RCTs) from the reference or 10 single-are studies were considered. The number of RCTs to the reference was varied.

The basic geometry of all of our simulations is illustrated in Figure 7. The performance of ALM and reference prediction depend on the quantity of the connected evidence and not on the disconnected evidence. We therefore varied only the number of RCTs connected to the reference and always included only 5 RCTs disconnected from the reference or 10 single-arm studies. The number of 2-arm and 3-arm RCTs to the reference was gradually increased, with scenarios described in Table 1. Simulations were conducted twice, once with the number of patients in trials fixed at 100 on each arm and again with 1000 patients. The patient number simulations were kept separate, and our primary case included 100 patients per arm.

**Table 1:** Number of Randomized Control Trials (RCTs) in 3 Simulation Studies with 5, 15, and 50 RCTs Connected to the Reference^a^

Treatments to Compare	Total RCTs Connected to the Reference	Treatment 2 v. Treatment 1 (Reference)	Treatment 3 v. 1 (Reference)	Treatments 3 and 2 v. 1 (Reference)	RCTs Disconnected from the Reference (on Treatment 5 v. Treatment 4)	Single-Arm Studies on Treatment 4	Single-Arm Studies on Treatment 5
No. of RCTs	5	2	2	1	5	5	5
	15	5	5	5	5	5	5
	50	20	20	10	5	5	5

a The number and treatment arms of the RCTs disconnected from the reference and single-arm studies were the same in all simulations.

Our simulated data followed the setup of the binomial-logistic NMA example that has been used throughout. The number of events on arm k of study i was simulated as

$$r_{ik}\sim binomial\left(p_{ik},n_{ik}\right)$$

The probability of event $p_{ik}$ was modeled as

$$\mathrm{logit}\left(p_{ik}\right)=\mu_{i}+d_{1k}$$

For numerical stability, we forced any arms with zero events (r = 0) to have at least 1 event (r = 1). The log odds ratios $d_{1k}$ for $t_{ik}$ relative to reference $t_{i1}=1$ were simulated as $d_{1k}\sim N\left(0.5,1\right)$ for each treatment. This arbitrary choice of distribution determined the scale for other model parameters.

The model we used to simulate the log odds for study s was

$$\mu_{i}\sim Normal\left(m+X_{i}\beta +I_{i=disc}\gamma ,\mathrm{sd}=1\right)$$

We simulated the across-study mean from m~N(0.5,1) . The covariate effect $X_{i}$ represents variation in prognostic factors across studies and was simulated from $X_{i}\sim N\left(0.5,1\right)$ . β represents the strength of influence of $X_{i}$ , and we considered scenarios with weak and strong covariates simulated with β~N(0.1,1) and β~N(1,1) , respectively. A stronger influence suggests more data for the reference prediction model or value of matching on $X_{i}$ for ALM.

$I_{i=disc}$ is an indicator for i being a disconnected RCT or a single-arm study, whereas γ represents additional variation in baseline response in such studies. Together, these represent differences in prognostic variables between RCTs connected to the reference and either the RCTs from the reference or single-arm studies. A nonzero γ implies that reference prediction and ALM will be biased; thus, we considered scenarios γ=0 and γ~N(0.5,1) . This model for $\mu_{i}$ implies that the contrast-based NMA and the ALM and reference prediction models that match it on connected networks were misspecified for nonzero β and γ , as the NMA model assumes that $\mu_{i}$ was not systematically different between trials.

As noted above, we considered only a single prognostic factor $X_{i}$ , and this is without loss of generality. Unaccounted extras would be represented by scenarios with larger variation in m or a larger magnitude of γ , while accounted extras would just be represented by a stronger β .

We applied contrast-based NMA, reference prediction, and ALM for all modeled scenarios and compared their treatment effect estimates to the “truth” in the (for reference prediction and ALM only) components connected to or disconnected from the reference. As we ran these for both fixed and random effects, and for 100 and 1000 patients per study arm, there were 96 scenarios in total:

$$\begin{array}{c} \left(\begin{array}{c} Fixed\;effects\\ Random\;effects \end{array}\right)\times \left(\begin{array}{c} Disconnected\;RCTs\\ Single-arm\;studies \end{array}\right)\\ \times \left(\begin{array}{c} 100patients\;per\;arm\\ 1000patients\;per\;arm \end{array}\right)\times \left(\begin{array}{c} \beta weak\\ \beta strong \end{array}\right)\\ \times \left(\begin{array}{c} \gamma zero\\ \gamma strong \end{array}\right)\times \left(\begin{array}{c} 5connected\;RCTs\\ 15connected\;RCTs\\ 50connected\;RCTs \end{array}\right) \end{array}$$

The 12 scenarios across assumptions on β , γ , and the number of RCTs connected to the reference are listed in Table 2. For all simulations, we calculated the bias and coverage probability for log odds ratios for connected treatments ( $d_{12}$ and $d_{13}$ ) and disconnected treatments ( $d_{14}$ and $d_{15}$ ). The bias and coverage probability are explicitly defined in the Appendix. Also in the Appendix is a formal sample size calculation indicating that 100 simulations is sufficient to estimate the coverage probability to within a sufficiently small Monte Carlo standard error of 0.047 (i.e., within 4.7%).^37^ The Monte Carlo standard error of the bias is estimated to be 0.015, which is sufficient for estimating log odds ratios with distribution $d_{1k}\sim N\left(0.5,1\right)$ .

**Table 2:** List of Scenarios Used in the Simulation Study^a^

Scenario No.	Number of RCTs Connected to the Reference	Effect of Covariate Used in Regression or Matching β	Effect of Covariate Not Used in Regression or Matching γ
1	5 RCTs	Weak effect: β~N(0.1,1)	γ=0
2	5 RCTs	Strong effect: β~N(1,1)	γ=0
3	15 RCTs	Weak effect: β~N(0.1,1)	γ=0
4	15 RCTs	Strong effect: β~N(1,1)	γ=0
5	50 RCTs	Weak effect: β~N(0.1,1)	γ=0
6	50 RCTs	Strong effect: β~N(1,1)	γ=0
7	5 RCTs	Weak effect: β~N(0.1,1)	γ~N(0.1,1)
8	5 RCTs	Strong effect: β~N(1,1)	γ~N(0.1,1)
9	15 RCTs	Weak effect: β~N(0.1,1)	γ~N(0.1,1)
10	15 RCTs	Strong effect: β~N(1,1)	γ~N(0.1,1)
11	50 RCTs	Weak effect: β~N(0.1,1)	γ~N(0.1,1)
12	50 RCTs	Strong effect: β~N(1,1)	γ~N(0.1,1)

a Each is used under the assumption of fixed or random study effects and with either randomized controlled trials (RCTs) disconnected from the reference or single-arm studies, giving 48 scenarios in total.

### Results of the Simulation Study

**Figure 8:**
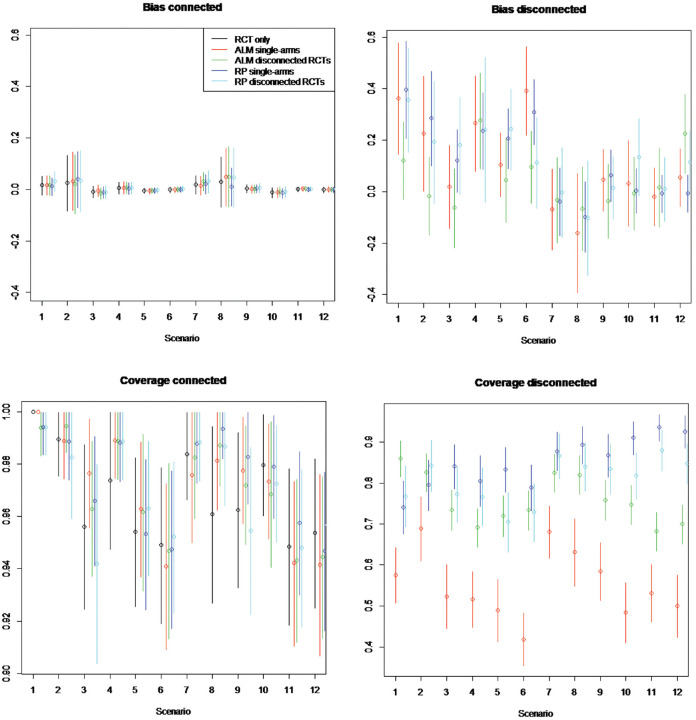
Simulation study estimated bias and coverage of each method in the connected and disconnected evidence scenarios. Random study effects of 100 patients per arm. Scenarios: scenario 1 = 5 randomized controlled trials (RCTs), β weak, γ = 0; scenario 2 = 5 RCTs, β strong, γ = 0; scenario 3 = 15 RCTs, β weak, γ = 0; scenario 4 = 15 RCTs, β strong, γ = 0; scenario 5 = 50 RCTs, β weak, γ = 0; scenario 6 = 50 RCTs, β strong, γ = 0; scenario 7 = 5 RCTs, β weak, γ≠ 0; scenario 8 = 5 RCTs, β strong, γ≠ 0; scenario 9 = 15 RCTs, β weak, γ≠ 0; scenario 10 = 15 RCTs, β strong, γ≠ 0; scenario 11 = 50 RCTs, β weak, γ≠ 0; scenario 12 = 50 RCTs, β strong, γ≠ 0. The β represents the impact of covariates included in aggregate-level matching (ALM) and reference prediction (RP) regression. γ represents the impact of the covariates not included in the ALM or RP regression. Scenarios are an analysis on RCTs only using single-arm studies (single) and RCTs from the reference (disconnected).

**Figure 9:**
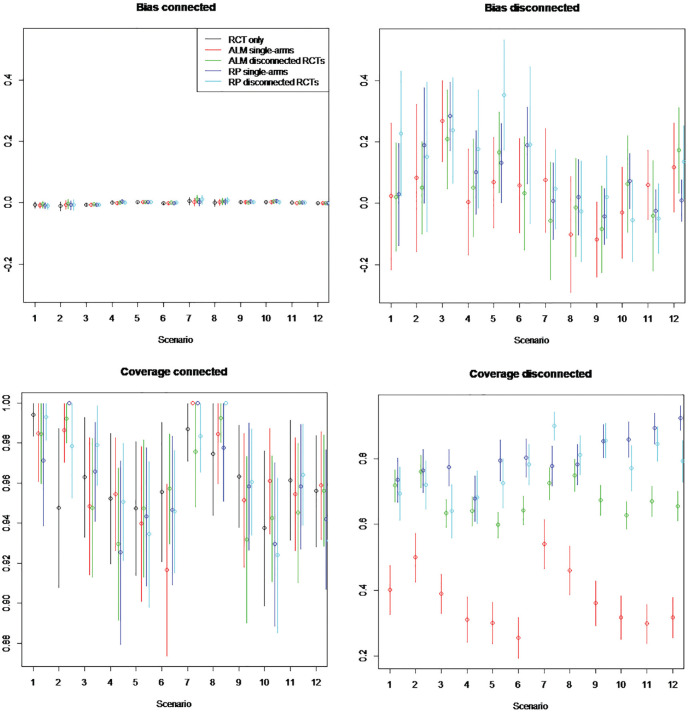
Simulation study estimated bias and coverage of each method in the connected and disconnected evidence scenarios. random study effects of 1000 patients per arm. Scenarios: scenario 1 = 5 randomized controlled trial (RCTs), β weak, γ = 0; scenario 2 = 5 RCTs, β strong, γ = 0; scenario 3 = 15 RCTs, β weak, γ = 0; scenario 4 = 15 RCTs, β strong, γ = 0; scenario 5 = 50 RCTs, β weak, γ = 0; scenario 6 = 50 RCTs, β strong, γ = 0; scenario 7 = 5 RCTs, β weak, γ≠ 0; scenario 8 = 5 RCTs, β strong, γ≠ 0; scenario 9 = 15 RCTs, β weak, γ≠ 0; scenario 10 = 15 RCTs, β strong, γ≠ 0; scenario 11 = 50 RCTs, β weak, γ≠ 0; scenario 12 = 50 RCTs, β strong, γ≠ 0. β represents the impact of covariates included in aggregate-level matching (ALM) and reference prediction (RP) regression. γ represents the impact of the covariates not included in ALM or RP regression. Scenarios are an analysis on RCTs only, using single-arm studies (single), and RCTs from the reference (disconnected).

Estimated bias and coverage probabilities across all methods and all scenarios for random study effects models in which RCTs have 100 patients are illustrated in Figure 8 and where RCTs have 1000 patients in Figure 9. Bias is interpreted in comparison with the mean treatment effect of 0.5 (i.e., the mean of log odds ratio $d_{1k}$ ). Numerical results and full results for fixed study effects models are provided in the Appendix.

### Estimated Bias

On the component of the network connected to the reference, the bias of ALM ranges from −0.0158 to 0.051, for reference prediction from −0.0107 to 0.083, and for NMA based only on RCTs from −0.011 to 0.030. This suggests the bias is lower for NMA only but is similar between ALM and reference prediction and in any case low (usually less than 10%) compared with the true treatment effect.

In RCTs disconnected from the reference and in single-arm studies, the bias is much higher, ranging from −0.1 to −0.4 when disconnected studies are not systematically different ( γ=0 ) and from −0.2 to 0.2 when they are systematically different ( γ≠0 ). These are substantial compared with the true mean treatment effect. Across scenarios, the bias of ALM ranges from −0.101 to 0.36 with 5 RCTs connected to the reference and from −0.040 to 0.392 with 50 RCTs, whereas that of reference prediction ranges from −0.102 to 0.400 with 5 RCTs and from −0.049 to 0.354 with 50 RCTs, suggesting no relation between bias and number of connected RCTs. Similarly, there is no clear trend with the strength of covariate effects ( β ). The bias of ALM ranges from −0.076 to 0.36 under weak covariate ( β ) effects and from −0.16 to 0.392 under strong covariate effects, while that of reference prediction ranges from −0.049 to 0.40 under weak effects and from −0.102 to 0.310 under strong effects. There is a marginal trend that a greater number of patients leads to lower bias for both methods. The bias of ALM ranges from −0.069 to 0.36 with 100 patients and from −0.117 to 0.173 with 1000 patients, while the bias of reference prediction ranges from −0.102 to 0.40 with 100 patients and −0.056 to 0.354. Overall, there is no trend of the bias being different between methods, with the bias of ALM ranging from −0.16 to 0.392 and that of reference prediction from −0.102 to 0.40.

### Estimated Coverage

In the component of the network connected to the reference, the coverage of all methods was similar but greatest for NMA using only RCTs. The coverage ranged for ALM from 0.92 to 1.00, for reference prediction from 0.92 to 1.00, and for NMA using only RCTs from 0.95 to 1.00.

In RCTs disconnected from the reference and in single-arm studies, reference prediction has a coverage ranging from 0.64 (1000 patients, disconnected RCTs, 15 connected RCTs, β weak, γ=0 ) to 0.94 (100 patients, single-arm studies, 50 connected RCTs, β weak, γ≠0 ). ALM has worse coverage, ranging from 0.30 (1000 patients, single-arm, 50 connected RCTs, β weak, γ=0 ) to 0.82 (100 patients, disconnected RCTs, 5 connected RCTs, β weak, γ≠0 ).

ALM appeared to perform worse for single-arm studies than for RCTs disconnected from the reference, with coverage on single-arm studies ranging from 0.26 to 0.69 and on disconnected RCTs from 0.60 to 0.86. Coverage of all methods appeared to decrease with increasing numbers of patients, going from a range of 0.42 to 0.95 on 100 patients to a range of 0.26 to 0.92 on 1000 patients. This may occur when there are systematic differences in prognostic factors between RCTs connected to the reference and single-arm studies or RCTs disconnected from the reference treatment (i.e., γ≠0 ), as ALM and reference prediction would become closer to the systematically different connected evidence. In this scenario, the coverage of all methods does indeed reduce from a range of 0.48 to 0.94 on 100 patients to a range of 0.36 to 0.92 on 1000 patients.

### Fixed Effects Results

Fixed study effects results are presented in the Appendix. Bias was generally similar to the random study effects case in both the components connected to and disconnected from the reference for all methods and scenarios. The coverage probabilities were lower for reference prediction on disconnected treatments and ranged from 0.81 to 1.00. Coverage probabilities were so low, ranging from 0.18 to 0.76 for disconnected treatments across all scenarios, for ALM that the method could not be recommended with fixed study effects. It is worth recalling from above that coverage was worst for ALM under random effects (from 0.30 to 0.82).

## Discussion

We have presented 2 modified methods for including RCTs disconnected from the reference, or any intervention of interest, or single-arm studies in contrast-based NMA when only aggregate data are available. Reference prediction is a modification of random effects on baseline with RCTs connected to the reference and RCTs disconnected from the reference kept separate, different heterogeneity variances used for random study effects models, and a corrected adjustment for multiarm studies. We modified ALM to allow the inclusion of RCTs from the reference, used different heterogeneity variances for RCTs connected to and disconnected from the reference, and corrected the adjustment for multiarm studies. These methods give similar point estimates and 95% CrIs to the independent baseline contrast-based NMA, which suggests that we have minimized the introduction of bias. Fixed study effects models performed poorly, with point estimates far from the truth and 95% CrIs that did not include the truth; this was matched by the simulation study in which fixed study effects had high bias and low coverage. The low coverage was a result of fixed effects being less “lenient” to poorly matching studies or to different populations than random effects. In the constructed data example, the performance of ALM was found to greatly depend on the matched study, illustrating the importance of choosing an appropriate study. The performance of reference prediction depended greatly on the similarity of the connected and disconnected evidence. This was confirmed by the simulation study in which coverage of both methods was worse when there were systematic differences in prognostic factors between connected and disconnected studies. This illustrates the importance of assessing whether these methods are appropriate in each specific circumstance. In both the constructed data and simulation study, reference prediction was found to have lower bias and greater coverage than ALM and could be viewed as the “safer” option for inference on disconnected networks.

There are many possible extensions to our methodology. Aggregate- or individual-level real-world evidence in the form of registries or cohort studies could be used to build the reference prediction models or control arm data for ALM.^11^ This could allow predictions to be tailored to specific populations, rather than relying only on what has been studied in an RCT. Both methods could be used to incorporate single-arm studies on treatments already in the RCTs connected to the reference; hierarchical models could be used to keep effects separate or informative priors used to minimize any bias from single-arm studies on RCT data.^7,38^ The high uncertainty in the effect estimates of random study effects reference prediction or ALM suggests that these may be better suited to uncertainty quantification for input in value-of-information analyses using economic models.^39^ This could provide an upper bound on the value of studying disconnected treatments in new head-to-head RCTs.

Equally, there are many limitations to our methods and assessments. Reference prediction, despite having good coverage and low bias, can provide highly variable and almost noninformative treatment effect estimates. Our constructed data situation was based on an example in atrial fibrillation and is unlikely to be generalizable to other clinical areas; that single-arm studies and RCTs from the reference were based on RCTs connected to the reference also means they are likely to be more similar in prognostic factors than a real scenario. Our constructed data example and simulation study have shown only that there are no problems in specific situations, and there may be cases in which both reference prediction and ALM lead to serious bias. We also restricted our analysis to binomial outcomes. Extension to count or continuous outcomes would be straightforward as similar generalized linear models for NMA on single time points exist.^1^ However, time-to-event outcomes NMA such as fractional polynomials NMA, common in oncology, would require more significant modification.^40^

We have presented methods that aim to get as close as possible to the results of a fully connected contrast-based NMA with independent baselines. However, we would term these as “last resort” methodologies for when networks are disconnected or if the RCT evidence is so limited as to be uninformative. If individual patient data are available from some or all trials, methods such as multilevel network meta-regression, propensity score reweighting, matching-adjusted indirect comparison, or simulated treatment comparison are recommended instead. If high-quality nonrandomized comparative evidence is available, this should instead be used through hierarchical models or informative priors. If only aggregate data from RCTs are available, and multiple outcomes, class effects, or dose-response modeling are not appropriate, reference prediction and ALM could be considered for health care decision making, as long as the limitations are recognized.

## Supplemental Material

sj-docx-1-mdm-10.1177_0272989X221097081 – Supplemental material for Network Meta-analysis on Disconnected Evidence Networks When Only Aggregate Data Are Available: Modified Methods to Include Disconnected Trials and Single-Arm Studies while Minimizing Bias Click here for additional data file. Supplemental material, sj-docx-1-mdm-10.1177_0272989X221097081 for Network Meta-analysis on Disconnected Evidence Networks When Only Aggregate Data Are Available: Modified Methods to Include Disconnected Trials and Single-Arm Studies while Minimizing Bias by Howard Thom, Joy Leahy and Jeroen P. Jansen in Medical Decision Making

sj-docx-2-mdm-10.1177_0272989X221097081 – Supplemental material for Network Meta-analysis on Disconnected Evidence Networks When Only Aggregate Data Are Available: Modified Methods to Include Disconnected Trials and Single-Arm Studies while Minimizing Bias Click here for additional data file. Supplemental material, sj-docx-2-mdm-10.1177_0272989X221097081 for Network Meta-analysis on Disconnected Evidence Networks When Only Aggregate Data Are Available: Modified Methods to Include Disconnected Trials and Single-Arm Studies while Minimizing Bias by Howard Thom, Joy Leahy and Jeroen P. Jansen in Medical Decision Making
