# Supplementary material for: Network Meta-analysis on Disconnected Evidence Networks When Only Aggregate Data Are Available: Modified Methods to Include Disconnected Trials and Single-Arm Studies while Minimizing Bias
Source: Med Decis Making. 2022 May 7;42(7):906–22. doi: 10.1177/0272989X221097081 (PMC9459361; doi:10.1177/0272989X221097081)
Supplement: sj-docx-1-mdm-10.1177_0272989X221097081 – Supplemental material for Network Meta-analysis on Disconnected Evidence Networks When Only Aggregate Data Are Available: Modified Methods to Include Disconnected Trials and Single-Arm Studies while Minimizing Bias [file sj-docx-1-mdm-10.1177_0272989X221097081.docx]

# Code appendix

All code runs in the R statistical programming language linked to OpenBUGS using the R2OpenBUGS library. Full code and data for the constructed data example are available at <https://github.com/Bogdasayen/disconnectednma-constructed-data>. Full code and data for the simulation study are available at <https://github.com/Bogdasayen/disconnectednma-simulation-study>. Analysis of a simple dataset is provided below. This is also available in a project, which will be easier to load into RStudio, at the repository <https://github.com/Bogdasayen/disconnectednma-simple-example>.

The script disconnected.nma.main.R calls data loading and model scripts to run independent baselines, ALM and reference prediction on an example evidence network. The evidence network is one draw from the simulation study with 5 connected RCTs with 100 patients per arm on treatments 1 and 2 and 3, 5 disconnected RCTs on treatments 4 and 5, and 10 single-arm studies with 5 on treatments 4 and 5 each. Data and code are described with in-line comments.

## disconnected.nma.main.R

# Code to implement reference prediction and ALM

# Load necessary libraries

require(R2OpenBUGS)

# Load necessary data

source("load.data.R")

# Global option to use random study effects or not

# Paper establishes that random study effects are preferred as base case

random.effects <- TRUE

# BUGS options

n.chains <- 2 # Code below (setting initial values) only set up for 2 but can be extended

num.sims <- 1000 # Recommend at least 10000 and ideally use n.thin = 2 or more

burn.in <- 1000 * n.chains # Recommend at least 30000

do.debug = FALSE # Set this to TRUE to see OpenBUGS running and check errors

##############################################################################

## Load models ############################################################

##############################################################################

# Standard independent baseline models with fixed and random study effects

# The following are the NICE TSD2 1c (RE) and 1d (FE) functions

source("independent.baselines.model.R")

# Reference prediction with fixed study effects to include single-arm studies

# The following use fixed effects with (up to) 3 covariates on baseline to include single arm studies and keeps RCT network otherwise separate from single-arm studies

source("reference.prediction.model.single.1cov.fe.R")

# Reference prediction with fixed study effects to include disconnected RCTs

# The following uses fixed effects with (up to) 1 covariate on baseline

source("reference.prediction.model.disc.1cov.fe.R")

# Random effects models for reference prediction and ALM have to use a model file

# format in order to use the dnorm(,)I(,) syntax to truncate the prior on sd

# in disconnected evidence

# Reference prediction with random study effects to include single-arm studies

model.file.reference.prediction.single.1cov.re <- "reference.prediction.model.single.1cov.re.txt"

# Reference prediction with random study effectst to include disconnected RCTs

model.file.reference.prediction.disc.1cov.re <- "reference.prediction.model.disc.1cov.re.txt"

# ALM models fixed study effects to include single-arm studies

source("alm.model.single.fe.R")

# ALM models fixed study effects to include disconnected RCTs

source("alm.model.disc.fe.R")

# ALM models random study effects to include single-arm studies

model.file.alm.single.re <- "alm.model.single.re.txt"

# ALM models random study effects to include disconnected RCTs

model.file.alm.disc.re <- "alm.model.disc.re.txt"

##############################################################################

## Initial values ############################################################

##############################################################################

# Initial values are shared across all models

# Some values are simply unused by some models

inits1<-list(d = c(NA, rep(0.5,b.data$nt-1)), mu=rep(0.5,b.data$ns), sd = 1,

mu.single = rep(-0.5, b.data$ns.single), m = 0.1, sd.m = 1, beta = 0.1,

mu.base = rep(-0.5, b.data$ns.base), beta.base = 0.1,

mu.disc = matrix(-0.5, nrow=b.data$ns.disc, ncol=max(b.data$na.disc)),

sd = 1) # mu.disc is a vector in ALM and matrix in RP

inits2<-list(d = c(NA, rep(-0.5,b.data$nt-1)), mu=rep(-0.5,b.data$ns), sd = 0.5,

mu.single = rep(0.5, b.data$ns.single), m = 0.5, sd.m = 0.5, beta = 0.25,

mu.base = rep(0.5, b.data$ns.base), beta.base = 0.25,

mu.disc = matrix(0.5, nrow=b.data$ns.disc, ncol=max(b.data$na.disc)),

sd = 0.5) # mu.disc is a vector in ALM and matrix in RP

bugs.inits<-list(inits1,inits2)

##############################################################################

## Run independent baseline models ###########################################

##############################################################################

# Independent baseline fixed study effects

if(!random.effects) {

bugs.object.independent.baselines<-

bugs(data=b.data,inits=bugs.inits,parameters.to.save=c("mu","d"),model=model.independent.baseline.fe,

clearWD=TRUE,summary.only=FALSE,n.iter=(num.sims+burn.in),n.burnin=burn.in,n.chains=n.chains,bugs.seed=1,n.thin=1,debug=do.debug)

}

# Independent baseline random study effects

if(random.effects) {

bugs.object.independent.baselines <-

bugs(data=b.data,inits=bugs.inits,parameters.to.save=c("mu","d","sd"),model=model.independent.baseline.re,

clearWD=TRUE,summary.only=FALSE,n.iter=(num.sims+burn.in),n.burnin=burn.in,n.chains=n.chains,bugs.seed=1,n.thin=2,debug=do.debug)

}

##############################################################################

## Match studies for ALM #####################################################

##############################################################################

# Vectors of RCTs matched to the disconnected RCTs and single-arm studies

# Used for plug-in models

matched.rct.disc <- rep(NA,b.data$ns.disc)

matched.rct.single <- rep(NA, b.data$ns.single)

# For ALM, need to extract the plug in for the closest matching study

# Need separate data structures for single-arm and disconnected analyses for the plug-in models

b.data.disc <- b.data.single <- b.data

for(i.disc in 1:b.data$ns.disc)

{

# Use euclidean distance to choose closest arm match

distance <- rep(NA, b.data$ns)

for(i.rct in 1:b.data$ns)

{

# Distance between unweighted average of arms of each trial

distance[i.rct] <- dist(rbind(

mean(b.data$x.disc[i.disc,1:b.data$na.disc[i.disc]]),

mean(b.data$x[i.rct,1:b.data$na[i.rct]])

))

}

min.rct<-which.min(distance)

matched.rct.disc[i.disc]<-min.rct

}

for(i.single in 1:b.data$ns.single)

{

# Use euclidean distance to choose closest arm match

distance <- rep(NA, b.data$ns)

for(i.rct in 1:b.data$ns)

{

# Distance between unweighted average of arms of each trial

distance[i.rct] <- dist(rbind(

b.data$x.single[i.single],

mean(b.data$x[i.rct,1:b.data$na[i.rct]])

))

}

min.rct<-which.min(distance)

matched.rct.single[i.single]<-min.rct

}

b.data.single$matched.rct <- matched.rct.single

b.data.disc$matched.rct <- matched.rct.disc

# Take the mu from the matched RCT

# Adding NA at end to avoid confusion between vectors and scalars

b.data.single$mu.plugin.mean<-

c(bugs.object.independent.baselines$summary[matched.rct.single,"mean"],NA)

b.data.single$mu.plugin.prec<-

1/c(bugs.object.independent.baselines$summary[matched.rct.single,"sd"],NA)^2

b.data.disc$mu.plugin.mean<-

c(bugs.object.independent.baselines$summary[matched.rct.disc,"mean"],NA)

b.data.disc$mu.plugin.prec<-

1/c(bugs.object.independent.baselines$summary[matched.rct.disc,"sd"],NA)^2

##############################################################################

## Run reference prediction and ALM ##########################################

##############################################################################

# And informative priors for sd.disc from the connected RCTs

# This is for both plug-in and reference prediction models

if(random.effects) {

b.data.disc$sd.connected.mean<-b.data.single$sd.connected.mean<-b.data$sd.connected.mean<-

bugs.object.independent.baselines$summary["sd","mean"]

b.data.disc$sd.connected.tau<-b.data.single$sd.connected.tau<-b.data$sd.connected.tau<-

1/bugs.object.independent.baselines$summary["sd","sd"]^2

}

# Reference prediction with 1 covariate for disconnected studies

if(!random.effects) {

bugs.object.reference.prediction.disc.fe <-

bugs(data=b.data,inits=bugs.inits,parameters.to.save=c("d"),model=model.reference.prediction.disc.1cov.fe,

clearWD=TRUE,summary.only=FALSE,n.iter=(num.sims+burn.in),n.burnin=burn.in,n.chains=n.chains,bugs.seed=1,n.thin=1,debug=do.debug)

} else {

bugs.object.reference.prediction.disc.re <-

bugs(data=b.data,inits=bugs.inits,parameters.to.save=c("d"),model.file= model.file.reference.prediction.disc.1cov.re,

clearWD=TRUE,summary.only=FALSE,n.iter=(num.sims+burn.in),n.burnin=burn.in,n.chains=n.chains,bugs.seed=1,n.thin=2,debug=do.debug)

}

# Reference prediction with 1 covariate for single-arm studies

if(!random.effects) {

bugs.object.reference.prediction.single.fe <-

bugs(data=b.data,inits=bugs.inits,parameters.to.save=c("d"),model=model.reference.prediction.single.1cov.fe,

clearWD=TRUE,summary.only=FALSE,n.iter=(num.sims+burn.in),n.burnin=burn.in,n.chains=n.chains,bugs.seed=2,n.thin=1,debug=do.debug)

} else {

bugs.object.reference.prediction.single.re <-

bugs(data=b.data,inits=bugs.inits,parameters.to.save=c("d"),model.file=model.file.reference.prediction.single.1cov.re,

clearWD=TRUE,summary.only=FALSE,n.iter=(num.sims+burn.in),n.burnin=burn.in,n.chains=n.chains,bugs.seed=2,n.thin=2,debug=do.debug)

}

# ALM disconnected RCTs

if(!random.effects) {

bugs.object.alm.disc.fe <-

bugs(data=b.data.disc,inits=bugs.inits,parameters.to.save=c("d"),model=model.alm.disc.fe,

clearWD=TRUE,summary.only=FALSE,n.iter=(num.sims+burn.in),n.burnin=burn.in,n.chains=n.chains,bugs.seed=1,n.thin=1,debug=do.debug)

} else {

bugs.object.alm.disc.re <-

bugs(data=b.data.disc,inits=bugs.inits,parameters.to.save=c("d"),model.file=model.file.alm.disc.re,

clearWD=TRUE,summary.only=FALSE,n.iter=(num.sims+burn.in),n.burnin=burn.in,n.chains=n.chains,bugs.seed=1,n.thin=2,debug=do.debug)

}

# ALM single-arm studies

if(!random.effects) {

bugs.object.alm.single.fe <-

bugs(data=b.data.single,inits=bugs.inits,parameters.to.save=c("d"),model=model.alm.single.fe,

clearWD=TRUE,summary.only=FALSE,n.iter=(num.sims+burn.in),n.burnin=burn.in,n.chains=n.chains,bugs.seed=1,n.thin=1,debug=do.debug)

} else {

bugs.object.alm.single.re <-

bugs(data=b.data.single,inits=bugs.inits,parameters.to.save=c("d"),model.file=model.file.alm.single.re,

clearWD=TRUE,summary.only=FALSE,n.iter=(num.sims+burn.in),n.burnin=burn.in,n.chains=n.chains,bugs.seed=1,n.thin=2,debug=do.debug)

}

## load.data.R

b.data <- list()

# Data on connected RCTs

# Number of studies

b.data$ns <- 5

# Number of treatemnts

b.data$nt <- 5

# Number of events in the 5 connected RCTs (4 have 2 arms, 1 has 3 arms)

b.data$r <- matrix(NA, nrow = 5, ncol = 3)

b.data$r[1,] <- c(94, 92, NA)

b.data$r[2,] <- c(98, 96, NA)

b.data$r[3,] <- c(98, 96, NA)

b.data$r[4,] <- c(95, 97, NA)

b.data$r[5,] <- c(94, 98, 96)

# Number of patients on each arm

b.data$n <- matrix(NA, nrow = 5, ncol =3)

b.data$n[1,] <- c(100, 100, NA)

b.data$n[2,] <- c(100, 100, NA)

b.data$n[3,] <- c(100, 100, NA)

b.data$n[4,] <- c(100, 100, NA)

b.data$n[5,] <- c(100, 100, 100)

# Treatments of each arm of each of the 5 RCTs

b.data$t <- matrix(NA, nrow = 5, ncol =3)

b.data$t[1,] <- c(1, 2, NA)

b.data$t[2,] <- c(1, 2, NA)

b.data$t[3,] <- c(1, 3, NA)

b.data$t[4,] <- c(1, 3, NA)

b.data$t[5,] <- c(1, 2, 3)

# Covariate value on each arm of each RCTRs

b.data$x <- matrix(NA, nrow = 5, ncol =3)

b.data$x[1,] <- c(-0.1336573, -0.2878004, NA)

b.data$x[2,] <- c(0.9109553, -0.1664376, NA)

b.data$x[3,] <- c(0.6923338, 0.2929722, NA)

b.data$x[4,] <- c(1.3306355, 1.1880444, NA)

b.data$x[5,] <- c(1.3141482, 1.2933084, 1.700588)

# Number of arms in 5 RCTs of connected network

b.data$na <- c(2, 2, 2, 2, 3)

# Data for reference/baseline treatments

# Number of arms in connected network with reference treatment

# In this case all 5 RCTs included the reference treatment

b.data$ns.base <- 5

# Number of events in arms on reference treatment

b.data$r.base <- c(94, 98, 98, 95, 94)

# Number of patients in arms on reference treatment

b.data$n.base <- c(100, 100, 100, 100, 100)

# Covariate values in arms on reference treatment

# Corresponds to first column of x[] matrix in connected RCTs

b.data$x.base <- c(-0.1336573, 0.9109553, 0.6923338, 1.3306355, 1.3141482)

# Average value of covariate in arms on reference treatment

b.data$x.base.mean <- c(0.8228831)

# Data for single-arm studies to be included using ALM or reference prediction

# Number of single-arm studies

b.data$ns.single <- 10

# Number of events on single-arm studies

b.data$r.single <- c(100, 96, 98, 87, 99, 91, 36, 78, 99, 83)

# Number of patients on single-arm studies

b.data$n.single <- c(100, 100, 100, 100, 100, 100, 100, 100, 100, 100)

# Treatments of single-arm studies

b.data$t.single <- c(4, 4, 4, 4, 4, 5, 5, 5, 5, 5)

# Covariate values for single-arm studies

b.data$x.single <- c(-0.43956105, -0.36811619, 0.71098340, -0.13441955, 0.26357280, 2.25849534,

0.10642150, -0.06012546, 1.29700372, 0.63297998)

# Data for disconnected RCTs to include using either ALM or reference prediction

# Number of disconnected RCTs

b.data$ns.disc <- 5

# Number of events on arms of disconnected RCTs

b.data$r.disc <- matrix(NA, nrow = 5, ncol = 2)

b.data$r.disc[1,] <- c(99, 100)

b.data$r.disc[2,] <- c(96, 100)

b.data$r.disc[3,] <- c(90, 78)

b.data$r.disc[4,] <- c(98, 90)

b.data$r.disc[5,] <- c(96, 95)

# Number of patients on arms of disconnected RCTs

b.data$n.disc <- matrix(NA, nrow = 5, ncol = 2)

b.data$n.disc[1,] <- c(100, 100)

b.data$n.disc[2,] <- c(100, 100)

b.data$n.disc[3,] <- c(100, 100)

b.data$n.disc[4,] <- c(100, 100)

b.data$n.disc[5,] <- c(100, 100)

# Treatments for arms of disconnected RCTs

b.data$t.disc <- matrix(NA, nrow = 5, ncol = 3)

b.data$t.disc[1,] <- c(4, 5, NA)

b.data$t.disc[2,] <- c(4, 5, NA)

b.data$t.disc[3,] <- c(4, 5, NA)

b.data$t.disc[4,] <- c(4, 5, NA)

b.data$t.disc[5,] <- c(4, 5, NA)

# Covariate values for arms of disconnected RCTs

b.data$x.disc <- matrix(NA, nrow = 5, ncol = 2)

b.data$x.disc[1,] <- c(2.2867433, 1.20999082)

b.data$x.disc[2,] <- c(1.5475021, 1.01273989)

b.data$x.disc[3,] <- c(1.1266772, 0.23397848)

b.data$x.disc[4,] <- c(0.2853316, 0.08022455)

b.data$x.disc[5,] <- c(1.8597435, 0.31436530)

# Number of arms in disconnected RCTs

b.data$na.disc <- c(2, 2, 2, 2, 2)

## independent.baselines.model.R

## Model using independent effects on the baseline

# Binomial likelihood, logit link

# Simultaneous baseline and treat effects model for multi-arm trials

model.independent.baseline.re<-function()

{ # *** PROGRAM STARTS

for(i in 1:ns){ # LOOP THROUGH STUDIES

w[i,1] <- 0 # adjustment for multi-arm trials is zero for control arm

delta[i,1] <- 0 # treatment effect is zero for control arm

mu[i] ~ dnorm(0,0.01) # model for trial baselines re treatment 1

for (k in 1:na[i]) { # LOOP THROUGH ARMS

r[i,k] ~ dbin(p[i,k],n[i,k]) # binomial likelihood

logit(p[i,k]) <- mu[i] + delta[i,k] # model for linear predictor

rhat[i,k] <- p[i,k] * n[i,k] # expected value of the numerators

dev.NA[i,k] <- 2 * (r[i,k] * (log(r[i,k])-log(rhat[i,k])) #Deviance contribution including NAs

+ (n[i,k]-r[i,k]) * (log(n[i,k]-r[i,k]) - log(n[i,k]-rhat[i,k])))

dev[i,k] <- dev.NA[i,k]*(1-equals(n[i,1],1)) #Deviance contribution with correction for NAs

}

resdev[i] <- sum(dev[i,1:na[i]]) # summed residual deviance contribution for this trial

for (k in 2:na[i]) { # LOOP THROUGH ARMS

delta[i,k] ~ dnorm(md[i,k],taud[i,k]) # trial-specific LOR distributions

md[i,k] <- d[t[i,k]] - d[t[i,1]] + sw[i,k] # mean of LOR distributions (with multi-arm trial correction)

taud[i,k] <- tau *2*(k-1)/k # precision of LOR distributions (with multi-arm trial correction)

w[i,k] <- (delta[i,k] - d[t[i,k]] + d[t[i,1]]) # adjustment for multi-arm RCTs

sw[i,k] <- sum(w[i,1:k-1])/(k-1) # cumulative adjustment for multi-arm trials

}

}

totresdev <- sum(resdev[]) # Total Residual Deviance

d[1]<-0 # treatment effect is zero for reference treatment

for (k in 2:nt){ d[k] ~ dnorm(0,.01) } # vague priors for treatment effects

sd ~ dunif(0,2) # vague prior for between-trial SD

tau <- pow(sd,-2) # between-trial precision = (1/between-trial variance)

}

# The independent effects on baseline and fixed treatment effect model

model.independent.baseline.fe<-function()

{

for(i in 1:ns){ # LOOP THROUGH STUDIES

mu[i] ~ dnorm(0,.01) # vague priors for all trial baselines

for (k in 1:na[i]) { # LOOP THROUGH ARMS

r[i,k] ~ dbin(p[i,k],n[i,k]) # Binomial likelihood

logit(p[i,k]) <- mu[i] + delta[i,k]

delta[i,k]<-d[t[i,k]] - d[t[i,1]] # model for linear predictor

rhat[i,k] <- p[i,k] * n[i,k] # expected value of the numerators

dev[i,k] <- 2 * (r[i,k] * (log(r[i,k])-log(rhat[i,k]))

+ (n[i,k]-r[i,k]) * (log(n[i,k]-r[i,k]) - log(n[i,k]-rhat[i,k]))) #Deviance contribution

}

resdev[i] <- sum(dev[i,1:na[i]]) # summed residual deviance contribution for this trial

}

totresdev <- sum(resdev[]) #Total Residual Deviance

d[1]<-0 # treatment effect is zero for reference treatment

for (k in 2:nt){ d[k] ~ dnorm(0,.01) } # vague priors for treatment effects

}

## reference.prediction.model.single.1cov.fe.R

# Reference prediction fixed study effects to include single-arm studies

# Data are same as TSD format: ns, nt, na, r, n, t

# The data on baseline arms (from RCTs) are

# ns.base : number of baseline arms

# r.base : number of events in baseline arms

# n.base : number of patients in baseline arms

# x.base : Matrix of covariates with one row per covariate

# The data on single arm studies are:

# ns.single : number of single arm studies

# r.single : number of events in single arm studies

# n.single : number of patients in single arm studies

# t.single : treatment in single arm study

# x.single : matrix of covariates with one row per covariate

# cov.index : vector of numbers indicating which row of x.single/x.base to use for each covariate

# NOTE: All x.base and x.single must be defined. Suggest using mean covariate value when not reported.

# NOTE: Naively pools single arm and RCT evidence on treatment effects (not sure enough evidence to fit hierarchical model)

# Reference prediction fixed study effects with 1 covariate including single-arm studies

# Binomial likelihood, logit link

# Simultaneous baseline and treatment effects model for multi-arm trials

model.reference.prediction.single.1cov.fe<-function()

{

# Model for RCTs ###################################

for(i in 1:ns){ # LOOP THROUGH STUDIES

mu[i] ~ dnorm(0,0.01) # random effect on baselines

for (k in 1:na[i]) { # LOOP THROUGH ARMS

r[i,k] ~ dbin(p[i,k],n[i,k]) # Binomial likelihood

logit(p[i,k]) <- mu[i] + delta[i,k]

delta[i,k]<-d[t[i,k]] - d[t[i,1]] # model for linear predictor

rhat[i,k] <- p[i,k] * n[i,k] # expected value of the numerators

dev[i,k] <- 2 * (r[i,k] * (log(r[i,k])-log(rhat[i,k]))

+ (n[i,k]-r[i,k]) * (log(n[i,k]-r[i,k]) - log(n[i,k]-rhat[i,k]))) #Deviance contribution

}

resdev[i] <- sum(dev[i,1:na[i]]) # summed residual deviance contribution for this trial

}

# Model for baseline effects ###################################

# Adapted from Program 1 of NICE DSU TSD 5

for (i in 1:ns.base){ # LOOP THROUGH STUDIES

r.base[i] ~ dbin(p.base[i],n.base[i]) # Likelihood

logit(p.base[i]) <- mu.base[i]

mu.base[i] ~ dnorm(mu.base.mean[i],tau.m) # Random effects model

mu.base.mean[i] <- m + (x.base[i]-x.base.mean)*beta.base # Prediction of mean effect # Log-odds of response

}

beta.base~dnorm(0,0.01) # vague prior for covariate effects

m ~ dnorm(0,.01) # vague prior for mean

var.m <- 1/tau.m # between-trial variance

tau.m <- pow(sd.m,-2) # between-trial precision = (1/between-trial variance)

sd.m ~ dunif(0,5) # vague prior for between-trial SD

# Model for single-arm studies ###################################

# Prevent feedback to baseline model (these two may not be necessary as mu.single is cut below

m.cut<-cut(m)

tau.m.cut<-cut(tau.m)

beta.base.cut<-cut(beta.base)

for(i in 1:ns.single)

{

r.single[i]~dbin(p.single[i],n.single[i])

logit(p.single[i]) <- mu.single[i]

mu.single[i] ~ dnorm(mu.single.mean[i], tau.m.cut)

mu.single.mean[i] <- m.cut + delta.single[i] + (x.single[i]-x.base.mean)*beta.base.cut # model for linear predictor

delta.single[i]<-d[t.single[i]]

rhat.single[i] <- p.single[i] * n.single[i] # expected value of the numerators

dev.single[i] <- 2 * (r.single[i] * (log(r.single[i])-log(rhat.single[i]))

+ (n.single[i]-r.single[i]) * (log(n.single[i]-r.single[i]) - log(n.single[i]-rhat.single[i]))) #Deviance contribution

}

# Calculate deviance ###################################

totresdev.single<-sum(dev.single[]) # Total residual deviance for single-arm studies

totresdev.rct <- sum(resdev[]) # Total residual deviance for RCTs

totresdev<-totresdev.single+totresdev.rct #Total Residual Deviance

# Priors for remaining parameters

d[1]<-0 # treatment effect is zero for reference treatment

for (k in 2:nt){ d[k] ~ dnorm(0,.01) } # vague priors for treatment effects

}

## reference.prediction.model.disc.1cov.fe.R

# Reference prediction models with fixed study effects to include disconnected RCTs

# Data are same as TSD format: ns, nt, na, r, n, t

# The data on baseline arms (from RCTs) are

# ns.base : number of baseline arms

# r.base : number of events in baseline arms

# n.base : number of patients in baseline arms

# x.base : Matrix of covariates with one row per covariate

# The data on disconnected networks are (as in standard TSD):

# ns.disc, nt.disc, na.disc, r.disc, n.disc, t.disc

# x.disc is matrix of covariates for disconnected RCTs.

# cov.index is the index of the covariates to use in x.disc and x.base

# NOTE: All x.base and x.disc must be defined. Suggest using mean covariate value when not reported.

# NOTE: Naively pools disconnected and connected RCT evidence on treatment effects (not sure enough evidence to fit hierarchical model)

# Reference prediction with fixed study effect model to include disconnected RCTs

# Binomial likelihood, logit link

# Simultaneous baseline and treat effects model for multi-arm trials

model.reference.prediction.disc.1cov.fe<-function()

{

# Model for RCTs ###################################

for(i in 1:ns){ # LOOP THROUGH STUDIES

mu[i] ~ dnorm(0,0.01) # random effect on baselines

for (k in 1:na[i]) { # LOOP THROUGH ARMS

r[i,k] ~ dbin(p[i,k],n[i,k]) # Binomial likelihood

logit(p[i,k]) <- mu[i] + delta[i,k]

delta[i,k]<-d[t[i,k]] - d[t[i,1]] # model for linear predictor

rhat[i,k] <- p[i,k] * n[i,k] # expected value of the numerators

dev[i,k] <- 2 * (r[i,k] * (log(r[i,k])-log(rhat[i,k]))

+ (n[i,k]-r[i,k]) * (log(n[i,k]-r[i,k]) - log(n[i,k]-rhat[i,k]))) #Deviance contribution

}

resdev[i] <- sum(dev[i,1:na[i]]) # summed residual deviance contribution for this trial

}

# Model for baseline effects ###################################

# Adapted from Program 1 of NICE DSU TSD 5

for (i in 1:ns.base){ # LOOP THROUGH STUDIES

r.base[i] ~ dbin(p.base[i],n.base[i]) # Likelihood

logit(p.base[i]) <- mu.base[i]

mu.base[i] ~ dnorm(mu.base.mean[i],tau.m) # Random effects model

mu.base.mean[i] <- m + (x.base[i]-x.base.mean)*beta.base # Prediction of mean effect # Log-odds of response

}

beta.base~dnorm(0,0.01) #0.298 # vague prior for covariate effects

m ~ dnorm(0,.01) # vague prior for mean

var.m <- 1/tau.m # between-trial variance

tau.m <- pow(sd.m,-2) # between-trial precision = (1/between-trial variance)

sd.m ~ dunif(0,2) # vague prior for between-trial SD

# Model for disconnected RCTs ###################################

# Prevent feedback to baseline model (these two may not be necessary as mu.single is cut below

m.cut<-cut(m)

tau.m.cut<-cut(tau.m)

beta.base.cut<-cut(beta.base)

for(i in 1:ns.disc){ # LOOP THROUGH STUDIES

for (k in 1:na.disc[i]) { # LOOP THROUGH ARMS

r.disc[i,k] ~ dbin(p.disc[i,k],n.disc[i,k]) # binomial likelihood

logit(p.disc[i,k]) <- mu.disc[i,k]

mu.disc[i,k] ~ dnorm(mu.disc.mean[i,k], tau.m.cut)

mu.disc.mean[i,k] <- m.cut + delta.disc[i,k] + (x.disc[i,k]-x.base.mean)*beta.base.cut #+ x.disc[cov.index[2],i,k]*beta.base.cut[2] #+ x.disc[cov.index[3],i,k]*beta.base.cut[3] # model for linear predictor

delta.disc[i,k]<-d[t.disc[i,k]] - d[t.disc[i,1]] # model for linear predictor

rhat.disc[i,k] <- p.disc[i,k] * n.disc[i,k] # expected value of the numerators

dev.disc[i,k] <- 2 * (r.disc[i,k] * (log(r.disc[i,k])-log(rhat.disc[i,k]))

+ (n.disc[i,k]-r.disc[i,k]) * (log(n.disc[i,k]-r.disc[i,k]) - log(n.disc[i,k]-rhat.disc[i,k]))) #Deviance contribution

}

resdev.disc[i] <- sum(dev.disc[i,1:na.disc[i]]) # summed residual deviance contribution for this trial

}

# Calculate deviance ###################################

totresdev.disc<-sum(resdev.disc[]) # Total residual deviance for disconnected RCTs

totresdev.rct <- sum(resdev[]) # Total residual deviance for RCTs

totresdev<-totresdev.disc+totresdev.rct #Total Residual Deviance

# Priors for remaining parameters

d[1]<-0 # treatment effect is zero for reference treatment

for (k in 2:nt){ d[k] ~ dnorm(0,.01) } # vague priors for treatment effects

}

## reference.prediction.model.single.1cov.re.txt

# Reference prediction with random study effects to include single-arm studies

# Uses 1 covariate

# Have to use a model file in order to use the dnorm(,)I(,) syntax to truncate priors on sd in disconnected evidence

# Uses 'cut()' to prevent feedback from single-arm studies to baseline model

# Data are same as TSD format: ns, nt, na, r, n, t

# The data on baseline arms (from RCTs) are

# ns.base : number of baseline arms

# r.base : number of events in baseline arms

# n.base : number of patients in baseline arms

# The data on single arm studies are:

# ns.single : number of single arm studies

# r.single : number of events in single arm studies

# n.single : number of patients in single arm studies

# t.single : treatment in single arm study

# sd.connected.mean and sd.connected.tau are the mean and precision of the sd in the connected components.

# These are used as informative priors on sd.disc in the random effects models

# Binomial likelihood, logit link

# Simultaneous baseline and treat effects model for multi-arm trials

# Includes single-arm studies

model{ # *** PROGRAM STARTS

# Model for RCTs ##############################################

for(i in 1:ns){ # LOOP THROUGH STUDIES

w[i,1] <- 0 # adjustment for multi-arm trials is zero for control arm

delta[i,1] <- 0 # treatment effect is zero for control arm

mu[i] ~ dnorm(0,0.01) # Baseline are a nuisance for RCT evidence

for (k in 1:na[i]) { # LOOP THROUGH ARMS

r[i,k] ~ dbin(p[i,k],n[i,k]) # binomial likelihood

logit(p[i,k]) <- mu[i] + delta[i,k] # model for linear predictor

rhat[i,k] <- p[i,k] * n[i,k] # expected value of the numerators

dev.NA[i,k] <- 2 * (r[i,k] * (log(r[i,k])-log(rhat[i,k])) #Deviance contribution including NAs

+ (n[i,k]-r[i,k]) * (log(n[i,k]-r[i,k]) - log(n[i,k]-rhat[i,k])))

dev[i,k] <- dev.NA[i,k]*(1-equals(n[i,1],1)) # Deviance contribution with correction for NAs

}

resdev[i] <- sum(dev[i,1:na[i]]) # summed residual deviance contribution for this trial

for (k in 2:na[i]) { # LOOP THROUGH ARMS

delta[i,k] ~ dnorm(md[i,k],taud[i,k]) # trial-specific LOR distributions

md[i,k] <- d[t[i,k]] - d[t[i,1]] + sw[i,k] # mean of LOR distributions (with multi-arm trial correction)

taud[i,k] <- tau *2*(k-1)/k # precision of LOR distributions (with multi-arm trial correction)

w[i,k] <- (delta[i,k] - d[t[i,k]] + d[t[i,1]]) # adjustment for multi-arm RCTs

sw[i,k] <- sum(w[i,1:k-1])/(k-1) # cumulative adjustment for multi-arm trials

}

}

# Model for baseline effects ###################################

# Adapted from Program 1 of NICE DSU TSD 5

for (i in 1:ns.base){ # LOOP THROUGH STUDIES

r.base[i] ~ dbin(p.base[i],n.base[i]) # Likelihood

logit(p.base[i]) <- mu.base[i] # Log-odds of response

mu.base[i] ~ dnorm(mu.base.mean[i],tau.m) # Random effects model

mu.base.mean[i] <- m + (x.base[i]-x.base.mean)*beta.base # Prediction of mean effect

}

beta.base~dnorm(0,0.298) # vague prior for covariate effects

m ~ dnorm(0,.01) # vague prior for mean

var.m <- 1/tau.m # between-trial variance

tau.m <- pow(sd.m,-2) # between-trial precision = (1/between-trial variance)

sd.m ~ dunif(0,2) # vague prior for between-trial SD

# Model for single-arm studies #######################################

# Prevent feedback to baseline model (these two may not be necessary as mu.single is cut below

m.cut<-cut(m)

tau.m.cut<-cut(tau.m)

beta.base.cut<-cut(beta.base)

for(i in 1:ns.single)

{

r.single[i]~dbin(p.single[i],n.single[i])

logit(p.single[i]) <- mu.single[i]

mu.single[i] ~ dnorm(mu.single.mean[i], tau.m.cut)

mu.single.mean[i] <- m.cut + delta.single[i] + (x.single[i]-x.base.mean)*beta.base.cut

delta.single[i]~dnorm(d[t.single[i]],tau.disc) # Treatment effect relative to reference

rhat.single[i] <- p.single[i] * n.single[i] # expected value of the numerators

dev.single[i] <- 2 * (r.single[i] * (log(r.single[i])-log(rhat.single[i]))

+ (n.single[i]-r.single[i]) * (log(n.single[i]-r.single[i]) - log(n.single[i]-rhat.single[i]))) #Deviance contribution

}

# Calculate deviance ##############################################

totresdev.single<-sum(dev.single[]) # Total residual deviance for single-arm studies

totresdev.rct <- sum(resdev[]) # Total residual deviance for RCTs

totresdev<-totresdev.single+totresdev.rct #Total Residual Deviance

# Specify remaining priors

d[1]<-0 # treatment effect is zero for reference treatment

for (k in 2:nt){ d[k] ~ dnorm(0,.01) } # vague priors for treatment effects

sd ~ dunif(0,2) # vague prior for between-trial SD

tau <- pow(sd,-2) # between-trial precision = (1/between-trial variance)

# Priors for single-arm studies random study effects models

sd.disc ~ dnorm(sd.connected.mean, sd.connected.tau)I(0,) # Informative prior based on connected SD

tau.disc <- pow(sd.disc,-2) # between-trial precision = (1/between-trial variance)

}

## reference.prediction.model.disc.1cov.re.txt

# Reference prediction with random study effects to include disconnected RCTs

# Uses 1 covariate

# Have to use a model file in order to use the dnorm(,)I(,) syntax to truncate priors on sd in disconnected evidence

# Uses 'cut()' to prevent feedback from single-arm studies to baseline model

# Data are same as TSD format: ns, nt, na, r, n, t

# The data on baseline arms (from RCTs) are

# ns.base : number of baseline arms

# r.base : number of events in baseline arms

# n.base : number of patients in baseline arms

# The data on disconnected networks are (as in standard TSD):

# ns.disc, nt.disc, na.disc, r.disc, n.disc, t.disc

# sd.connected.mean and sd.connected.tau are the mean and precision of the sd in the connected components.

# These are used as informative priors on sd.disc in the random effects models

# Binomial likelihood, logit link

# Simultaneous baseline and treat effects model for multi-arm trials

# Includes disconnected RCTs

model{ # *** PROGRAM STARTS

# Model for RCTs ##############################################

for(i in 1:ns){ # LOOP THROUGH STUDIES

w[i,1] <- 0 # adjustment for multi-arm trials is zero for control arm

delta[i,1] <- 0 # treatment effect is zero for control arm

mu[i] ~ dnorm(0,0.01) # Baseline are a nuisance for RCT evidence

for (k in 1:na[i]) { # LOOP THROUGH ARMS

r[i,k] ~ dbin(p[i,k],n[i,k]) # binomial likelihood

logit(p[i,k]) <- mu[i] + delta[i,k] # model for linear predictor

rhat[i,k] <- p[i,k] * n[i,k] # expected value of the numerators

dev.NA[i,k] <- 2 * (r[i,k] * (log(r[i,k])-log(rhat[i,k])) #Deviance contribution including NAs

+ (n[i,k]-r[i,k]) * (log(n[i,k]-r[i,k]) - log(n[i,k]-rhat[i,k])))

dev[i,k] <- dev.NA[i,k]*(1-equals(n[i,1],1)) # Deviance contribution with correction for NAs

}

resdev[i] <- sum(dev[i,1:na[i]]) # summed residual deviance contribution for this trial

for (k in 2:na[i]) { # LOOP THROUGH ARMS

delta[i,k] ~ dnorm(md[i,k],taud[i,k]) # trial-specific LOR distributions

md[i,k] <- d[t[i,k]] - d[t[i,1]] + sw[i,k] # mean of LOR distributions (with multi-arm trial correction)

taud[i,k] <- tau *2*(k-1)/k # precision of LOR distributions (with multi-arm trial correction)

w[i,k] <- (delta[i,k] - d[t[i,k]] + d[t[i,1]]) # adjustment for multi-arm RCTs

sw[i,k] <- sum(w[i,1:k-1])/(k-1) # cumulative adjustment for multi-arm trials

}

}

# Model for baseline effects ###################################

# Adapted from Program 1 of NICE DSU TSD 5

for (i in 1:ns.base){ # LOOP THROUGH STUDIES

r.base[i] ~ dbin(p.base[i],n.base[i]) # Likelihood

logit(p.base[i]) <- mu.base[i] # Log-odds of response

mu.base[i] ~ dnorm(mu.base.mean[i],tau.m) # Random effects model

mu.base.mean[i] <- m + (x.base[i]-x.base.mean)*beta.base # Prediction of mean effect

}

beta.base~dnorm(0,0.298) # vague prior for covariate effects

m ~ dnorm(0,.01) # vague prior for mean

var.m <- 1/tau.m # between-trial variance

tau.m <- pow(sd.m,-2) # between-trial precision = (1/between-trial variance)

sd.m ~ dunif(0,5) # vague prior for between-trial SD

# Model for disconnected RCTs #######################################

# Prevent feedback to baseline model (these two may not be necessary as mu.disc is cut below

m.cut<-cut(m)

tau.m.cut<-cut(tau.m)

beta.base.cut<-cut(beta.base)

for(i in 1:ns.disc){ # LOOP THROUGH STUDIES

for (k in 1:na.disc[i]) { # LOOP THROUGH ARMS

r.disc[i,k] ~ dbin(p.disc[i,k],n.disc[i,k]) # binomial likelihood

logit(p.disc[i,k]) <- mu.disc[i,k]

mu.disc[i,k] ~ dnorm(mu.disc.mean[i,k], tau.m.cut)

mu.disc.mean[i,k] <- m.cut + delta.disc[i,k] + (x.disc[i,k]-x.base.mean)*beta.base.cut #+ x.disc[cov.index[2],i,k]*beta.base.cut[2] #+ x.disc[cov.index[3],i,k]*beta.base.cut[3] # model for linear predictor

rhat.disc[i,k] <- p.disc[i,k] * n.disc[i,k] # expected value of the numerators

dev.NA.disc[i,k] <- 2 * (r.disc[i,k] * (log(r.disc[i,k])-log(rhat.disc[i,k])) #Deviance contribution including NAs

+ (n.disc[i,k]-r.disc[i,k]) * (log(n.disc[i,k]-r.disc[i,k]) - log(n.disc[i,k]-rhat.disc[i,k])))

dev.disc[i,k] <- dev.NA.disc[i,k]*(1-equals(n.disc[i,1],1)) # Deviance contribution with correction for NAs

}

resdev.disc[i] <- sum(dev.disc[i,1:na.disc[i]]) # summed residual deviance contribution for this trial

delta.disc[i,1] ~ dnorm(d[t.disc[i,1]], tau.disc) # Handle k=1 separately as sum dissappears.

w.disc[i,1] <- 0 # adjustment for multi-arm trials is zero for control arm

for (k in 2:na.disc[i]) { # LOOP THROUGH ARMS (including control arm)

delta.disc[i,k] ~ dnorm(md.disc[i,k],taud.disc[i,k]) # trial-specific LOR distributions

md.disc[i,k] <- d[t.disc[i,k]] + sw.disc[i,k] # mean of LOR distributions (with multi-arm trial correction)

taud.disc[i,k] <- tau.disc *2*k/(k+1) # precision of LOR distributions (with multi-arm trial correction)

w.disc[i,k] <- (delta.disc[i,k] - d[t.disc[i,k]] ) # adjustment for multi-arm RCTs

sw.disc[i,k] <- sum(w.disc[i,1:(k-1)])/k # cumulative adjustment for multi-arm trials

}

}

# Calculate deviance ##############################################

totresdev.disc<-sum(resdev.disc[]) # Total residual deviance for disconnected RCTs

totresdev.rct <- sum(resdev[]) # Total residual deviance for RCTs

totresdev<-totresdev.disc+totresdev.rct #Total Residual Deviance

# Specify remaining priors

d[1]<-0 # treatment effect is zero for reference treatment

for (k in 2:nt){ d[k] ~ dnorm(0,.01) } # vague priors for treatment effects

sd ~ dunif(0,5) # vague prior for between-trial SD

tau <- pow(sd,-2) # between-trial precision = (1/between-trial variance)

# Priors for single-arm studies random study effects models

# Informative prior based on connected SD

sd.disc ~ dnorm(sd.connected.mean, sd.connected.tau)I(0,)

#sd.disc ~ dunif(sd.connected.LL, sd.connected.UL)

tau.disc <- pow(sd.disc,-2) # between-trial precision = (1/between-trial variance)

}

## alm.model.single.fe.R

# ALM model fixed study effects to include single-arm studies

# The closest matching RCT must be matched externally and the 'mu' taken from an independent baselines model

# Data are same as TSD format: ns, nt, na, r, n, t

# The data on single arm studies are:

# ns.single : number of single arm studies

# r.single : number of events in single arm studies

# n.single : number of patients in single arm studies

# t.single : treatment in single arm study

# mu.plugin[] is the plugin estimator from RCT with index matched.rct[]

# sd.connected.mean and sd.connected.tau are the mean and precision of the sd in the connected components.

# These are used as informative priors on sd.disc in the random effects models

model.alm.single.fe<-function()

{

# Model for RCTs ###################################

for(i in 1:ns){ # LOOP THROUGH STUDIES

mu[i] ~ dnorm(0,0.01) # random effect on baselines

for (k in 1:na[i]) { # LOOP THROUGH ARMS

r[i,k] ~ dbin(p[i,k],n[i,k]) # Binomial likelihood

logit(p[i,k]) <- mu[i] + delta[i,k]

delta[i,k]<-d[t[i,k]] - d[t[i,1]] # model for linear predictor

rhat[i,k] <- p[i,k] * n[i,k] # expected value of the numerators

dev[i,k] <- 2 * (r[i,k] * (log(r[i,k])-log(rhat[i,k]))

+ (n[i,k]-r[i,k]) * (log(n[i,k]-r[i,k]) - log(n[i,k]-rhat[i,k]))) #Deviance contribution

}

resdev[i] <- sum(dev[i,1:na[i]]) # summed residual deviance contribution for this trial

}

# Model for single-arm studies ###################################

for(i in 1:ns.single)

{

mu.plugin[i]~dnorm(mu.plugin.mean[i],mu.plugin.prec[i])

r.single[i]~dbin(p.single[i],n.single[i])

logit(p.single[i])<-mu.plugin[i]+delta.single[i]

delta.single[i]<-d[t.single[i]] - d[t[matched.rct[i],1]] # Treatment effect relative to reference

rhat.single[i] <- p.single[i] * n.single[i] # expected value of the numerators

dev.single[i] <- 2 * (r.single[i] * (log(r.single[i])-log(rhat.single[i]))

+ (n.single[i]-r.single[i]) * (log(n.single[i]-r.single[i]) - log(n.single[i]-rhat.single[i]))) #Deviance contribution

}

# Calculate deviance ###################################

totresdev.single<-sum(dev.single[]) # Total residual deviance for single-arm studies

totresdev.rct <- sum(resdev[]) # Total residual deviance for RCTs

totresdev<-totresdev.single+totresdev.rct #Total Residual Deviance

# Priors for remaining parameters

d[1]<-0 # treatment effect is zero for reference treatment

for (k in 2:nt){ d[k] ~ dnorm(0,.01) } # vague priors for treatment effects

}

## alm.model.disc.fe.R

# ALM model with fixed study effects to include disconnected RCTs

# The closest matching RCT must be matched externally and the 'mu' taken from an independent baselines model

# Extra data are mu.plugin[] and matched.rct[]. The latter is an indicator for the matched RCT

# Data are same as TSD format: ns, nt, na, r, n, t

# The data on disconnected networks are (as in standard TSD):

# ns.disc, nt.disc, na.disc, r.disc, n.disc, t.disc

# x.disc is matrix of covariates for disconnected RCTs.

# sd.connected.mean and sd.connected.tau are the mean and precision of the sd in the connected components.

# These are used as informative priors on sd.disc in the random effects models

model.alm.disc.fe<-function()

{

for(i in 1:ns){ # LOOP THROUGH STUDIES

mu[i] ~ dnorm(0,.01) # vague priors for all trial baselines

for (k in 1:na[i]) { # LOOP THROUGH ARMS

r[i,k] ~ dbin(p[i,k],n[i,k]) # Binomial likelihood

logit(p[i,k]) <- mu[i] + delta[i,k]

delta[i,k]<-d[t[i,k]] - d[t[i,1]] # model for linear predictor

rhat[i,k] <- p[i,k] * n[i,k] # expected value of the numerators

dev[i,k] <- 2 * (r[i,k] * (log(r[i,k])-log(rhat[i,k]))

+ (n[i,k]-r[i,k]) * (log(n[i,k]-r[i,k]) - log(n[i,k]-rhat[i,k]))) #Deviance contribution

}

resdev.rct[i] <- sum(dev[i,1:na[i]]) # summed residual deviance contribution for this trial

}

for(i in 1:ns.disc){ # LOOP THROUGH STUDIES

mu.plugin[i]~dnorm(mu.plugin.mean[i],mu.plugin.prec[i])

for (k in 1:na.disc[i]) { # LOOP THROUGH ARMS

r.disc[i,k] ~ dbin(p.disc[i,k],n.disc[i,k]) # Binomial likelihood

logit(p.disc[i,k]) <- mu.plugin[i] + delta.disc[i,k]

delta.disc[i,k]<-d[t.disc[i,k]] - d[t[matched.rct[i],1]] # model for linear predictor

rhat.disc[i,k] <- p.disc[i,k] * n.disc[i,k] # expected value of the numerators

dev.disc[i,k] <- 2 * (r.disc[i,k] * (log(r.disc[i,k])-log(rhat.disc[i,k]))

+ (n.disc[i,k]-r.disc[i,k]) * (log(n.disc[i,k]-r.disc[i,k]) - log(n.disc[i,k]-rhat.disc[i,k]))) #Deviance contribution

}

resdev.disc[i] <- sum(dev.disc[i,1:na.disc[i]]) # summed residual deviance contribution for this trial

}

totresdev.rct<-sum(resdev.rct[])

totresdev.disc<-sum(resdev.disc[])

totresdev <- totresdev.rct + totresdev.disc #Total Residual Deviance

d[1]<-0 # treatment effect is zero for reference treatment

for (k in 2:nt){ d[k] ~ dnorm(0,.01) } # vague priors for treatment effects

}

## alm.model.single.re.txt

# ALM with random study effects to include single-arm studies

# Have to use a model file in order to use the dnorm(,)I(,) syntax to truncate priors on sd in disconnected evidence

# The closest matching RCT must be matched externally and the 'mu' taken from an independent baselines model

# Data are same as TSD format: ns, nt, na, r, n, t

# The data on single arm studies are:

# ns.single : number of single arm studies

# r.single : number of events in single arm studies

# n.single : number of patients in single arm studies

# t.single : treatment in single arm study

# mu.plugin[] is the plugin estimator from RCT with index matched.rct[]

# sd.connected.mean and sd.connected.tau are the mean and precision of the sd in the connected components.

# These are used as informative priors on sd.disc in the random effects models

# Binomial likelihood, logit link

# Plugin estimator to include single-arm studies

model{ # *** PROGRAM STARTS

# Model for RCTs ##############################################

for(i in 1:ns){ # LOOP THROUGH STUDIES

w[i,1] <- 0 # adjustment for multi-arm trials is zero for control arm

delta[i,1] <- 0 # treatment effect is zero for control arm

mu[i] ~ dnorm(0,0.01) # Baseline are a nuisance for RCT evidence

for (k in 1:na[i]) { # LOOP THROUGH ARMS

r[i,k] ~ dbin(p[i,k],n[i,k]) # binomial likelihood

logit(p[i,k]) <- mu[i] + delta[i,k] # model for linear predictor

rhat[i,k] <- p[i,k] * n[i,k] # expected value of the numerators

dev.NA[i,k] <- 2 * (r[i,k] * (log(r[i,k])-log(rhat[i,k])) #Deviance contribution including NAs

+ (n[i,k]-r[i,k]) * (log(n[i,k]-r[i,k]) - log(n[i,k]-rhat[i,k])))

dev[i,k] <- dev.NA[i,k]*(1-equals(n[i,1],1)) # Deviance contribution with correction for NAs

}

resdev[i] <- sum(dev[i,1:na[i]]) # summed residual deviance contribution for this trial

for (k in 2:na[i]) { # LOOP THROUGH ARMS

delta[i,k] ~ dnorm(md[i,k],taud[i,k]) # trial-specific LOR distributions

md[i,k] <- d[t[i,k]] - d[t[i,1]] + sw[i,k] # mean of LOR distributions (with multi-arm trial correction)

taud[i,k] <- tau *2*(k-1)/k # precision of LOR distributions (with multi-arm trial correction)

w[i,k] <- (delta[i,k] - d[t[i,k]] + d[t[i,1]]) # adjustment for multi-arm RCTs

sw[i,k] <- sum(w[i,1:k-1])/(k-1) # cumulative adjustment for multi-arm trials

}

}

# Model for single-arm studies #######################################

for(i in 1:ns.single)

{

r.single[i]~dbin(p.single[i],n.single[i])

mu.plugin[i]~dnorm(mu.plugin.mean[i],mu.plugin.prec[i])

logit(p.single[i])<-mu.plugin[i]+delta.single[i]

delta.single[i]~dnorm(md.single[i],tau.disc) # Treatment effect relative to reference

md.single[i]<- d[t.single[i]] - d[t[matched.rct[i],1]]

rhat.single[i] <- p.single[i] * n.single[i] # expected value of the numerators

dev.single[i] <- 2 * (r.single[i] * (log(r.single[i])-log(rhat.single[i]))

+ (n.single[i]-r.single[i]) * (log(n.single[i]-r.single[i]) - log(n.single[i]-rhat.single[i]))) #Deviance contribution

}

# Calculate deviance ##############################################

totresdev.single<-sum(dev.single[]) # Total residual deviance for single-arm studies

totresdev.rct <- sum(resdev[]) # Total residual deviance for RCTs

totresdev<-totresdev.single+totresdev.rct #Total Residual Deviance

# Specify remaining priors

d[1]<-0 # treatment effect is zero for reference treatment

for (k in 2:nt){ d[k] ~ dnorm(0,.01) } # vague priors for treatment effects

sd ~ dunif(0,2) # vague prior for between-trial SD

tau <- pow(sd,-2) # between-trial precision = (1/between-trial variance)

# Priors for single-arm studies random study effects models

sd.disc ~ dnorm(sd.connected.mean, sd.connected.tau)I(0,) # Informative prior based on connected SD

tau.disc <- pow(sd.disc,-2) # between-trial precision = (1/between-trial variance)

}

## alm.model.disc.re.txt

# ALM with random study effects to include disconnected RCTs

# Have to use a model file in order to use the dnorm(,)I(,) syntax to truncate priors on sd in disconnected evidence

# The closest matching RCT must be matched externally and the 'mu' taken from an independent baselines model

# Extra data are mu.plugin[] and matched.rct[]. The latter is an indicator for the matched RCT

# Data are same as TSD format: ns, nt, na, r, n, t

# The data on disconnected networks are (as in standard TSD):

# ns.disc, nt.disc, na.disc, r.disc, n.disc, t.disc

# x.disc is matrix of covariates for disconnected RCTs.

# sd.connected.mean and sd.connected.tau are the mean and precision of the sd in the connected components.

# These are used as informative priors on sd.disc in the random effects models

# Binomial likelihood, logit link

# Simultaneous baseline and treat effects model for multi-arm trials

model{ # *** PROGRAM STARTS

# Model for connected RCTs is as in independent baselines model

for(i in 1:ns){ # LOOP THROUGH STUDIES

w[i,1] <- 0 # adjustment for multi-arm trials is zero for control arm

delta[i,1] <- 0 # treatment effect is zero for control arm

mu[i] ~ dnorm(0,0.01) # model for trial baselines re treatment 1

for (k in 1:na[i]) { # LOOP THROUGH ARMS

r[i,k] ~ dbin(p[i,k],n[i,k]) # binomial likelihood

logit(p[i,k]) <- mu[i] + delta[i,k] # model for linear predictor

rhat[i,k] <- p[i,k] * n[i,k] # expected value of the numerators

dev.NA[i,k] <- 2 * (r[i,k] * (log(r[i,k])-log(rhat[i,k])) #Deviance contribution including NAs

+ (n[i,k]-r[i,k]) * (log(n[i,k]-r[i,k]) - log(n[i,k]-rhat[i,k])))

dev[i,k] <- dev.NA[i,k]*(1-equals(n[i,1],1)) #Deviance contribution with correction for NAs

}

resdev.rct[i] <- sum(dev[i,1:na[i]]) # summed residual deviance contribution for this trial

for (k in 2:na[i]) { # LOOP THROUGH ARMS

delta[i,k] ~ dnorm(md[i,k],taud[i,k]) # trial-specific LOR distributions

md[i,k] <- d[t[i,k]] - d[t[i,1]] + sw[i,k] # mean of LOR distributions (with multi-arm trial correction)

taud[i,k] <- tau *2*(k-1)/k # precision of LOR distributions (with multi-arm trial correction)

w[i,k] <- (delta[i,k] - d[t[i,k]] + d[t[i,1]]) # adjustment for multi-arm RCTs

sw[i,k] <- sum(w[i,1:k-1])/(k-1) # cumulative adjustment for multi-arm trials

}

}

# Disconnected RCTs

for(i in 1:ns.disc){ # LOOP THROUGH STUDIES

w.disc[i,1] <- 0 # adjustment for multi-arm trials is zero for control arm

delta.disc[i,1] <- 0 # treatment effect is zero for control arm

mu.plugin[i]~dnorm(mu.plugin.mean[i],mu.plugin.prec[i])

for (k in 1:na.disc[i]) { # LOOP THROUGH ARMS

r.disc[i,k] ~ dbin(p.disc[i,k],n.disc[i,k]) # binomial likelihood

logit(p.disc[i,k]) <- mu.plugin[i] + delta.disc[i,k] # model for linear predictor

rhat.disc[i,k] <- p.disc[i,k] * n.disc[i,k] # expected value of the numerators

dev.disc.NA[i,k] <- 2 * (r.disc[i,k] * (log(r.disc[i,k])-log(rhat.disc[i,k])) #Deviance contribution including NAs

+ (n.disc[i,k]-r.disc[i,k]) * (log(n.disc[i,k]-r.disc[i,k]) - log(n.disc[i,k]-rhat.disc[i,k])))

dev.disc[i,k] <- dev.disc.NA[i,k]*(1-equals(n.disc[i,1],1)) #Deviance contribution with correction for NAs

}

resdev.disc[i] <- sum(dev.disc[i,1:na.disc[i]]) # summed residual deviance contribution for this trial

# Multi-arm correction for disconnected RCTs

# Treatment effects are relative to the baseline of the matched RCT

md.disc[i,1] <- d[t.disc[i,1]] - d[t[matched.rct[i],1]]

for (k in 2:na.disc[i]) { # LOOP THROUGH ARMS

delta.disc[i,k] ~ dnorm(md.disc[i,k],taud.disc[i,k]) # trial-specific LOR distributions

md.disc[i,k] <- d[t.disc[i,k]] - d[t[matched.rct[i],1]] + sw.disc[i,k] # mean of LOR distributions (with multi-arm trial correction)

taud.disc[i,k] <- tau.disc *2*k/(k+1) # precision of LOR distributions (with multi-arm trial correction)

w.disc[i,k] <- (delta.disc[i,k] - d[t.disc[i,k]] - d[t[matched.rct[i],1]]) # adjustment for multi-arm RCTs

sw.disc[i,k] <- sum(w.disc[i,1:k-1])/k # cumulative adjustment for multi-arm trials

}

} # End loop over disconnected RCTs

totresdev.disc<-sum(resdev.disc[])

totresdev.rct<-sum(resdev.rct[])

totresdev <- totresdev.rct + totresdev.disc # Total Residual Deviance

d[1]<-0 # treatment effect is zero for reference treatment

for (k in 2:nt){ d[k] ~ dnorm(0,.01) } # vague priors for treatment effects

sd ~ dunif(0,2) # vague prior for between-trial SD

tau <- pow(sd,-2) # between-trial precision = (1/between-trial variance)

# Priors for disconnected RCT

sd.disc ~ dnorm(sd.connected.mean, sd.connected.tau)I(0,) # Informative prior based on connected SD

tau.disc <- pow(sd.disc,-2) # between-trial precision = (1/between-trial variance)

}
