# Supplementary material for: Network Meta-analysis on Disconnected Evidence Networks When Only Aggregate Data Are Available: Modified Methods to Include Disconnected Trials and Single-Arm Studies while Minimizing Bias
Source: Med Decis Making. 2022 May 7;42(7):906–22. doi: 10.1177/0272989X221097081 (PMC9459361; doi:10.1177/0272989X221097081)
Supplement: sj-docx-2-mdm-10.1177_0272989X221097081 – Supplemental material for Network Meta-analysis on Disconnected Evidence Networks When Only Aggregate Data Are Available: Modified Methods to Include Disconnected Trials and Single-Arm Studies while Minimizing Bias [file sj-docx-2-mdm-10.1177_0272989X221097081.docx]

# Appendix

**Table of contents**

A.1 Corrections for multi-arm trials under random study effects

A.1.1 Correction for contrast-based independent baselines NMA on connected networks

A.1.2 Correction for reference prediction

A.1.3 Correction for aggregate level matching

A.2. Further details on the atrial fibrillation constructed data example

A.2.1. Data summaries

A.2.2. Results of model comparison in constructed example

A.2.3. Results tables for ischemic stroke in constructed data example

A.2.4. Results on clinically relevant bleeding in constructed data example

A.3. Further details of the simulation study

A.3.1. Coverage, bias, and sample size calculation

A.3.2. Further simulation study results for random study effects 100 patients per arm

A.3.3. Simulation study results for fixed study effects 100 patients per arm

A.3.4. Further simulation study results for random study effects 1000 patients per arm

A.3.5. Simulation study results for fixed study effects 1000 patients per arm

## A.1 Corrections for multi-arm trials under random study effects

### A.1.1 Correction for contrast-based independent baselines NMA on connected networks

Under random effects where $\delta_{ik}\sim Normal(d_{t_{i1}t_{ik}},\sigma^{2})$, trials with more than 2 arms have more than one $\delta_{ik}$ and they are correlated as they are both relative to the same baseline arm on treatment $t_{i1}$. This is modelled by assuming the between-arm variance to be $\frac{\sigma^{2}}{2}$ and assuming the relative effects are jointly normal:

$$\vec{\delta_{i}}=\left( \begin{matrix} \delta_{i2} \\ \vdots\\ \delta_{ia_{i}} \end{matrix} \right)\sim N_{a_{i}-1}\left[ \left( \begin{matrix} d_{t_{i1}t_{i2}} \\ \vdots\\ d_{t_{i1}t_{ia_{i}}} \end{matrix} \right),\left( \begin{matrix} \sigma^{2} & \frac{\sigma^{2}}{2}\ldots& \frac{\sigma^{2}}{2} \\ \frac{\sigma^{2}}{2} & \ddots& \frac{\begin{aligned} \vdots\\ \sigma^{2} \end{aligned}}{2} \\ \frac{\begin{aligned} \vdots\\ \sigma^{2} \end{aligned}}{2} & \cdots\frac{\sigma^{2}}{2} & \sigma^{2} \end{matrix} \right) \right]$$

where $a_{i}$is the number of arms in trial $i$. This can be written as the following univariate distribution

$$\delta_{ik}|\left( \begin{matrix} \delta_{i2} \\ \vdots\\ \delta_{i,\left( k-1 \right)} \end{matrix} \right)\sim N(d_{1t_{ik}}-d_{1t_{i1}}+\frac{1}{k-1}\sum_{j=1}^{k-1} \left[ \delta_{ij}-(d_{1t_{ij}}-d_{1t_{i1}}) \right],\frac{k}{2\left( k-1 \right)}\sigma^{2})$$

### A.1.2 Correction for reference prediction

A further issue with random effects on relates to the correction under random study effects models for correlation between arms in disconnected RCTs with greater than 2 arms. For the disconnected RCTs, this must be adapted as RCT specific treatment effects $\delta_{ik}$ are always relative to the reference $1$ and not a common baseline arm of the RCT.

The univariate distribution is now

$$\delta_{i1}\sim N\left( d_{1t_{i1}},{\sigma^{2}}^{'} \right)$$

Where $t_{i1}$ is the RCT specific baseline arm and we note that the disconnected RCT specific heterogeneity variance ${\sigma^{2}}^{'}$ is being used. For all $k>1$, we have

$$\delta_{ik}|\left( \begin{matrix} \delta_{i,1} \\ \vdots\\ \delta_{i,k-1} \end{matrix} \right)\sim N(d_{1t_{ik}}+\frac{1}{k}\sum_{j=1}^{k-1} \left[ \delta_{ij}-d_{1t_{ij}} \right],\frac{k+1}{2k}{\sigma^{2}}^{'})$$

### A.1.3 Correction for aggregate level matching

As the $\delta_{i^{'}k}$ are relative to $t_{i1}$ and not the baseline of $i^{'}$ the correlation correction for RCTs with 3 or more arms under random study effects must be modified. The correction is now

$$\delta_{i^{'}1}\sim N\left( d_{1t_{i^{'}1}}-d_{1t_{i1}},{\sigma^{2}}^{'} \right)$$

For all $k>1$, we have

$$\delta_{i^{'}k}|\left( \begin{matrix} \delta_{i^{'}1} \\ \vdots\\ \delta_{i^{'},k-1} \end{matrix} \right)\sim N(d_{1t_{i^{'}k}}-d_{1t_{i1}}+\frac{1}{k}\sum_{j=1}^{k-1} \left[ \delta_{i^{'}j}-d_{1t_{i^{'}j}} \right],\frac{k+1}{2k}{\sigma^{2}}^{'})$$

## A.2. Further details on the atrial fibrillation constructed data example

### A.2.1. Data summaries

For ischemic stroke, the number of events and patients on each arm of each trial are provided in Table 4 and the treatments on each arm in Table 5. For clinically relevant bleeding, the number of events and patients on each arm of each trial are provided in Table 6 and the treatments on each arm in Table 7. Note that continuity corrections of adding 0.5 to arms with zero events have been applied. The mean age, proportion male, and mean CHA_2_DS_2_-VASc score are provided in Table 8. These summaries are from the key NMA publications, where further details of the RCTs and systematic literature review methods can be found.(29, 36)

An illustration of disconnecting the clinically relevant bleeding network is provided in Figure 8.

Table 1 Ischemic stroke events/patients for each arm of each RCT in the connected NMA used for constructed data example. Dabigatran trials that were artificially disconnected are highlighted and at the bottom of the table.*

|  | **Arm 1** | **Arm 2** | **Arm 3** | **Arm 4** |
| --- | --- | --- | --- | --- |
| **ACTIVE W** | 42/3371 | 90/3335 | - | - |
| **AFASAK II** | 3/170 | 5/169 | - | - |
| **AF-VKA-ASA-CHINA** | 1/239 | 8/201 | - | - |
| **ARISTOTLE** | 175/9081 | 162/9120 | - | - |
| **ARISTOTLE-J** | 1/75 | 0.5/71 | 0.5/72 | - |
| **AVERROES** | 35/2808 | 93/2791 | - | - |
| **ENGAGE AF-TIMI 48** | 235/7036 | 333/7034 | 236/7035 | - |
| **EXPLORE-Xa** | 0.5/127 | 0.5/127 | 1/127 | 1/127 |
| **PATAF** | 3/131 | 4/141 | - | - |
| **ROCKET AF** | 161/7082 | 149/7061 | - | - |
| **SPAF II** | 13/358 | 13/197 | 19/357 | 18/188 |
| **RE-LY** | 134/6022 | 152/6015 | 103/6076 | - |

*Continuity correction of 0.5 added to arms with only zero events.

Table 2 Treatments on each arm of each RCT in ischemic stroke NMA used for the constructed data example. Dabigatran RCTs that were artificially disconnected are highlighted and at the bottom of the table.

|  | **Arm 1** | **Arm 2** | **Arm 3** | **Arm 4** |
| --- | --- | --- | --- | --- |
| **ACTIVE W** | Coumarin (INR 2-3) | Antiplatelet (>=150mg od) | - | - |
| **AFASAK II** | Coumarin (INR 2-3) | Antiplatelet (>=150mg od) | - | - |
| **AF-VKA-ASA-CHINA** | Coumarin (INR 2-3) | Antiplatelet (>=150mg od) | - | - |
| **ARISTOTLE** | Coumarin (INR 2-3) | Apixaban (5mg bd) | - | - |
| **ARISTOTLE-J** | Coumarin (INR 2-3) | Apixaban (5mg bd) | Apixaban (2.5mg bd) | - |
| **AVERROES** | Apixaban (5mg bd) | Antiplatelet (<150mg od) | - | - |
| **ENGAGE AF-TIMI 48** | Coumarin (INR 2-3) | Edoxaban (30mg od) | Edoxaban (60mg od) | - |
| **EXPLORE-Xa** | Coumarin (INR 2-3) | Betrixaban (40mg od) | Betrixaban (60mg od) | Betrixaban (80mg od) |
| **PATAF** | Coumarin (INR 2-3) | Antiplatelet (>=150mg od) | - | - |
| **ROCKET AF** | Coumarin (INR 2-3) | Rivaroxaban (20mg od) | - | - |
| **SPAF II** | Coumarin (INR 2-3) | Coumarin (INR 2-3) | Antiplatelet (>=150mg od) | Antiplatelet (>=150mg od) |
| **RE-LY** | Coumarin (INR 2-3) | Dabigatran (110mg bd) | Dabigatran (150mg bd) | - |

Table 3 Clinically relevant bleeding events/patients for each arm of each RCT in the connected NMA used for constructed data example. Dabigatran trials that were artificially disconnected are highlighted and at the bottom of the table.*

|  | **Arm 1** | **Arm 2** | **Arm 3** | **Arm 4** | **Arm 5** | **Arm 6** | **Arm 7** | **Arm 8** | **Arm 9** | **Arm 10** |
| --- | --- | --- | --- | --- | --- | --- | --- | --- | --- | --- |
| **ACTIVE W** | 93/3371 | 101/3335 | - | - | - | - | - | - | - | - |
| **AFASAK II** | 2/170 | 4/169 | - | - | - | - | - | - | - | - |
| **AF-EDOX-VKA-ASIA** | 5/75 | 0.5/79 | 6/80 | - | - | - | - | - | - | - |
| **AF-EDOX-VKA-JAPAN** | 2/130 | 7/134 | 7/130 | - | - | - | - | - | - | - |
| **AF-EDOX-VKA-MULTI** | 8/250 | 7/235 | 19/244 | 9/234 | 19/180 | - | - | - | - | - |
| **AF-VKA-ASA-CHINA** | 7/239 | 1/201 | - | - | - | - | - | - | - | - |
| **ARISTOTLE** | 340/9081 | 275/9120 | - | - | - | - | - | - | - | - |
| **ARISTOTLE-J** | 4/75 | 1/71 | 1/72 | - | - | - | - | - | - | - |
| **AVERROES** | 129/2808 | 110/2791 | - | - | - | - | - | - | - | - |
| **BAFTA** | 25/488 | 25/485 | - | - | - | - | - | - | - | - |
| **ENGAGE AF-TIMI 48** | 1788/7036 | 1182/7034 | 1571/7035 | - | - | - | - | - | - | - |
| **EXPLORE-Xa** | 9/127 | 1/127 | 5/127 | 5/127 | - | - | - | - | - | - |
| **ROCKET AF** | 1453/7082 | 1525/7061 | - | - | - | - | - | - | - | - |
| **WASPO** | 0.5/36 | 3/39 | - | - | - | - | - | - | - | - |
| **AF-DABIG-VKA-JAPAN** | 0.5/46 | 1/58 | - | - | - | - | - | - | - | - |
| **PETRO** | 0.5/70 | 0.5/100 | 0.5/33 | 0.5/36 | 0.5/105 | 3/30 | 1/34 | 0.5/59 | 0.5/27 | 0.5/21 |
| **RE-LY** | 334/6022 | 315/6015 | 363/6076 | - | - | - | - | - | - | - |

*Continuity correction of 0.5 added to arms with only zero events.

Table 4 Treatments on each arm of each RCT in clinically relevant bleeding NMA used for the constructed data example. Dabigatran RCTs that were artificially disconnected are highlighted and at the bottom of the table.

|  | **Arm 1** | **Arm 2** | **Arm 3** | **Arm 4** | **Arm 5** | **Arm 6** | **Arm 7** | **Arm 8** | **Arm 9** | **Arm 10** |
| --- | --- | --- | --- | --- | --- | --- | --- | --- | --- | --- |
| **ACTIVE W** | Coumarin (INR 2-3) | Antiplatelet (>=150mg od) | - | - | - | - | - | - | - | - |
| **AFASAK II** | Coumarin (INR 2-3) | Antiplatelet (>=150mg od) | - | - | - | - | - | - | - | - |
| **AF-EDOX-VKA-ASIA** | Coumarin (INR 2-3) | Edoxaban (30mg od) | Edoxaban (60mg od) | - | - | - | - | - | - | - |
| **AF-EDOX-VKA-JAPAN** | Edoxaban (30mg od) | Edoxaban (45mg od) | Edoxaban (60mg od) | - | - | - | - | - | - | - |
| **AF-EDOX-VKA-MULTI** | Coumarin (INR 2-3) | Edoxaban (30mg od) | Edoxaban (30mg bd) | Edoxaban (60mg od) | Edoxaban (60mg bd) | - | - | - | - | - |
| **AF-VKA-ASA-CHINA** | Coumarin (INR 2-3) | Antiplatelet (>=150mg od) | - | - | - | - | - | - | - | - |
| **ARISTOTLE** | Coumarin (INR 2-3) | Apixaban (5mg bd) | - | - | - | - | - | - | - | - |
| **ARISTOTLE-J** | Coumarin (INR 2-3) | Apixaban (5mg bd) | Apixaban (2.5mg bd) | - | - | - | - | - | - | - |
| **AVERROES** | Apixaban (5mg bd) | Antiplatelet (<150mg od) | - | - | - | - | - | - | - | - |
| **BAFTA** | Coumarin (INR 2-3) | Antiplatelet (<150mg od) | - | - | - | - | - | - | - | - |
| **ENGAGE AF-TIMI 48** | Coumarin (INR 2-3) | Edoxaban (30mg od) | Edoxaban (60mg od) | - | - | - | - | - | - | - |
| **EXPLORE-Xa** | Coumarin (INR 2-3) | Betrixaban (40mg od) | Betrixaban (60mg od) | Betrixaban (80mg od) | - | - | - | - | - | - |
| **ROCKET AF** | Coumarin (INR 2-3) | Rivaroxaban (20mg od) | - | - | - | - | - | - | - | - |
| **WASPO** | Coumarin (INR 2-3) | Antiplatelet (>=150mg od) | - | - | - | - | - | - | - | - |
| **AF-DABIG-VKA-JAPAN** | Dabigatran (110mg bd) | Dabigatran (150mg bd) | - | - | - | - | - | - | - | - |
| **PETRO** | Coumarin (INR 2-3) | Dabigatran (150mg bd) | Dabigatran (150mg bd) + Aspirin (325mg bd) | Dabigatran (150mg bd) + Aspirin (81mg bd) | Dabigatran (300mg bd) | Dabigatran (300mg bd) + Aspirin (325mg bd) | Dabigatran (300mg bd) + Aspirin (81mg bd) | Dabigatran (50mg bd) | Dabigatran (50mg bd) + Aspirin (325mg bd) | Dabigatran (50mg bd) + Aspirin (81mg bd) |
| **RE-LY** | Coumarin (INR 2-3) | Dabigatran (110mg bd) | Dabigatran (150mg bd) | - | - | - | - | - | - | - |

*INR = International normalised range

Table 5 Baseline age, percentage male, and mean CHA2DS2-VASc score for RCTs in the NMA used for the constructed data example. Dabigatran RCTs that were artificially disconnected are highlighted at the bottom of the table.*

| **Study** | **Intervention** | **Mean age (years)** | **Percentage male (%)** | **mean CHA_2_DS_2_-VASc score** |
| --- | --- | --- | --- | --- |
| **ACTIVE W** | Antiplatelet (Clopidogrel 75mg + (aspirin 75-100mg) od | 70.2 | 67 | 2 |
| **ACTIVE W** | Warfarin (INR 2-3, some patients may have received other vitamin K antagonists) | 70.2 | 66 | 2 |
| **AFASAK II** | Aspirin 300mg od | 73.1 | 65 | NR |
| **AFASAK II** | Warfarin (INR 2-3) | 73.2 | 57 | NR |
| **AF-DABIG-VKA-JAPAN** | Warfarin (INR 2-3, ≥1.6 to ≤2.6 in ≥70 yrs) | NR | NR | NR |
| **AF-EDOX-VKA-ASIA** | Edoxaban 30mg od | 64.9 | 64.6 | 2 |
| **AF-EDOX-VKA-ASIA** | Edoxaban 60mg od | 65.9 | 68.8 | 1.9 |
| **AF-EDOX-VKA-ASIA** | Warfarin (INR 2-3) | 64.5 | 62.7 | 1.8 |
| **AF-EDOX-VKA-JAPAN** | Edoxaban 30mg od | 69.4 | 84 | 1.9 |
| **AF-EDOX-VKA-JAPAN** | Edoxaban 45mg od | 69.5 | 81.3 | 2.1 |
| **AF-EDOX-VKA-JAPAN** | Edoxaban 60mg od | 68.4 | 81.7 | 2.1 |
| **AF-EDOX-VKA-JAPAN** | Warfarin (INR 2-3, 1.6-2.6 in ≥70 yrs.) | 68.8 | 82.9 | 2.2 |
|  |  |  |  |  |
| **AF-EDOX-VKA-MULTI** | Edoxaban 30mg od | 65.2 | 59.6 | 3.12 |
| **AF-EDOX-VKA-MULTI** | Edoxaban 30mg bd | 64.8 | 61.5 | 3.1 |
| **AF-EDOX-VKA-MULTI** | Edoxaban 60mg od | 64.9 | 66.2 | 3.06 |
| **AF-EDOX-VKA-MULTI** | Edoxaban 60mg bd | 64.7 | 63.3 | 3.06 |
| **AF-EDOX-VKA-MULTI** | Warfarin (INR 2-3) | 66 | 60.4 | 3.03 |
| **AF-VKA-ASA-CHINA** | Warfarin (INR 2.1-2.5) | 66.8 | 63.2 | NR |
| **AF-VKA-ASA-CHINA** | Aspirin 200mg od | 67.6 | 59.2 | NR |
| **ARISTOTLE** | Apixaban 5mg bd | 70 | 64.5 | 2.1 |
| **ARISTOTLE** | Warfarin (INR 2-3) | 70 | 65 | 2.1 |
| **ARISTOTLE-J** | Apixaban 2.5mg bd | 69.3 | 85.1 | 1.8 |
| **ARISTOTLE-J** | Apixaban 5mg bd | 70 | 82.4 | 2.1 |
| **ARISTOTLE-J** | Warfarin (INR 2-3, 2-2.6 in ≥70 yrs.) | 71.7 | 81.1 | 1.9 |
| **AVERROES** | Apixaban 5mg bd | 70 | 59 | 2 |
| **AVERROES** | Aspirin 81-324mg od | 70 | 58 | 2.1 |
| **BAFTA** | Aspirin 75mg od | 81.5 | 54 | 2.33 |
| **BAFTA** | Warfarin (INR 2-3) | 81.5 | 55 | 2.34 |
| **ENGAGE AF-TIMI 48** | Edoxaban 30mg od | 72 | 62.5 | 2.8 |
| **ENGAGE AF-TIMI 48** | Edoxaban 60mg od | 72 | 62.1 | 2.8 |
| **ENGAGE AF-TIMI 48** | Warfarin (INR 2-3) | 72 | 61.2 | 2.8 |
| **EXPLORE-Xa** | Betrixaban 40mg od | 73.3 | 62.2 | 2.59 |
| **EXPLORE-Xa** | Betrixaban 60mg od | 73.8 | 63.8 | 2.48 |
| **EXPLORE-Xa** | Betrixaban 80mg od | 72 | 70.1 | 2.06 |
| **EXPLORE-Xa** | Warfarin (INR 2-3) | 72.7 | 70.1 | 2.51 |
| **PATAF** | Aspirin150mg od | 70.8 | 48 | NR |
| **PATAF** | Dicoumarol (INR 2.5-3.5, some patients received other coumarins – phenprocoumon or acenocoumarol) | 70 | 44 | NR |
| **ROCKET AF** | Rivaroxaban 20mg od | 73 | 60.3 | 3.48 |
| **ROCKET AF** | Warfarin (INR 2-3) | 73 | 60.3 | 3.46 |
| **SPAF II** | Warfarin (INR 2-4.5 in <75yrs.) | 65 | 75 | NR |
| **SPAF II** | Aspirin 325mg (in <75 yrs.) | 64 | 76 | NR |
| **SPAF II** | Warfarin (INR 2-4.5 in ≥75yrs.) | 80 | 59 | NR |
| **SPAF II** | Aspirin 325mg (in ≥75 yrs.) | 80 | 58 | NR |
| **WASPO** | Warfarin (INR 2-3) | 83.5 | 39 | NR |
| **WASPO** | Aspirin 300mg od | 82.6 | 54 | NR |
| **AF-DABIG-VKA-JAPAN** | Dabigatran110mg bd | NR | NR | NR |
| **AF-DABIG-VKA-JAPAN** | Dabigatran 150mg bd | NR | NR | NR |
| **PETRO** | Dabigatran 50mg bd | 70 | 80 | NR |
| **PETRO** | Dabigatran 50mg + Aspirin 81mg bd | NR | NR | NR |
| **PETRO** | Dabigatran 50mg + Aspirin 325mg bd | NR | NR | NR |
| **PETRO** | Dabigatran 150mg bd | 70 | 81.3 | NR |
| **PETRO** | Dabigatran 150mg + Aspirin 81mg bd | NR | NR | NR |
| **PETRO** | Dabigatran 150mg + Aspirin 325mg bd | NR | NR | NR |
| **PETRO** | Dabigatran 300mg bd | 69.5 | 82.6 | NR |
| **PETRO** | Dabigatran 300mg + Aspirin 81mg bd | NR | NR | NR |
| **PETRO** | Dabigatran 300mg + Aspirin 325mg bd | NR | NR | NR |
| **PETRO** | Warfarin (INR 2-3) | 69 | 84.3 | NR |
| **RE-LY** | Dabigatran 110mg bd | 71.4 | 64.3 | 2.1 |
| **RE-LY** | Dabigatran 150mg bd | 71.5 | 63.2 | 2.2 |
| **RE-LY** | Warfarin (INR 2-3) | 71.6 | 63.3 | 2.1 |

*AF = atrial fibrillation; INR = international normalized ratio; NA; not applicable; NR = not reported, od = once daily; bd = twice daily, asp = aspirin

Figure 9 Disconnecting the clinically relevant bleeding atrial fibrillation network. 17 RCTs on 24 interventions are changed to either 3 disconnected RCTs and 14 connected RCTs, or 12 single-arm studies and 14 connected RCTs.


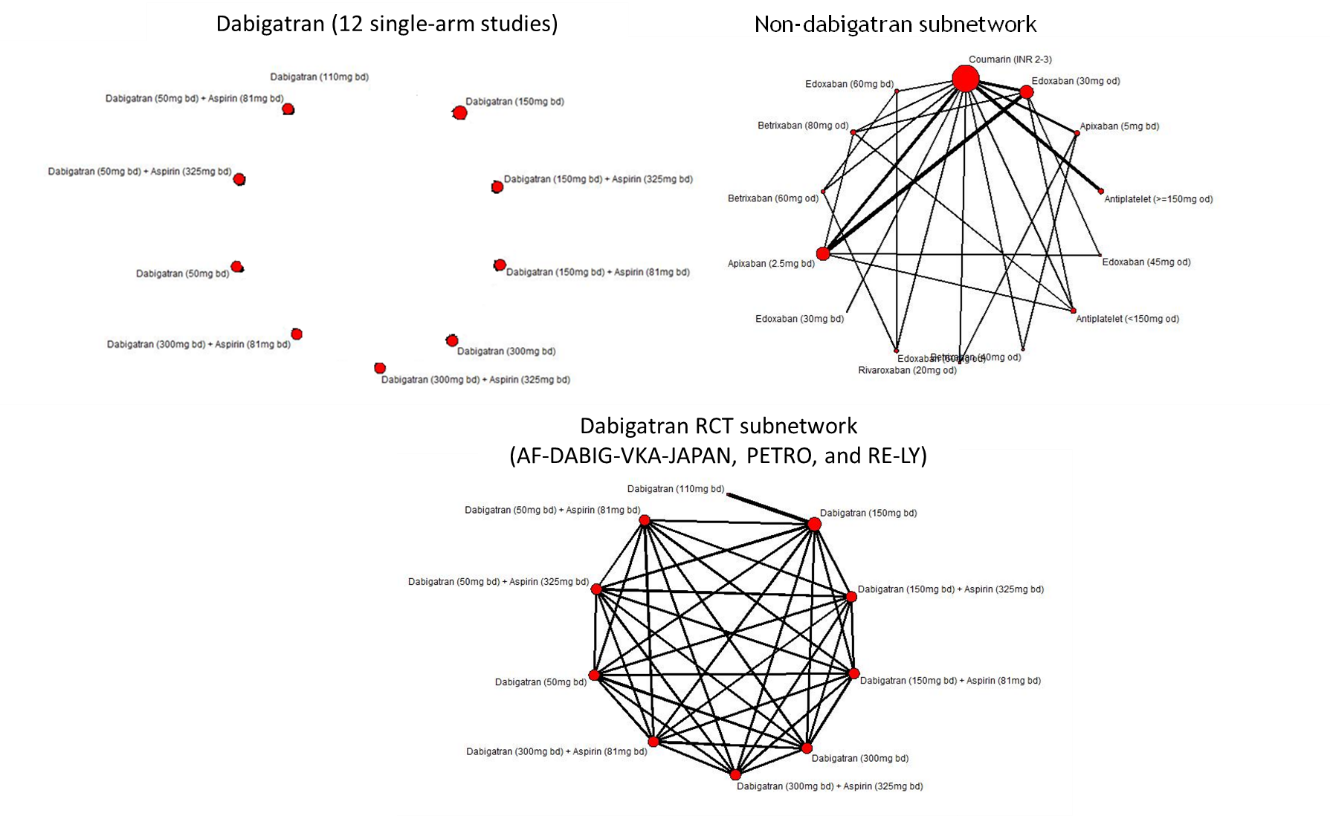


### A.2.2. Results of model comparison in constructed example

The possible models for reference prediction are compared in Table 9. All fixed effects models have substantially higher (worse) DIC and residual deviance that is substantially greater than the number of datapoints (i.e. number of studies with coumarin arms, n=11). Of the random effects models, adding covariates does not substantially reduce DIC or have meaningful impact on residual deviance. The random effects model with no covariates is therefore selected.

Table 6 Statistical assessment of reference prediction model on ischemic stroke (95% credible intervals). Lower values of deviance and DIC are preferred. Residual deviance close to number of datapoints (n=11) indicates good fit.

| **Covariates** | **Residual deviance** | **DIC** | **Residual deviance** | **DIC** |
| --- | --- | --- | --- | --- |
|  | **Fixed effects** | | **Random effects** | |
| No covariates | 79.3 (78.3, 83.3) | 127.1 | 11.9 (4.24, 23.1) | 67.14 |
| Age | 70.8 (62, 82.1) | 119 | 11.6 (4.3, 21.8) | 66.96 |
| Proportion male | 73.7 (71.8, 78.8) | 122.3 | 11.8 (4.16, 23.3) | 67.72 |
| CHADS2 | 68 (66, 73.4) | 116.7 | 11.6 (4.12, 22.7) | 67.03 |
| Age and proportion male | 68.3 (62.2, 76) | 117.3 | 11.9 (4.3, 22.9) | 67.67 |
| Age and CHADS2 | 70.3 (63.7, 77.6) | 119.2 | 11.8 (4.35, 22.6) | 67.3 |
| Proportion male and CHADS2 | 69.6 (66.3, 76.1) | 119 | 11.7 (4.1, 23.2) | 67.81 |
| All 3 covariates | 69.8 (63.5, 78.4) | 119.8 | 11.8 (4.25, 22.9) | 67.93 |

### A.2.3. Results tables for ischemic stroke in constructed data example

Table 7 Ischemic stroke under random study effects. Treatments in RCTs connected to the reference only. Comparison of estimated log odds ratios using true method (all RCTs), reference prediction, and aggregate level matching. Means (95% CrI).

|  | **True (all RCTs)** | **Reference prediction** | **ALM** |
| --- | --- | --- | --- |
| Antiplatelet (>=150mg od) | 0.69 (0.055, 1.34) | 0.69 (0.071, 1.35) | 0.69 (0.067, 1.35) |
| Antiplatelet (<150mg od) | 0.87 (-0.99, 2.56) | 0.87 (-0.96, 2.58) | 0.87 (-0.97, 2.55) |
| Edoxaban (30mg od) | 0.36 (-0.94, 1.64) | 0.36 (-0.92, 1.63) | 0.36 (-0.93, 1.61) |
| Betrixaban (40mg od) | -0.48 (-6.45, 4.69) | -0.41 (-6.26, 4.67) | -0.36 (-6.09, 4.75) |
| Rivaroxaban (20mg od) | -0.077 (-1.37, 1.23) | -0.075 (-1.36, 1.20) | -0.077 (-1.37, 1.22) |
| Apixaban (2.5mg bd) | -1.11 (-6.21, 2.67) | -1.22 (-6.61, 2.51) | -1.19 (-6.43, 2.60) |
| Edoxaban (60mg od) | 0.0018 (-1.28, 1.29) | 0.0028 (-1.29, 1.28) | 0.0058 (-1.27, 1.29) |
| Betrixaban (60mg od) | 0.87 (-3.08, 5.37) | 0.89 (-3.20, 5.49) | 0.91 (-3.19, 5.59) |
| Betrixaban (80mg od) | 0.79 (-3.33, 5.33) | 0.89 (-3.15, 5.51) | 0.91 (-3.19, 5.52) |

Table 8 Ischemic stroke under random study effects. Treatments in RCTs disconnected from the reference only. Comparison of estimated log odds ratios using true method (all RCTs), reference prediction, and aggregate level matching. Means (95% CrI).

|  | **True (all RCTs)** | **Reference prediction** | **ALM** |
| --- | --- | --- | --- |
| Dabigatran (110mg bd) | 0.13 (-1.14, 1.39) | 0.23 (-1.70, 2.24) | -0.0065 (-19.59, 19.68) |
| Dabigatran (150mg bd) | -0.28 (-1.55, 0.99) | -0.19 (-1.98, 1.71) | -0.21 (-1.36, 0.94) |

Table 9 Ischemic stroke under random study effects. Treatments in single-arm studies only. Comparison of estimated log odds ratios using true method (all RCTs), reference prediction, and aggregate level matching. Means (95% CrI).

|  | **True (all RCTs)** | **Reference prediction** | **ALM** |
| --- | --- | --- | --- |
| Dabigatran (110mg bd) | 0.13 (-1.14, 1.39) | 0.23 (-1.90, 2.42) | 1.19 (-1.03, 3.42) |
| Dabigatran (150mg bd) | -0.28 (-1.55, 0.99) | -0.18 (-2.29, 2.01) | -0.70 (-2.05, 0.67) |

Table 10 Ischemic stroke under fixed study effects. Treatments in RCTs connected to the reference only. Comparison of estimated log odds ratios using true method (all RCTs), reference prediction, and aggregate level matching. Means (95% CrI).

|  | **True (all RCTs)** | **Reference prediction** | **ALM** |
| --- | --- | --- | --- |
| Antiplatelet (>=150mg od) | 0.69 (0.41, 0.98) | 0.69 (0.41, 0.98) | 0.69 (0.41, 0.98) |
| Antiplatelet (<150mg od) | 0.93 (0.48, 1.38) | 0.93 (0.49, 1.39) | 0.92 (0.48, 1.38) |
| Edoxaban (30mg od) | 0.36 (0.19, 0.54) | 0.36 (0.19, 0.54) | 0.36 (0.19, 0.54) |
| Betrixaban (40mg od) | -0.42 (-6.20, 4.64) | 0.0034 (-6.48, 6.59) | -0.39 (-6.07, 4.64) |
| Rivaroxaban (20mg od) | -0.076 (-0.30, 0.15) | -0.077 (-0.30, 0.15) | -0.077 (-0.30, 0.15) |
| Apixaban (2.5mg bd) | -1.24 (-6.58, 2.34) | -1.33 (-7.23, 2.42) | -1.25 (-6.62, 2.34) |
| Edoxaban (60mg od) | 0.0046 (-0.18, 0.19) | 0.0047 (-0.18, 0.19) | 0.0047 (-0.18, 0.19) |
| Betrixaban (60mg od) | 0.90 (-3.02, 5.44) | 1.39 (-3.01, 7.50) | 0.91 (-3.02, 5.46) |
| Betrixaban (80mg od) | 0.89 (-3.03, 5.46) | 1.40 (-2.98, 7.47) | 0.90 (-3.05, 5.47) |

Table 11 Ischemic stroke under fixed study effects. Treatments in RCTs disconnected from the reference only. Comparison of estimated log odds ratios using true method (all RCTs), reference prediction, and aggregate level matching. Means (95% CrI).

|  | **True (all RCTs)** | **Reference prediction** | **ALM** |
| --- | --- | --- | --- |
| Dabigatran (110mg bd) | 0.13 (-0.11, 0.37) | 0.22 (-1.34, 1.91) | 0.66 (0.35, 0.96) |
| Dabigatran (150mg bd) | -0.28 (-0.54, -0.021) | -0.18 (-1.76, 1.50) | 0.25 (-0.075, 0.57) |

Table 12 Ischemic stroke under fixed study effects. Treatments in single-arm studies only. Comparison of estimated log odds ratios using true method (all RCTs), reference prediction, and aggregate level matching. Means (95% CrI).

|  | **True (all RCTs)** | **Reference prediction** | **ALM** |
| --- | --- | --- | --- |
| Dabigatran (110mg bd) | 0.13 (-0.11, 0.37) | 0.23 (-1.34, 1.91) | 1.20 (-0.66, 3.04) |
| Dabigatran (150mg bd) | -0.28 (-0.54, -0.021) | -0.18 (-1.76, 1.51) | -0.70 (-0.93, -0.47) |

### A.2.4. Results on clinically relevant bleeding in constructed data example

Clinically relevant bleeding results for methods with random study effects are presented in Figure 9, and those for fixed study effects in Figure 10.

Figure 10 Comparison of estimated log odds ratios using true method (all RCTs), reference prediction, and aggregate level matching under random study effects for the clinically relevant bleeding outcome. Point estimates are means and uncertainty intervals are 95% credible intervals.


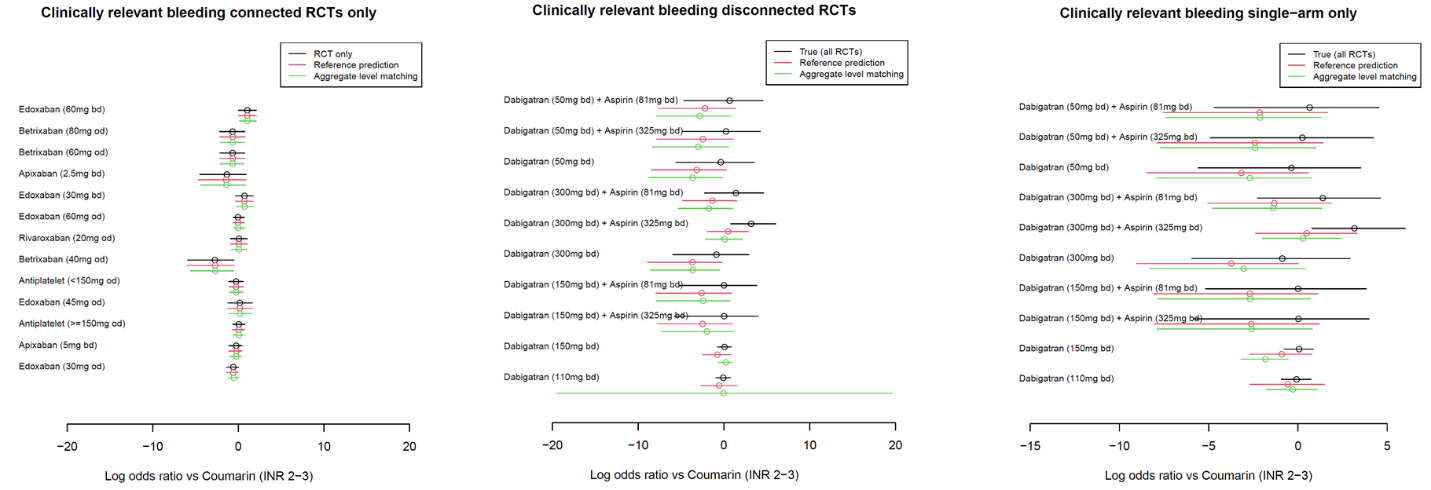


Figure 11 Comparison of estimated log odds ratios using true method (all RCTs), reference prediction, and aggregate level matching under fixed study effects for the clinically relevant bleeding outcome. Point estimates are means and uncertainty intervals are 95% credible intervals.


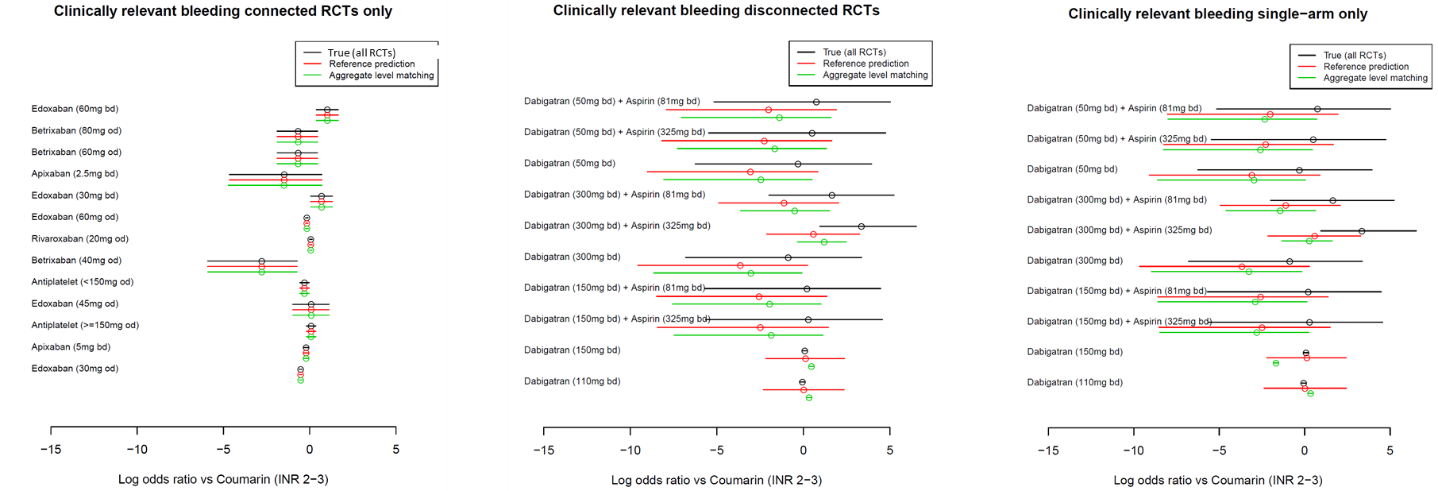


Table 6 Statistical assessment of reference prediction model on ischemic stroke (95% credible intervals). Lower values of deviance and DIC are preferred. Residual deviance close to number of datapoints (n=11) indicates good fit.

| **Covariates** | **Residual deviance** | **DIC** | **Residual deviance** | **DIC** |
| --- | --- | --- | --- | --- |
|  | **Fixed effects** | | **Random effects** | |
| No covariates | 79.3 (78.3, 83.3) | 127.1 | 11.9 (4.24, 23.1) | 67.14 |
| Age | 70.8 (62, 82.1) | 119 | 11.6 (4.3, 21.8) | 66.96 |
| Proportion male | 73.7 (71.8, 78.8) | 122.3 | 11.8 (4.16, 23.3) | 67.72 |
| CHADS2 | 68 (66, 73.4) | 116.7 | 11.6 (4.12, 22.7) | 67.03 |
| Age and proportion male | 68.3 (62.2, 76) | 117.3 | 11.9 (4.3, 22.9) | 67.67 |
| Age and CHADS2 | 70.3 (63.7, 77.6) | 119.2 | 11.8 (4.35, 22.6) | 67.3 |
| Proportion male and CHADS2 | 69.6 (66.3, 76.1) | 119 | 11.7 (4.1, 23.2) | 67.81 |
| All 3 covariates | 69.8 (63.5, 78.4) | 119.8 | 11.8 (4.25, 22.9) | 67.93 |

### A.2.3. Results tables for ischemic stroke in constructed data example

Table 7 Clinically relevant bleeding under random study effects. Treatments in RCTs connected to the reference only. Comparison of estimated log odds ratios using true method (all RCTs), reference prediction, and aggregate level matching. Means (95% CrI).

|  | **True (all RCTs)** | **Reference prediction** | **ALM** |
| --- | --- | --- | --- |
| Edoxaban (30mg od) | -0.59 (-1.37, 0.009) | -0.60 (-1.40, 0.0086) | -0.54 (-1.18, 0.065) |
| Apixaban (5mg bd) | -0.25 (-1.10, 0.42) | -0.24 (-1.11, 0.42) | -0.23 (-1.02, 0.39) |
| Antiplatelet (>=150mg od) | 0.055 (-0.65, 0.75) | 0.053 (-0.68, 0.75) | 0.054 (-0.61, 0.71) |
| Edoxaban (45mg od) | 0.17 (-1.22, 1.63) | 0.16 (-1.24, 1.63) | 0.18 (-1.13, 1.61) |
| Antiplatelet (<150mg od) | -0.26 (-1.11, 0.58) | -0.26 (-1.11, 0.58) | -0.26 (-1.04, 0.53) |
| Betrixaban (40mg od) | -2.75 (-5.90, -0.52) | -2.72 (-5.98, -0.49) | -2.70 (-5.61, -0.54) |
| Rivaroxaban (20mg od) | 0.065 (-0.91, 1.04) | 0.066 (-0.92, 1.06) | 0.069 (-0.83, 0.98) |
| Edoxaban (60mg od) | -0.04 (-0.60, 0.71) | -0.041 (-0.63, 0.73) | -0.023 (-0.52, 0.74) |
| Edoxaban (30mg bd) | 0.71 (-0.32, 1.75) | 0.71 (-0.34, 1.76) | 0.74 (-0.23, 1.75) |
| Apixaban (2.5mg bd) | -1.34 (-4.50, 0.92) | -1.44 (-4.67, 0.87) | -1.39 (-4.41, 0.83) |
| Betrixaban (60mg od) | -0.66 (-2.14, 0.75) | -0.66 (-2.17, 0.78) | -0.68 (-2.15, 0.70) |
| Betrixaban (80mg od) | -0.67 (-2.18, 0.75) | -0.66 (-2.16, 0.79) | -0.66 (-2.12, 0.74) |
| Edoxaban (60mg bd) | 1.05 (0.015, 2.10) | 1.05 (-0.0035, 2.10) | 1.07 (0.12, 2.10) |

Table 8 Clinically relevant bleeding under random study effects. Treatments in RCTs disconnected from the reference only. Comparison of estimated log odds ratios using true method (all RCTs), reference prediction, and aggregate level matching. Means (95% CrI).

|  | **True (all RCTs)** | **Reference prediction** | **ALM** |
| --- | --- | --- | --- |
| Dabigatran (110mg bd) | -0.076 (-0.94, 0.74) | -0.56 (-2.68, 1.50) | -0.047 (-19.53, 19.61) |
| Dabigatran (150mg bd) | 0.065 (-0.76, 0.86) | -0.77 (-2.47, 0.83) | 0.22 (-0.64, 0.89) |
| Dabigatran (150mg bd) + Aspirin (325mg bd) | 0.024 (-5.81, 3.98) | -2.49 (-7.74, 0.99) | -1.98 (-7.33, 1.13) |
| Dabigatran (150mg bd) + Aspirin (81mg bd) | 0.0032 (-5.18, 3.83) | -2.61 (-7.92, 0.89) | -2.44 (-7.90, 0.75) |
| Dabigatran (300mg bd) | -0.88 (-5.95, 2.92) | -3.68 (-8.89, -0.22) | -3.62 (-8.57, -0.49) |
| Dabigatran (300mg bd) + Aspirin (325mg bd) | 3.17 (0.79, 6.01) | 0.48 (-1.92, 2.85) | 0.095 (-2.13, 2.18) |
| Dabigatran (300mg bd) + Aspirin (81mg bd) | 1.39 (-2.27, 4.63) | -1.34 (-4.84, 1.48) | -1.76 (-5.30, 0.99) |
| Dabigatran (50mg bd) | -0.36 (-5.60, 3.53) | -3.19 (-8.47, 0.30) | -3.64 (-8.72, -0.14) |
| Dabigatran (50mg bd) + Aspirin (325mg bd) | 0.25 (-4.91, 4.23) | -2.45 (-7.82, 1.07) | -3.00 (-8.32, 0.56) |
| Dabigatran (50mg bd) + Aspirin (81mg bd) | 0.65 (-4.69, 4.55) | -2.21 (-7.56, 1.33) | -2.84 (-7.87, 0.78) |

Table 9 Clinically relevant bleeding under random study effects. Treatments in single-arm studies only. Comparison of estimated log odds ratios using true method (all RCTs), reference prediction, and aggregate level matching. Means (95% CrI).

|  | **True (all RCTs)** | **Reference prediction** | **ALM** |
| --- | --- | --- | --- |
| Dabigatran (110mg bd) | -0.076 (-0.94, 0.74) | -0.57 (-2.69, 1.48) | -0.28 (-1.77, 1.07) |
| Dabigatran (150mg bd) | 0.065 (-0.76, 0.86) | -0.91 (-2.68, 0.77) | -1.82 (-3.19, -0.55) |
| Dabigatran (150mg bd) + Aspirin (325mg bd) | 0.024 (-5.81, 3.98) | -2.61 (-8.05, 1.20) | -2.59 (-7.88, 0.80) |
| Dabigatran (150mg bd) + Aspirin (81mg bd) | 0.0032 (-5.18, 3.83) | -2.70 (-8.07, 1.11) | -2.68 (-7.85, 0.71) |
| Dabigatran (300mg bd) | -0.88 (-5.95, 2.92) | -3.74 (-9.04, 0.024) | -3.05 (-8.32, 0.41) |
| Dabigatran (300mg bd) + Aspirin (325mg bd) | 3.17 (0.79, 6.01) | 0.48 (-2.37, 3.31) | 0.27 (-1.98, 2.44) |
| Dabigatran (300mg bd) + Aspirin (81mg bd) | 1.39 (-2.27, 4.63) | -1.32 (-5.05, 1.87) | -1.38 (-4.78, 1.34) |
| Dabigatran (50mg bd) | -0.36 (-5.60, 3.53) | -3.17 (-8.46, 0.60) | -2.70 (-7.94, 0.74) |
| Dabigatran (50mg bd) + Aspirin (325mg bd) | 0.25 (-4.91, 4.23) | -2.41 (-7.91, 1.40) | -2.41 (-7.70, 1.01) |
| Dabigatran (50mg bd) + Aspirin (81mg bd) | 0.65 (-4.69, 4.55) | -2.15 (-7.55, 1.66) | -2.13 (-7.40, 1.30) |

Table 10 Clinically relevant bleeding under fixed study effects. Treatments in RCTs connected to the reference only. Comparison of estimated log odds ratios using true method (all RCTs), reference prediction, and aggregate level matching. Means (95% CrI).

|  | **True (all RCTs)** | **Reference prediction** | **ALM** |
| --- | --- | --- | --- |
| Edoxaban (30mg od) | -0.52 (-0.61, -0.44) | -0.52 (-0.61, -0.44) | -0.52 (-0.60, -0.44) |
| Apixaban (5mg bd) | -0.21 (-0.36, -0.052) | -0.21 (-0.36, -0.052) | -0.21 (-0.36, -0.052) |
| Antiplatelet (>=150mg od) | 0.079 (-0.20, 0.35) | 0.079 (-0.20, 0.35) | 0.078 (-0.20, 0.35) |
| Edoxaban (45mg od) | 0.081 (-0.99, 1.11) | 0.085 (-0.98, 1.12) | 0.086 (-0.98, 1.12) |
| Antiplatelet (<150mg od) | -0.30 (-0.58, -0.035) | -0.30 (-0.57, -0.033) | -0.30 (-0.57, -0.035) |
| Betrixaban (40mg od) | -2.73 (-5.73, -0.72) | -2.78 (-5.92, -0.72) | -2.73 (-5.76, -0.72) |
| Rivaroxaban (20mg od) | 0.065 (-0.016, 0.15) | 0.065 (-0.016, 0.15) | 0.065 (-0.016, 0.15) |
| Edoxaban (60mg od) | -0.16 (-0.24, -0.088) | -0.16 (-0.24, -0.088) | -0.16 (-0.24, -0.085) |
| Edoxaban (30mg bd) | 0.68 (0.049, 1.31) | 0.69 (0.052, 1.30) | 0.69 (0.052, 1.31) |
| Apixaban (2.5mg bd) | -1.45 (-4.54, 0.70) | -1.48 (-4.66, 0.71) | -1.46 (-4.56, 0.70) |
| Betrixaban (60mg od) | -0.66 (-1.87, 0.46) | -0.67 (-1.89, 0.45) | -0.67 (-1.88, 0.46) |
| Betrixaban (80mg od) | -0.66 (-1.87, 0.46) | -0.67 (-1.89, 0.46) | -0.67 (-1.88, 0.46) |
| Edoxaban (60mg bd) | 1.02 (0.38, 1.65) | 1.02 (0.38, 1.66) | 1.02 (0.38, 1.65) |

Table 11 Clinically relevant bleeding under fixed study effects. Treatments in RCTs disconnected from the reference only. Comparison of estimated log odds ratios using true method (all RCTs), reference prediction, and aggregate level matching. Means (95% CrI).

|  | **True (all RCTs)** | **Reference prediction** | **ALM** |
| --- | --- | --- | --- |
| Dabigatran (110mg bd) | -0.062 (-0.22, 0.097) | 0.025 (-2.36, 2.44) | 0.33 (0.18, 0.49) |
| Dabigatran (150mg bd) | 0.078 (-0.075, 0.23) | 0.13 (-2.22, 2.45) | 0.46 (0.31, 0.61) |
| Dabigatran (150mg bd) + Aspirin (325mg bd) | 0.18 (-5.26, 4.05) | -2.48 (-8.45, 1.49) | -1.72 (-6.83, 1.12) |
| Dabigatran (150mg bd) + Aspirin (81mg bd) | 0.096 (-5.38, 3.96) | -2.56 (-8.53, 1.39) | -1.80 (-6.92, 1.05) |
| Dabigatran (300mg bd) | -0.95 (-6.31, 2.85) | -3.65 (-9.61, 0.27) | -2.86 (-7.91, -0.058) |
| Dabigatran (300mg bd) + Aspirin (325mg bd) | 3.17 (0.91, 5.94) | 0.58 (-2.18, 3.31) | 1.20 (-0.34, 2.48) |
| Dabigatran (300mg bd) + Aspirin (81mg bd) | 1.46 (-2.06, 4.68) | -1.12 (-4.93, 2.09) | -0.48 (-3.55, 1.52) |
| Dabigatran (50mg bd) | -0.39 (-5.79, 3.46) | -3.06 (-8.97, 0.87) | -2.29 (-7.37, 0.53) |
| Dabigatran (50mg bd) + Aspirin (325mg bd) | 0.38 (-5.07, 4.26) | -2.26 (-8.16, 1.71) | -1.53 (-6.69, 1.35) |
| Dabigatran (50mg bd) + Aspirin (81mg bd) | 0.62 (-4.91, 4.51) | -1.99 (-7.93, 1.99) | -1.27 (-6.44, 1.62) |

Table 12 Clinically relevant bleeding under fixed study effects. Treatments in single-arm studies only. Comparison of estimated log odds ratios using true method (all RCTs), reference prediction, and aggregate level matching. Means (95% CrI).

|  | **True (all RCTs)** | **Reference prediction** | **ALM** |
| --- | --- | --- | --- |
| Dabigatran (110mg bd) | -0.062 (-0.22, 0.097) | 0.018 (-2.37, 2.43) | 0.34 (0.19, 0.50) |
| Dabigatran (150mg bd) | 0.078 (-0.075, 0.23) | 0.12 (-2.21, 2.43) | -1.68 (-1.80, -1.56) |
| Dabigatran (150mg bd) + Aspirin (325mg bd) | 0.18 (-5.26, 4.05) | -2.50 (-8.53, 1.49) | -2.60 (-7.65, 0.26) |
| Dabigatran (150mg bd) + Aspirin (81mg bd) | 0.096 (-5.38, 3.96) | -2.58 (-8.60, 1.37) | -2.70 (-7.77, 0.16) |
| Dabigatran (300mg bd) | -0.95 (-6.31, 2.85) | -3.67 (-9.67, 0.28) | -3.05 (-8.11, -0.14) |
| Dabigatran (300mg bd) + Aspirin (325mg bd) | 3.17 (0.91, 5.94) | 0.58 (-2.15, 3.28) | 0.28 (-1.31, 1.62) |
| Dabigatran (300mg bd) + Aspirin (81mg bd) | 1.46 (-2.06, 4.68) | -1.11 (-4.94, 2.08) | -1.40 (-4.49, 0.64) |
| Dabigatran (50mg bd) | -0.39 (-5.79, 3.46) | -3.09 (-9.09, 0.90) | -2.77 (-7.82, 0.072) |
| Dabigatran (50mg bd) + Aspirin (325mg bd) | 0.38 (-5.07, 4.26) | -2.28 (-8.24, 1.68) | -2.42 (-7.50, 0.47) |
| Dabigatran (50mg bd) + Aspirin (81mg bd) | 0.62 (-4.91, 4.51) | -2.02 (-8.03, 1.95) | -2.16 (-7.27, 0.75) |

## A.3. Further details of the simulation study

### A.3.1. Coverage, bias, and sample size calculation

Our sample size calculation for the simulation study follows the recommendations of Morris 2019 and is based on the coverage probability and bias of our methods.(33) If the 95% credible interval (CrI) of the method includes the ‘true’ $d_{1k}$ it is success, otherwise fail, and the proportion of simulations with success is the coverage probability.

The number of simulations based on expected coverage $E\left( Coverage \right)$ and desired Monte Carlo standard error (MSE) is

$$n_{sim}=\frac{E\left( Coverage \right)\times\left( 1-E\left( Coverage \right) \right)}{\left( MSE \right)^{2}}$$

Initial runs of the simulation using $n_{sim}=100$ estimates $E\left( Coverage \right)$ to be at least, or worst case, 0.35 and this implies a Monte Carlo SE of 0.047.

The bias is defined as

$$\frac{1}{n_{sim}}\sum_{i=1}^{n_{sim}} \hat{d}_{i}-d$$

The number of simulations required for a specific expected bias $E\left( bias \right)$ is

$$n_{sim}=\frac{{E\left( bias \right)}^{2}}{{MSE}^{2}}+1$$

Initial runs with $n_{sim}=100$ suggested the largest, or worst case, value of $E\left( bias \right)$ to be at worst 0.15 and this implies MSE of 0.015. From these we conclude that $n_{sim}=100$ is sufficient for our purposes.

### A.3.2. Further simulation study results for random study effects 100 patients per arm

Table 13 Estimated bias (95% credible interval) of simulation study using 100 simulations for random study effects models.*

|  |  | **5 RCTs** | | **15 RCTs** | | **50 RCTs** | |
| --- | --- | --- | --- | --- | --- | --- | --- |
|  |  | $\boldsymbol{\beta weak}$ | $\boldsymbol{\beta strong}$ | $\boldsymbol{\beta weak}$ | $\boldsymbol{\beta strong}$ | $\boldsymbol{\beta weak}$ | $\boldsymbol{\beta strong}$ |
| **Bias on connected** $\boldsymbol{\gamma=0}$ | **RCT only** | 0.016 (-0.0210, 0.053) | 0.025 (-0.084, 0.13) | -0.0089 (-0.033, 0.0148) | 0.0064 (-0.017, 0.030) | -0.0042 (-0.016, 0.0072) | -0.00056 (-0.0125, 0.011) |
|  | **ALM single** | 0.017 (-0.0210, 0.055) | 0.032 (-0.081, 0.15) | -0.0045 (-0.028, 0.0194) | 0.0072 (-0.017, 0.031) | -0.0047 (-0.016, 0.0069) | -0.00112 (-0.0132, 0.011) |
|  | **ALM disconnected** | 0.017 (-0.0210, 0.055) | 0.021 (-0.094, 0.14) | -0.0158 (-0.039, 0.0074) | 0.0082 (-0.016, 0.032) | -0.0039 (-0.015, 0.0076) | 0.00041 (-0.0113, 0.012) |
|  | **RP single** | 0.012 (-0.0233, 0.047) | 0.040 (-0.070, 0.15) | -0.0107 (-0.034, 0.0132) | 0.0047 (-0.019, 0.028) | -0.0039 (-0.015, 0.0074) | 0.00050 (-0.0113, 0.012) |
|  | **RP disconnected** | 0.032 (-0.0055, 0.070) | 0.034 (-0.086, 0.15) | -0.0101 (-0.034, 0.0141) | 0.0057 (-0.018, 0.030) | -0.0025 (-0.014, 0.0088) | 0.00213 (-0.0097, 0.014) |
| **Bias on disconnected** $\boldsymbol{\gamma=0}$ | **ALM single** | 0.36 ( 0.144, 0.58) | 0.225 ( 0.0015, 0.45) | 0.020 (-0.1427, 0.182) | 0.27 ( 0.080, 0.45) | 0.105 (-0.019, 0.23) | 0.392 ( 0.220, 0.56) |
|  | **ALM disconnected** | 0.12 (-0.031, 0.27) | -0.016 (-0.1697, 0.14) | -0.063 (-0.2174, 0.092) | 0.28 ( 0.090, 0.46) | 0.045 (-0.119, 0.21) | 0.096 (-0.045, 0.24) |
|  | **RP single** | 0.40 ( 0.206, 0.59) | 0.286 ( 0.1010, 0.47) | 0.121 (-0.0021, 0.243) | 0.24 ( 0.086, 0.39) | 0.207 ( 0.089, 0.32) | 0.310 ( 0.184, 0.44) |
|  | **RP disconnected** | 0.36 ( 0.154, 0.56) | 0.193 (-0.0455, 0.43) | 0.181 (-0.0079, 0.369) | 0.24 (-0.040, 0.52) | 0.242 ( 0.084, 0.40) | 0.113 (-0.064, 0.29) |
| **Bias on connected** $\boldsymbol{\gamma}\boldsymbol{\neq0}$ | **RCT only** | 0.019 (-0.0178, 0.056) | 0.030 (-0.068, 0.129) | 0.0038 (-0.014, 0.022) | -0.0110 (-0.031, 0.0093) | 0.00127 (-0.0080, 0.0106) | -0.00022 (-0.012, 0.012) |
|  | **ALM single** | 0.015 (-0.0224, 0.053) | 0.050 (-0.063, 0.163) | 0.0014 (-0.016, 0.019) | -0.0111 (-0.031, 0.0093) | 0.00311 (-0.0060, 0.0123) | 0.00012 (-0.012, 0.012) |
|  | **ALM disconnected** | 0.033 (-0.0037, 0.069) | 0.050 (-0.069, 0.168) | 0.0017 (-0.016, 0.019) | -0.0080 (-0.029, 0.0125) | 0.00349 (-0.0059, 0.0129) | 0.00085 (-0.011, 0.013) |
|  | **RP single** | 0.021 (-0.0167, 0.059) | 0.011 (-0.063, 0.085) | 0.0044 (-0.013, 0.022) | -0.0136 (-0.033, 0.0063) | 0.00041 (-0.0090, 0.0098) | -0.00088 (-0.013, 0.011) |
|  | **RP disconnected** | 0.031 (-0.0129, 0.075) | 0.047 (-0.070, 0.163) | 0.0066 (-0.012, 0.026) | -0.0083 (-0.029, 0.0125) | 0.00202 (-0.0073, 0.0114) | 0.00144 (-0.010, 0.013) |
| **Bias on disconnected** $\boldsymbol{\gamma}\boldsymbol{\neq0}$ | **ALM single** | -0.0692 (-0.23, 0.089) | -0.160 (-0.39, 0.072) | 0.046 (-0.074, 0.17) | 0.0326 (-0.135, 0.200) | -0.0184 (-0.131, 0.095) | 0.055 (-0.0578, 0.168) |
|  | **ALM disconnected** | -0.0321 (-0.20, 0.135) | -0.066 (-0.23, 0.097) | -0.036 (-0.181, 0.11) | -0.0057 (-0.149, 0.137) | 0.0177 (-0.136, 0.171) | 0.225 ( 0.0719, 0.379) |
|  | **RP single** | -0.0382 (-0.17, 0.095) | -0.098 (-0.24, 0.039) | 0.063 (-0.038, 0.16) | 0.0040 (-0.083, 0.091) | -0.0073 (-0.081, 0.067) | -0.006 (-0.0783, 0.066) |
|  | **RP disconnected** | -0.0018 (-0.18, 0.174) | -0.102 (-0.32, 0.122) | 0.016 (-0.106, 0.14) | 0.1351 (-0.015, 0.285) | 0.0104 (-0.114, 0.135) | 0.115 (-0.0093, 0.239) |

* The $\beta$ represents impact of covariates included in aggregate level matching (ALM) and reference prediction (RP) regression. The $\gamma$ represents impact of covariates not included in ALM or RP regression. Scenarios are analysis on RCTs only, using single-arm studies (single), and disconnected RCTs (disconnected).

Table 14 Estimated coverage probability (95% credible intervals) of simulation study using 100 simulations for random study effects models.*

|  |  | **5 RCTs** | | **15 RCTs** | | **50 RCTs** | |
| --- | --- | --- | --- | --- | --- | --- | --- |
|  |  | $\boldsymbol{\beta weak}$ | $\boldsymbol{\beta strong}$ | $\boldsymbol{\beta weak}$ | $\boldsymbol{\beta strong}$ | $\boldsymbol{\beta weak}$ | $\boldsymbol{\beta strong}$ |
| **Coverage on connected** $\boldsymbol{\gamma=0}$ | **RCT only** | 1.00 (1.00, 1) | 0.99 (0.98, 1) | 0.96 (0.92, 0.99) | 0.97 (0.95, 1) | 0.95 (0.93, 0.98) | 0.95 (0.92, 0.98) |
|  | **ALM single** | 1.00 (1.00, 1) | 0.99 (0.97, 1) | 0.98 (0.96, 1.00) | 0.99 (0.97, 1) | 0.96 (0.94, 0.99) | 0.94 (0.91, 0.97) |
|  | **ALM disconnected** | 0.99 (0.98, 1) | 0.99 (0.98, 1) | 0.96 (0.94, 0.99) | 0.99 (0.97, 1) | 0.96 (0.93, 0.99) | 0.95 (0.91, 0.98) |
|  | **RP single** | 0.99 (0.98, 1) | 0.99 (0.97, 1) | 0.97 (0.94, 0.99) | 0.99 (0.97, 1) | 0.95 (0.92, 0.98) | 0.95 (0.92, 0.98) |
|  | **RP disconnected** | 0.99 (0.98, 1) | 0.98 (0.96, 1) | 0.94 (0.90, 0.98) | 0.99 (0.97, 1) | 0.96 (0.94, 0.99) | 0.95 (0.92, 0.98) |
| **Coverage on disconnected** $\boldsymbol{\gamma=0}$ | **ALM single** | 0.58 (0.51, 0.64) | 0.69 (0.61, 0.77) | 0.52 (0.45, 0.60) | 0.52 (0.45, 0.58) | 0.49 (0.41, 0.57) | 0.42 (0.35, 0.48) |
|  | **ALM disconnected** | 0.86 (0.82, 0.90) | 0.83 (0.78, 0.87) | 0.73 (0.69, 0.78) | 0.69 (0.64, 0.74) | 0.72 (0.67, 0.77) | 0.73 (0.68, 0.78) |
|  | **RP single** | 0.74 (0.68, 0.81) | 0.80 (0.73, 0.86) | 0.84 (0.79, 0.90) | 0.81 (0.74, 0.87) | 0.83 (0.78, 0.89) | 0.79 (0.73, 0.85) |
|  | **RP disconnected** | 0.77 (0.69, 0.84) | 0.84 (0.78, 0.91) | 0.77 (0.70, 0.84) | 0.77 (0.70, 0.84) | 0.71 (0.63, 0.78) | 0.73 (0.66, 0.80) |
| **Coverage on connected** $\boldsymbol{\gamma}\boldsymbol{\neq0}$ | **RCT only** | 0.98 (0.97, 1) | 0.96 (0.93, 0.99) | 0.96 (0.93, 0.99) | 0.98 (0.96, 1.00) | 0.95 (0.92, 0.98) | 0.95 (0.93, 0.98) |
|  | **ALM single** | 0.98 (0.95, 1) | 0.98 (0.96, 1.00) | 0.98 (0.96, 1.00) | 0.97 (0.95, 1.00) | 0.94 (0.91, 0.97) | 0.94 (0.91, 0.98) |
|  | **ALM disconnected** | 0.98 (0.96, 1) | 0.99 (0.97, 1.00) | 0.97 (0.95, 0.99) | 0.97 (0.94, 1.00) | 0.94 (0.91, 0.97) | 0.94 (0.91, 0.98) |
|  | **RP single** | 0.99 (0.97, 1) | 0.99 (0.98, 1.00) | 0.98 (0.96, 1.00) | 0.98 (0.96, 1.00) | 0.96 (0.93, 0.98) | 0.95 (0.92, 0.98) |
|  | **RP disconnected** | 0.99 (0.97, 1) | 0.99 (0.96, 1.00) | 0.95 (0.92, 0.99) | 0.97 (0.95, 0.99) | 0.95 (0.92, 0.98) | 0.96 (0.93, 0.98) |
| **Coverage on disconnected** $\boldsymbol{\gamma}\boldsymbol{\neq0}$ | **ALM single** | 0.68 (0.62, 0.74) | 0.63 (0.55, 0.71) | 0.58 (0.51, 0.66) | 0.48 (0.41, 0.56) | 0.53 (0.46, 0.60) | 0.50 (0.42, 0.58) |
|  | **ALM disconnected** | 0.83 (0.78, 0.87) | 0.82 (0.77, 0.87) | 0.76 (0.71, 0.81) | 0.75 (0.70, 0.80) | 0.68 (0.63, 0.73) | 0.70 (0.65, 0.75) |
|  | **RP single** | 0.88 (0.83, 0.93) | 0.89 (0.85, 0.94) | 0.87 (0.82, 0.92) | 0.91 (0.87, 0.95) | 0.94 (0.90, 0.97) | 0.93 (0.88, 0.97) |
|  | **RP disconnected** | 0.87 (0.81, 0.92) | 0.84 (0.78, 0.90) | 0.84 (0.77, 0.90) | 0.82 (0.76, 0.88) | 0.88 (0.83, 0.93) | 0.85 (0.80, 0.90) |

* The $\beta$ represents impact of covariates included in aggregate level matching (ALM) and reference prediction (RP) regression. The $\gamma$ represents impact of covariates not included in ALM or RP regression. Scenarios are analysis on RCTs only, using single-arm studies (single), and disconnected RCTs (disconnected).

### A.3.3. Simulation study results for fixed study effects 100 patients per arm

Figure 12 Simulation study estimated bias and coverage of each method in the connected and disconnected evidence scenarios. Fixed study effects 100 patients per arm. Points are means and lines are 95% credible intervals. Line colours (indicated in “Bias connected”) are common across each of the four images.


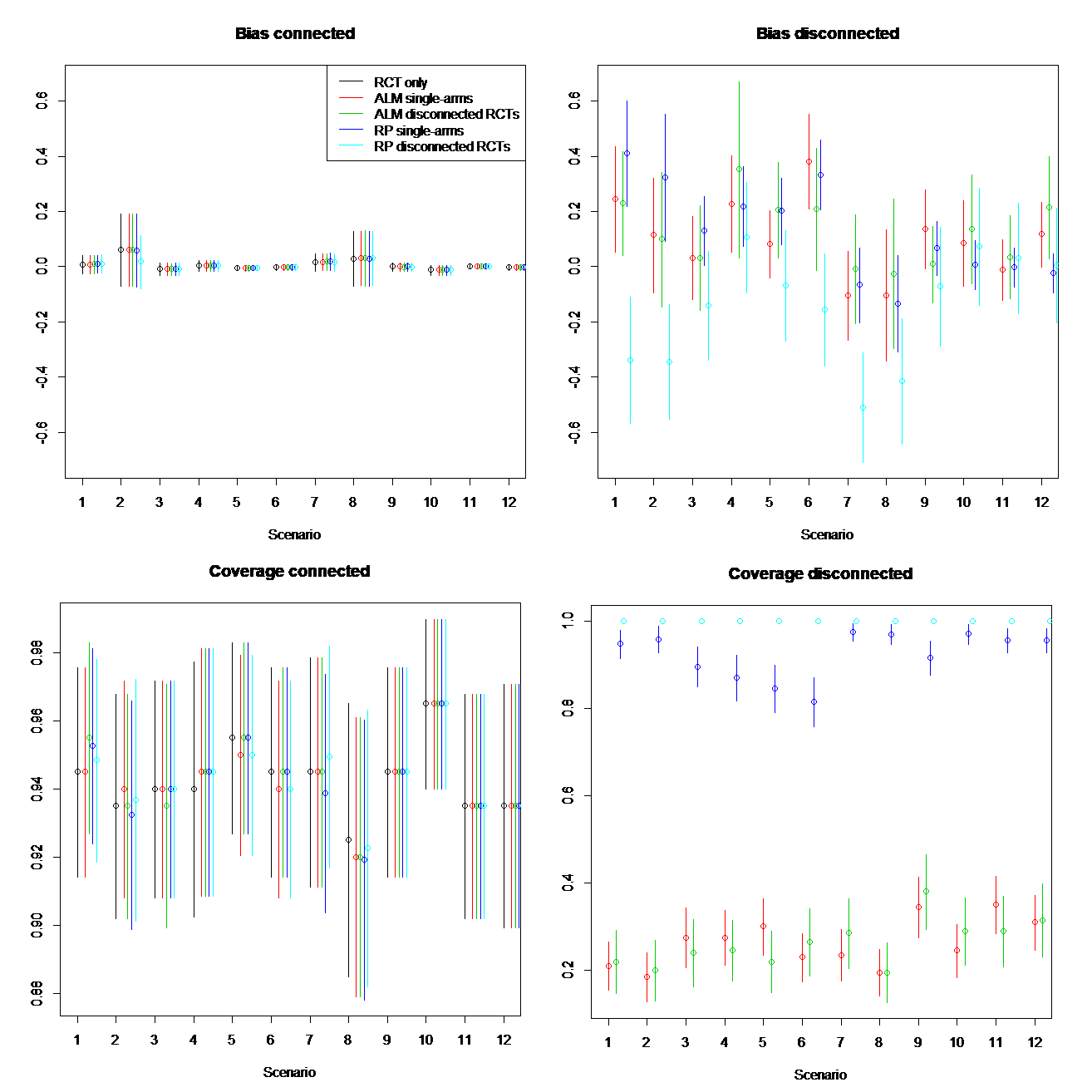


* Scenarios: 1=5 RCTs, $\beta$ weak, $\gamma=0$; 2=5 RCTs, $\beta$ strong, $\gamma=0$; 3=15 RCTs, $\beta$ weak, $\gamma=0$; 4=15 RCTs, $\beta$ strong, $\gamma=0$; 5=50 RCTs, $\beta$ weak, $\gamma=0$; 6=50 RCTs, $\beta$ strong, $\gamma=0$; 7=5 RCTs, $\beta$ weak, $\gamma\neq0$; 8=5 RCTs, $\beta$ strong, $\gamma\neq0$; 9=15 RCTs, $\beta$ weak, $\gamma\neq0$; 10=15 RCTs, $\beta$ strong, $\gamma\neq0$; 11=50 RCTs, $\beta$ weak, $\gamma\neq0$; 12=50 RCTs, $\beta$ strong, $\gamma\neq0$. The $\beta$ represents impact of covariates included in aggregate level matching (ALM) and reference prediction (RP) regression. The $\gamma$ represents impact of covariates not included in ALM or RP regression. Scenarios are analysis on RCTs only, using single-arm studies (single), and disconnected RCTs (disconnected).

Table 15 Estimated bias (95% credible interval) of simulation study using 100 simulations for fixed study effects models.*

|  |  | **5 RCTs** | | **15 RCTs** | | **50 RCTs** | |
| --- | --- | --- | --- | --- | --- | --- | --- |
|  |  | $\boldsymbol{\beta weak}$ | $\boldsymbol{\beta strong}$ | $\boldsymbol{\beta weak}$ | $\boldsymbol{\beta strong}$ | $\boldsymbol{\beta weak}$ | $\boldsymbol{\beta strong}$ |
| **Bias on connected** $\boldsymbol{\gamma=0}$ | **RCT only** | 0.0082 (-0.026, 0.043) | 0.061 (-0.071, 0.19) | -0.0074 (-0.030, 0.016) | 0.0053 (-0.016, 0.027) | -0.0038 (-0.015, 0.0074) | -0.0016 (-0.013, 0.01) |
|  | **ALM single** | 0.0086 (-0.026, 0.043) | 0.062 (-0.071, 0.20) | -0.0074 (-0.030, 0.015) | 0.0049 (-0.017, 0.027) | -0.0039 (-0.015, 0.0074) | -0.0016 (-0.013, 0.01) |
|  | **ALM disconnected** | 0.0095 (-0.025, 0.044) | 0.061 (-0.071, 0.19) | -0.0080 (-0.031, 0.015) | 0.0048 (-0.017, 0.027) | -0.0039 (-0.015, 0.0074) | -0.0015 (-0.013, 0.01) |
|  | **RP single** | 0.0102 (-0.022, 0.043) | 0.059 (-0.075, 0.19) | -0.0074 (-0.030, 0.016) | 0.0050 (-0.017, 0.027) | -0.0037 (-0.015, 0.0075) | -0.0015 (-0.013, 0.01) |
|  | **RP disconnected** | 0.0108 (-0.024, 0.045) | 0.019 (-0.080, 0.12) | -0.0079 (-0.031, 0.015) | 0.0048 (-0.017, 0.027) | -0.0037 (-0.015, 0.0075) | -0.0015 (-0.013, 0.01) |
| **Bias on disconnected** $\boldsymbol{\gamma=0}$ | **ALM single** | 0.25 ( 0.053, 0.44) | 0.12 (-0.095, 0.32) | 0.033 (-0.1203, 0.186) | 0.23 ( 0.052, 0.41) | 0.084 (-0.040, 0.21) | 0.38 ( 0.208, 0.554) |
|  | **ALM disconnected** | 0.23 ( 0.041, 0.42) | 0.10 (-0.145, 0.34) | 0.032 (-0.1589, 0.224) | 0.35 ( 0.031, 0.67) | 0.207 ( 0.033, 0.38) | 0.21 (-0.013, 0.432) |
|  | **RP single** | 0.41 ( 0.218, 0.60) | 0.32 ( 0.092, 0.56) | 0.131 ( 0.0059, 0.257) | 0.22 ( 0.073, 0.36) | 0.202 ( 0.080, 0.32) | 0.33 ( 0.205, 0.462) |
|  | **RP disconnected** | -0.34 (-0.569, -0.11) | -0.34 (-0.553, -0.13) | -0.141 (-0.3401, 0.057) | 0.11 (-0.095, 0.31) | -0.068 (-0.269, 0.13) | -0.15 (-0.359, 0.049) |
| **Bias on connected** $\boldsymbol{\gamma\neq0}$ | **RCT only** | 0.017 (-0.015, 0.050) | 0.030 (-0.070, 0.13) | 3.8e-04 (-0.017, 0.018) | -0.011 (-0.031, 0.0084) | 0.00069 (-0.0085, 0.0099) | -0.00080 (-0.012, 0.011) |
|  | **ALM single** | 0.017 (-0.015, 0.050) | 0.031 (-0.069, 0.13) | 1.9e-05 (-0.017, 0.017) | -0.012 (-0.032, 0.0082) | 0.00062 (-0.0086, 0.0098) | -0.00089 (-0.012, 0.011) |
|  | **ALM disconnected** | 0.018 (-0.014, 0.051) | 0.033 (-0.069, 0.13) | -9.2e-05 (-0.017, 0.017) | -0.012 (-0.032, 0.0078) | 0.00066 (-0.0086, 0.0099) | -0.00088 (-0.012, 0.011) |
|  | **RP single** | 0.019 (-0.014, 0.051) | 0.030 (-0.070, 0.13) | 3.1e-04 (-0.017, 0.018) | -0.012 (-0.032, 0.0082) | 0.00071 (-0.0086, 0.0100) | -0.00080 (-0.012, 0.011) |
|  | **RP disconnected** | 0.016 (-0.016, 0.049) | 0.032 (-0.069, 0.13) | -2.8e-04 (-0.018, 0.017) | -0.012 (-0.032, 0.0079) | 0.00078 (-0.0085, 0.0100) | -0.00082 (-0.012, 0.011) |
| **Bias on disconnected** $\boldsymbol{\gamma\neq0}$ | **ALM single** | -0.1037 (-0.27, 0.059) | -0.103 (-0.34, 0.136) | 0.1372 (-0.0073, 0.28) | 0.0865 (-0.070, 0.243) | -0.0102 (-0.122, 0.102) | 0.1181 (-0.0015, 0.238) |
|  | **ALM disconnected** | -0.0068 (-0.20, 0.191) | -0.025 (-0.30, 0.248) | 0.0098 (-0.1305, 0.15) | 0.1361 (-0.062, 0.335) | 0.0357 (-0.117, 0.188) | 0.2150 ( 0.0283, 0.402) |
|  | **RP single** | -0.0656 (-0.20, 0.072) | -0.133 (-0.31, 0.043) | 0.0687 (-0.0307, 0.17) | 0.0075 (-0.083, 0.098) | -0.0011 (-0.074, 0.072) | -0.0227 (-0.0937, 0.048) |
|  | **RP disconnected** | -0.5098 (-0.71, -0.310) | -0.413 (-0.64, -0.184) | -0.0712 (-0.2890, 0.15) | 0.0739 (-0.139, 0.287) | 0.0311 (-0.170, 0.233) | 0.0052 (-0.2046, 0.215) |

* The $\beta$ represents impact of covariates included in aggregate level matching (ALM) and reference prediction (RP) regression. The $\gamma$ represents impact of covariates not included in ALM or RP regression. Scenarios are analysis on RCTs only, using single-arm studies (single), and disconnected RCTs (disconnected).

Table 16 Estimated coverage probability (95% credible interval) of simulation study using 100 simulations for fixed study effects models.*

|  |  | **5 RCTs** | | **15 RCTs** | | **50 RCTs** | |
| --- | --- | --- | --- | --- | --- | --- | --- |
|  |  | $\boldsymbol{\beta weak}$ | $\boldsymbol{\beta strong}$ | $\boldsymbol{\beta weak}$ | $\boldsymbol{\beta strong}$ | $\boldsymbol{\beta weak}$ | $\boldsymbol{\beta strong}$ |
| **Coverage on connected** $\boldsymbol{\gamma=0}$ | **RCT only** | 0.94 (0.91, 0.98) | 0.94 (0.90, 0.97) | 0.94 (0.91, 0.97) | 0.94 (0.90, 0.98) | 0.95 (0.93, 0.98) | 0.94 (0.91, 0.98) |
|  | **ALM single** | 0.94 (0.91, 0.98) | 0.94 (0.91, 0.97) | 0.94 (0.91, 0.97) | 0.94 (0.91, 0.98) | 0.95 (0.92, 0.98) | 0.94 (0.91, 0.97) |
|  | **ALM disconnected** | 0.95 (0.93, 0.98) | 0.94 (0.90, 0.97) | 0.94 (0.90, 0.97) | 0.94 (0.91, 0.98) | 0.95 (0.93, 0.98) | 0.94 (0.91, 0.98) |
|  | **RP single** | 0.95 (0.92, 0.98) | 0.93 (0.90, 0.97) | 0.94 (0.91, 0.97) | 0.94 (0.91, 0.98) | 0.95 (0.93, 0.98) | 0.94 (0.91, 0.98) |
|  | **RP disconnected** | 0.95 (0.92, 0.98) | 0.94 (0.90, 0.97) | 0.94 (0.91, 0.97) | 0.94 (0.91, 0.98) | 0.95 (0.92, 0.98) | 0.94 (0.91, 0.97) |
| **Coverage on disconnected** $\boldsymbol{\gamma=0}$ | **ALM single** | 0.21 (0.15, 0.27) | 0.18 (0.13, 0.24) | 0.28 (0.21, 0.34) | 0.28 (0.21, 0.34) | 0.30 (0.23, 0.37) | 0.23 (0.17, 0.29) |
|  | **ALM disconnected** | 0.22 (0.15, 0.29) | 0.20 (0.13, 0.27) | 0.24 (0.16, 0.32) | 0.24 (0.17, 0.32) | 0.22 (0.15, 0.29) | 0.26 (0.19, 0.34) |
|  | **RP single** | 0.95 (0.91, 0.98) | 0.96 (0.93, 0.99) | 0.90 (0.85, 0.94) | 0.87 (0.82, 0.92) | 0.84 (0.79, 0.90) | 0.81 (0.76, 0.87) |
|  | **RP disconnected** | 1.00 (1.00, 1.00) | 1.00 (1.00, 1.00) | 1.00 (1.00, 1.00) | 1.00 (1.00, 1.00) | 1.00 (1.00, 1.00) | 1.00 (1.00, 1.00) |
| **Coverage on connected** $\boldsymbol{\gamma\neq0}$ | **RCT only** | 0.94 (0.91, 0.98) | 0.92 (0.88, 0.97) | 0.94 (0.91, 0.98) | 0.96 (0.94, 0.99) | 0.94 (0.9, 0.97) | 0.94 (0.9, 0.97) |
|  | **ALM single** | 0.94 (0.91, 0.98) | 0.92 (0.88, 0.96) | 0.94 (0.91, 0.98) | 0.96 (0.94, 0.99) | 0.94 (0.9, 0.97) | 0.94 (0.9, 0.97) |
|  | **ALM disconnected** | 0.94 (0.91, 0.98) | 0.92 (0.88, 0.96) | 0.94 (0.91, 0.98) | 0.96 (0.94, 0.99) | 0.94 (0.9, 0.97) | 0.94 (0.9, 0.97) |
|  | **RP single** | 0.94 (0.90, 0.97) | 0.92 (0.88, 0.96) | 0.94 (0.91, 0.98) | 0.96 (0.94, 0.99) | 0.94 (0.9, 0.97) | 0.94 (0.9, 0.97) |
|  | **RP disconnected** | 0.95 (0.92, 0.98) | 0.92 (0.88, 0.96) | 0.94 (0.91, 0.98) | 0.96 (0.94, 0.99) | 0.94 (0.9, 0.97) | 0.94 (0.9, 0.97) |
| **Coverage on disconnected** $\boldsymbol{\gamma\neq0}$ | **ALM single** | 0.23 (0.18, 0.29) | 0.20 (0.14, 0.25) | 0.34 (0.27, 0.42) | 0.24 (0.18, 0.31) | 0.35 (0.28, 0.42) | 0.31 (0.25, 0.37) |
|  | **ALM disconnected** | 0.28 (0.20, 0.37) | 0.20 (0.13, 0.26) | 0.38 (0.29, 0.47) | 0.29 (0.21, 0.37) | 0.29 (0.21, 0.37) | 0.32 (0.23, 0.40) |
|  | **RP single** | 0.97 (0.95, 1.00) | 0.97 (0.95, 0.99) | 0.92 (0.88, 0.95) | 0.97 (0.95, 0.99) | 0.95 (0.93, 0.98) | 0.95 (0.93, 0.98) |
|  | **RP disconnected** | 1.00 (1.00, 1.00) | 1.00 (1.00, 1.00) | 1.00 (1.00, 1.00) | 1.00 (1.00, 1.00) | 1.00 (1.00, 1.00) | 1.00 (1.00, 1.00) |

* The $\beta$ represents impact of covariates included in aggregate level matching (ALM) and reference prediction (RP) regression. The $\gamma$ represents impact of covariates not included in ALM or RP regression. Scenarios are analysis on RCTs only, using single-arm studies (single), and disconnected RCTs (disconnected).

### A.3.4. Further simulation study results for random study effects 1000 patients per arm

Table 17 Estimated bias (95% credible interval) of simulation study using 1000 simulations for random study effects models.*

|  |  | **5 RCTs** | | **15 RCTs** | | **50 RCTs** | |
| --- | --- | --- | --- | --- | --- | --- | --- |
|  |  | $\boldsymbol{\beta weak}$ | $\boldsymbol{\beta strong}$ | $\boldsymbol{\beta weak}$ | $\boldsymbol{\beta strong}$ | $\boldsymbol{\beta weak}$ | $\boldsymbol{\beta strong}$ |
| **Bias on connected** $\boldsymbol{\gamma=0}$ | **RCT only** | -0.0066 (-0.017, 0.0038) | -0.0106 (-0.026, 0.0050) | -0.0059 (-0.0108, -0.00099) | 0.00150 (-0.0044, 0.0074) | 0.0029 (-0.00052, 0.0062) | -3.6e-04 (-0.0038, 0.0030) |
|  | **ALM single** | -0.0074 (-0.018, 0.0032) | -0.0059 (-0.022, 0.0098) | -0.0066 (-0.0115, -0.00169) | -0.00095 (-0.0068, 0.0049) | 0.0026 (-0.00088, 0.0060) | -5.5e-04 (-0.0039, 0.0028) |
|  | **ALM disconnected** | -0.0053 (-0.017, 0.0064) | -0.0024 (-0.019, 0.0138) | -0.0054 (-0.0099, -0.00096) | 0.00055 (-0.0054, 0.0065) | 0.0020 (-0.00126, 0.0052) | -5.1e-05 (-0.0032, 0.0031) |
|  | **RP single** | -0.0077 (-0.019, 0.0032) | -0.0071 (-0.023, 0.0085) | -0.0071 (-0.0118, -0.00239) | 0.00386 (-0.0023, 0.0101) | 0.0024 (-0.00111, 0.0058) | -2.7e-04 (-0.0035, 0.0029) |
|  | **RP disconnected** | -0.0094 (-0.020, 0.0011) | -0.0058 (-0.024, 0.0120) | -0.0058 (-0.0105, -0.00107) | 0.00051 (-0.0054, 0.0064) | 0.0029 (-0.00040, 0.0063) | 2.0e-04 (-0.0032, 0.0036) |
| **Bias on disconnected** $\boldsymbol{\gamma=0}$ | **ALM single** | 0.024 (-0.217, 0.26) | 0.084 (-0.1568, 0.32) | 0.27 (0.136, 0.40) | 0.0053 (-0.168, 0.18) | 0.068 (-0.0794, 0.22) | 0.059 (-0.096, 0.21) |
|  | **ALM disconnected** | 0.021 (-0.156, 0.20) | 0.051 (-0.0997, 0.20) | 0.21 (0.048, 0.37) | 0.0509 (-0.109, 0.21) | 0.167 ( 0.0346, 0.30) | 0.033 (-0.152, 0.22) |
|  | **RP single** | 0.030 (-0.137, 0.20) | 0.190 ( 0.0012, 0.38) | 0.28 (0.172, 0.40) | 0.1016 (-0.035, 0.24) | 0.132 ( 0.0026, 0.26) | 0.189 ( 0.064, 0.32) |
|  | **RP disconnected** | 0.228 ( 0.024, 0.43) | 0.151 (-0.0930, 0.40) | 0.24 (0.066, 0.41) | 0.1774 (-0.016, 0.37) | 0.354 ( 0.1725, 0.53) | 0.191 (-0.065, 0.45) |
| **Bias on connected** $\boldsymbol{\gamma\neq0}$ | **RCT only** | 0.0061 (-0.0068, 0.019) | 0.00035 (-0.0121, 0.013) | 0.0032 (-0.0030, 0.0093) | 0.0029 (-0.00402, 0.0099) | 0.00011 (-0.0029, 0.0031) | -0.00073 (-0.0042, 0.0027) |
|  | **ALM single** | 0.0035 (-0.0099, 0.017) | 0.00224 (-0.0090, 0.013) | 0.0023 (-0.0037, 0.0083) | 0.0030 (-0.00393, 0.0099) | -0.00038 (-0.0034, 0.0027) | -0.00021 (-0.0037, 0.0033) |
|  | **ALM disconnected** | 0.0124 (-0.0011, 0.026) | 0.00513 (-0.0066, 0.017) | 0.0015 (-0.0048, 0.0079) | 0.0042 (-0.00276, 0.0112) | 0.00039 (-0.0027, 0.0035) | -0.00093 (-0.0044, 0.0026) |
|  | **RP single** | 0.0034 (-0.0104, 0.017) | 0.00428 (-0.0069, 0.015) | 0.0038 (-0.0023, 0.0099) | 0.0057 (-0.00091, 0.0124) | 0.00023 (-0.0028, 0.0033) | -0.00102 (-0.0046, 0.0026) |
|  | **RP disconnected** | 0.0107 (-0.0043, 0.026) | 0.00831 (-0.0029, 0.020) | 0.0033 (-0.0031, 0.0097) | 0.0026 (-0.00400, 0.0092) | 0.00034 (-0.0027, 0.0034) | -0.00050 (-0.0041, 0.0031) |
| **Bias on disconnected** $\boldsymbol{\gamma\neq0}$ | **ALM single** | 0.076 (-0.095, 0.25) | -0.101 (-0.29, 0.088) | -0.117 (-0.24, 0.006) | -0.029 (-0.179, 0.120) | 0.061 (-0.053, 0.175) | 0.1174 (-0.029, 0.263) |
|  | **ALM disconnected** | -0.057 (-0.249, 0.13) | -0.013 (-0.17, 0.148) | -0.084 (-0.23, 0.058) | 0.063 (-0.095, 0.221) | -0.040 (-0.220, 0.141) | 0.1732 ( 0.033, 0.313) |
|  | **RP single** | 0.008 (-0.118, 0.13) | 0.021 (-0.10, 0.145) | -0.042 (-0.13, 0.049) | 0.073 (-0.017, 0.164) | -0.025 (-0.095, 0.045) | 0.0097 (-0.059, 0.079) |
|  | **RP disconnected** | 0.047 (-0.083, 0.18) | -0.025 (-0.19, 0.139) | 0.021 (-0.11, 0.156) | -0.056 (-0.189, 0.078) | -0.049 (-0.162, 0.065) | 0.1347 ( 0.015, 0.254) |

* The $\beta$ represents impact of covariates included in aggregate level matching (ALM) and reference prediction (RP) regression. The $\gamma$ represents impact of covariates not included in ALM or RP regression. Scenarios are analysis on RCTs only, using single-arm studies (single), and disconnected RCTs (disconnected).

Table 18 Estimated coverage probability (95% credible interval) of simulation study using 1000 simulations for random study effects models.*

|  |  | **5 RCTs** | | **15 RCTs** | | **50 RCTs** | |
| --- | --- | --- | --- | --- | --- | --- | --- |
|  |  | $\boldsymbol{\beta weak}$ | $\boldsymbol{\beta strong}$ | $\boldsymbol{\beta weak}$ | $\boldsymbol{\beta strong}$ | $\boldsymbol{\beta weak}$ | $\boldsymbol{\beta strong}$ |
| **Coverage on connected** $\boldsymbol{\gamma=0}$ | **RCT only** | 0.99 (0.98, 1) | 0.95 (0.91, 0.99) | 0.96 (0.93, 0.99) | 0.95 (0.92, 0.99) | 0.95 (0.91, 0.98) | 0.96 (0.92, 0.99) |
|  | **ALM single** | 0.98 (0.96, 1) | 0.99 (0.97, 1.00) | 0.95 (0.91, 0.98) | 0.95 (0.93, 0.98) | 0.94 (0.90, 0.98) | 0.92 (0.87, 0.96) |
|  | **ALM disconnected** | 0.98 (0.96, 1) | 0.99 (0.98, 1.00) | 0.95 (0.91, 0.98) | 0.93 (0.89, 0.97) | 0.95 (0.91, 0.98) | 0.96 (0.93, 0.98) |
|  | **RP single** | 0.97 (0.94, 1) | 1.00 (1.00, 1.00) | 0.97 (0.94, 0.99) | 0.93 (0.88, 0.97) | 0.94 (0.91, 0.98) | 0.95 (0.91, 0.98) |
|  | **RP disconnected** | 0.99 (0.98, 1) | 0.98 (0.95, 1.00) | 0.98 (0.96, 1.00) | 0.95 (0.92, 0.98) | 0.93 (0.90, 0.97) | 0.95 (0.92, 0.98) |
| **Coverage on disconnected** $\boldsymbol{\gamma=0}$ | **ALM single** | 0.40 (0.33, 0.48) | 0.50 (0.43, 0.57) | 0.39 (0.33, 0.45) | 0.31 (0.24, 0.38) | 0.30 (0.24, 0.36) | 0.26 (0.19, 0.32) |
|  | **ALM disconnected** | 0.72 (0.67, 0.77) | 0.76 (0.71, 0.81) | 0.63 (0.59, 0.68) | 0.64 (0.60, 0.69) | 0.60 (0.56, 0.64) | 0.64 (0.60, 0.69) |
|  | **RP single** | 0.74 (0.67, 0.80) | 0.76 (0.70, 0.83) | 0.77 (0.72, 0.83) | 0.68 (0.61, 0.75) | 0.80 (0.73, 0.86) | 0.80 (0.74, 0.86) |
|  | **RP disconnected** | 0.69 (0.61, 0.78) | 0.72 (0.65, 0.80) | 0.64 (0.56, 0.72) | 0.68 (0.60, 0.76) | 0.73 (0.65, 0.80) | 0.78 (0.72, 0.84) |
| **Coverage on connected** $\boldsymbol{\gamma\neq0}$ | **RCT only** | 0.99 (0.97, 1) | 0.97 (0.94, 1) | 0.96 (0.94, 0.99) | 0.94 (0.90, 0.98) | 0.96 (0.93, 0.99) | 0.96 (0.93, 0.98) |
|  | **ALM single** | 1.00 (1.00, 1) | 0.98 (0.96, 1) | 0.95 (0.92, 0.98) | 0.96 (0.93, 0.99) | 0.95 (0.93, 0.98) | 0.96 (0.93, 0.99) |
|  | **ALM disconnected** | 0.98 (0.95, 1) | 0.99 (0.98, 1) | 0.93 (0.89, 0.97) | 0.94 (0.91, 0.97) | 0.95 (0.91, 0.98) | 0.96 (0.93, 0.98) |
|  | **RP single** | 1.00 (1.00, 1) | 0.98 (0.95, 1) | 0.96 (0.93, 0.99) | 0.93 (0.89, 0.97) | 0.96 (0.93, 0.99) | 0.94 (0.91, 0.98) |
|  | **RP disconnected** | 0.98 (0.97, 1) | 1.00 (1.00, 1) | 0.96 (0.93, 0.99) | 0.92 (0.89, 0.96) | 0.96 (0.94, 0.99) | 0.93 (0.89, 0.97) |
| **Coverage on disconnected** $\boldsymbol{\gamma\neq0}$ | **ALM single** | 0.54 (0.46, 0.62) | 0.46 (0.39, 0.54) | 0.36 (0.29, 0.43) | 0.32 (0.25, 0.39) | 0.30 (0.24, 0.36) | 0.32 (0.26, 0.38) |
|  | **ALM disconnected** | 0.73 (0.68, 0.77) | 0.75 (0.70, 0.80) | 0.67 (0.63, 0.72) | 0.63 (0.59, 0.67) | 0.67 (0.62, 0.72) | 0.66 (0.61, 0.70) |
|  | **RP single** | 0.78 (0.72, 0.84) | 0.78 (0.72, 0.85) | 0.85 (0.80, 0.90) | 0.86 (0.80, 0.91) | 0.89 (0.84, 0.94) | 0.92 (0.89, 0.96) |
|  | **RP disconnected** | 0.90 (0.86, 0.94) | 0.81 (0.75, 0.87) | 0.86 (0.80, 0.91) | 0.77 (0.70, 0.84) | 0.85 (0.79, 0.90) | 0.79 (0.73, 0.86) |

* The $\beta$ represents impact of covariates included in aggregate level matching (ALM) and reference prediction (RP) regression. The $\gamma$ represents impact of covariates not included in ALM or RP regression. Scenarios are analysis on RCTs only, using single-arm studies (single), and disconnected RCTs (disconnected).

### A.3.5. Simulation study results for fixed study effects 1000 patients per arm

Figure 12 Simulation study estimated bias and coverage of each method in the connected and disconnected evidence scenarios. Fixed study effects 1000 patients per arm. Points are means and lines are 95% credible intervals. Line colours (indicated in “Bias connected”) are common across each of the four images.


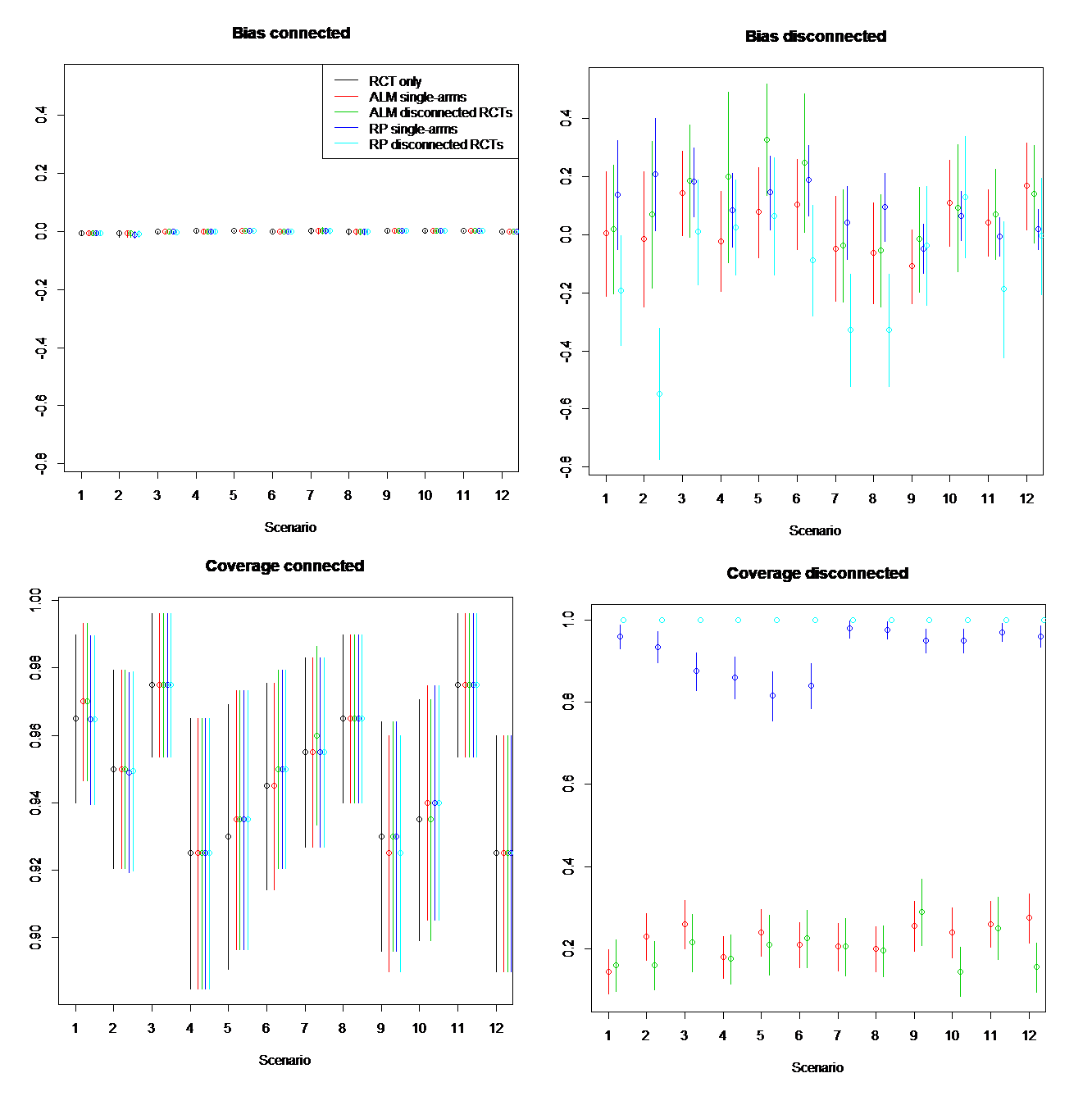


* Scenarios: 1=5 RCTs, $\beta$ weak, $\gamma=0$; 2=5 RCTs, $\beta$ strong, $\gamma=0$; 3=15 RCTs, $\beta$ weak, $\gamma=0$; 4=15 RCTs, $\beta$ strong, $\gamma=0$; 5=50 RCTs, $\beta$ weak, $\gamma=0$; 6=50 RCTs, $\beta$ strong, $\gamma=0$; 7=5 RCTs, $\beta$ weak, $\gamma\neq0$; 8=5 RCTs, $\beta$ strong, $\gamma\neq0$; 9=15 RCTs, $\beta$ weak, $\gamma\neq0$; 10=15 RCTs, $\beta$ strong, $\gamma\neq0$; 11=50 RCTs, $\beta$ weak, $\gamma\neq0$; 12=50 RCTs, $\beta$ strong, $\gamma\neq0$. The $\beta$ represents impact of covariates included in aggregate level matching (ALM) and reference prediction (RP) regression. The $\gamma$ represents impact of covariates not included in ALM or RP regression. Scenarios are analysis on RCTs only, using single-arm studies (single), and disconnected RCTs (disconnected).

Table 19 Estimated bias (95% credible interval) of simulation study using 1000 simulations for fixed study effects models.*

|  |  | **5 RCTs** | | **15 RCTs** | | **50 RCTs** | |
| --- | --- | --- | --- | --- | --- | --- | --- |
|  |  | $\boldsymbol{\beta weak}$ | $\boldsymbol{\beta strong}$ | $\boldsymbol{\beta weak}$ | $\boldsymbol{\beta strong}$ | $\boldsymbol{\beta weak}$ | $\boldsymbol{\beta strong}$ |
| **Bias on connected** $\boldsymbol{\gamma=0}$ | **RCT only** | -0.0070 (-0.016, 0.0019) | -0.0079 (-0.021, 0.00531) | -0.0025 (-0.0072, 0.0021) | 1.8e-04 (-0.0063, 0.0066) | 0.0023 (-0.00096, 0.0056) | -0.00039 (-0.0038, 0.0030) |
|  | **ALM single** | -0.0067 (-0.016, 0.0023) | -0.0076 (-0.021, 0.00561) | -0.0026 (-0.0072, 0.0021) | 1.1e-04 (-0.0063, 0.0066) | 0.0023 (-0.00100, 0.0056) | -0.00044 (-0.0038, 0.0029) |
|  | **ALM disconnected** | -0.0066 (-0.016, 0.0024) | -0.0076 (-0.021, 0.00561) | -0.0027 (-0.0073, 0.0020) | -2.0e-05 (-0.0065, 0.0064) | 0.0023 (-0.00098, 0.0056) | -0.00042 (-0.0038, 0.0030) |
|  | **RP single** | -0.0066 (-0.016, 0.0024) | -0.0114 (-0.024, 0.00062) | -0.0026 (-0.0072, 0.0021) | 1.1e-04 (-0.0064, 0.0066) | 0.0024 (-0.00092, 0.0057) | -0.00041 (-0.0038, 0.0030) |
|  | **RP disconnected** | -0.0067 (-0.016, 0.0023) | -0.0090 (-0.022, 0.00400) | -0.0027 (-0.0074, 0.0019) | -3.1e-06 (-0.0065, 0.0064) | 0.0024 (-0.00093, 0.0056) | -0.00038 (-0.0038, 0.0030) |
| **Bias on disconnected** $\boldsymbol{\gamma=0}$ | **ALM single** | 0.0032 (-0.213, 0.2197) | -0.015 (-0.250, 0.22) | 0.1418 (-0.0049, 0.29) | -0.023 (-0.195, 0.15) | 0.077 (-0.079, 0.23) | 0.104 (-0.0532, 0.26) |
|  | **ALM disconnected** | 0.0182 (-0.205, 0.2412) | 0.069 (-0.185, 0.32) | 0.1845 (-0.0107, 0.38) | 0.197 (-0.098, 0.49) | 0.327 ( 0.133, 0.52) | 0.247 ( 0.0082, 0.49) |
|  | **RP single** | 0.1367 (-0.053, 0.3259) | 0.207 ( 0.013, 0.40) | 0.1809 ( 0.0614, 0.30) | 0.084 (-0.044, 0.21) | 0.144 ( 0.015, 0.27) | 0.186 ( 0.0647, 0.31) |
|  | **RP disconnected** | -0.1919 (-0.382, -0.0014) | -0.547 (-0.774, -0.32) | 0.0095 (-0.1726, 0.19) | 0.025 (-0.140, 0.19) | 0.064 (-0.139, 0.27) | -0.089 (-0.2810, 0.10) |
| **Bias on connected** $\boldsymbol{\gamma\neq0}$ | **RCT only** | 0.0018 (-0.0103, 0.014) | -0.00109 (-0.012, 0.010) | 0.0023 (-0.0037, 0.0082) | 0.0012 (-0.0054, 0.0079) | 0.00027 (-0.0027, 0.0032) | -0.00101 (-0.0044, 0.0024) |
|  | **ALM single** | 0.0019 (-0.0102, 0.014) | -0.00087 (-0.012, 0.010) | 0.0022 (-0.0038, 0.0081) | 0.0012 (-0.0054, 0.0079) | 0.00022 (-0.0027, 0.0032) | -0.00102 (-0.0044, 0.0024) |
|  | **ALM disconnected** | 0.0023 (-0.0099, 0.014) | -0.00070 (-0.012, 0.011) | 0.0021 (-0.0039, 0.0080) | 0.0011 (-0.0056, 0.0077) | 0.00025 (-0.0027, 0.0032) | -0.00098 (-0.0044, 0.0024) |
|  | **RP single** | 0.0020 (-0.0102, 0.014) | -0.00106 (-0.012, 0.010) | 0.0022 (-0.0038, 0.0081) | 0.0013 (-0.0053, 0.0080) | 0.00027 (-0.0027, 0.0032) | -0.00094 (-0.0044, 0.0025) |
|  | **RP disconnected** | 0.0017 (-0.0104, 0.014) | -0.00127 (-0.013, 0.010) | 0.0021 (-0.0039, 0.0080) | 0.0011 (-0.0055, 0.0078) | 0.00023 (-0.0027, 0.0032) | -0.00097 (-0.0044, 0.0024) |
| **Bias on disconnected** $\boldsymbol{\gamma\neq0}$ | **ALM single** | -0.048 (-0.229, 0.13) | -0.064 (-0.239, 0.11) | -0.109 (-0.24, 0.020) | 0.109 (-0.04, 0.26) | 0.0398 (-0.076, 0.155) | 0.1667 ( 0.016, 0.32) |
|  | **ALM disconnected** | -0.037 (-0.232, 0.16) | -0.056 (-0.251, 0.14) | -0.016 (-0.20, 0.165) | 0.090 (-0.13, 0.31) | 0.0698 (-0.087, 0.226) | 0.1392 (-0.030, 0.31) |
|  | **RP single** | 0.040 (-0.087, 0.17) | 0.095 (-0.023, 0.21) | -0.048 (-0.13, 0.037) | 0.065 (-0.02, 0.15) | -0.0067 (-0.074, 0.061) | 0.0189 (-0.052, 0.09) |
|  | **RP disconnected** | -0.328 (-0.523, -0.13) | -0.328 (-0.523, -0.13) | -0.039 (-0.24, 0.166) | 0.129 (-0.08, 0.34) | -0.1886 (-0.423, 0.046) | -0.0048 (-0.206, 0.20) |

* The $\beta$ represents impact of covariates included in aggregate level matching (ALM) and reference prediction (RP) regression. The $\gamma$ represents impact of covariates not included in ALM or RP regression. Scenarios are analysis on RCTs only, using single-arm studies (single), and disconnected RCTs (disconnected).

Table 20 Estimated coverage probability (95% credible interval) of simulation study using 1000 simulations for fixed study effects models.*

|  |  | **5 RCTs** | | **15 RCTs** | | **50 RCTs** | |
| --- | --- | --- | --- | --- | --- | --- | --- |
|  |  | $\boldsymbol{\beta weak}$ | $\boldsymbol{\beta strong}$ | $\boldsymbol{\beta weak}$ | $\boldsymbol{\beta strong}$ | $\boldsymbol{\beta weak}$ | $\boldsymbol{\beta strong}$ |
| **Coverage on connected** $\boldsymbol{\gamma=0}$ | **RCT only** | 0.96 (0.94, 0.99) | 0.95 (0.92, 0.98) | 0.97 (0.95, 1) | 0.92 (0.88, 0.97) | 0.93 (0.89, 0.97) | 0.94 (0.91, 0.98) |
|  | **ALM single** | 0.97 (0.95, 0.99) | 0.95 (0.92, 0.98) | 0.97 (0.95, 1) | 0.92 (0.88, 0.97) | 0.94 (0.90, 0.97) | 0.94 (0.91, 0.98) |
|  | **ALM disconnected** | 0.97 (0.95, 0.99) | 0.95 (0.92, 0.98) | 0.97 (0.95, 1) | 0.92 (0.88, 0.97) | 0.94 (0.90, 0.97) | 0.95 (0.92, 0.98) |
|  | **RP single** | 0.96 (0.94, 0.99) | 0.95 (0.92, 0.98) | 0.97 (0.95, 1) | 0.92 (0.88, 0.97) | 0.94 (0.90, 0.97) | 0.95 (0.92, 0.98) |
|  | **RP disconnected** | 0.96 (0.94, 0.99) | 0.95 (0.92, 0.98) | 0.97 (0.95, 1) | 0.92 (0.88, 0.97) | 0.94 (0.90, 0.97) | 0.95 (0.92, 0.98) |
| **Coverage on disconnected** $\boldsymbol{\gamma=0}$ | **ALM single** | 0.14 (0.091, 0.20) | 0.23 (0.17, 0.29) | 0.26 (0.20, 0.32) | 0.18 (0.13, 0.23) | 0.24 (0.18, 0.30) | 0.21 (0.15, 0.27) |
|  | **ALM disconnected** | 0.16 (0.096, 0.22) | 0.16 (0.10, 0.22) | 0.21 (0.15, 0.28) | 0.17 (0.11, 0.24) | 0.21 (0.14, 0.28) | 0.22 (0.15, 0.30) |
|  | **RP single** | 0.96 (0.929, 0.99) | 0.93 (0.89, 0.97) | 0.88 (0.83, 0.92) | 0.86 (0.81, 0.91) | 0.81 (0.75, 0.88) | 0.84 (0.78, 0.90) |
|  | **RP disconnected** | 1.00 (1.000, 1.00) | 1.00 (1.00, 1.00) | 1.00 (1.00, 1.00) | 1.00 (1.00, 1.00) | 1.00 (1.00, 1.00) | 1.00 (1.00, 1.00) |
| **Coverage on connected** $\boldsymbol{\gamma\neq0}$ | **RCT only** | 0.95 (0.93, 0.98) | 0.96 (0.94, 0.99) | 0.93 (0.90, 0.96) | 0.94 (0.90, 0.97) | 0.97 (0.95, 1) | 0.92 (0.89, 0.96) |
|  | **ALM single** | 0.95 (0.93, 0.98) | 0.96 (0.94, 0.99) | 0.92 (0.89, 0.96) | 0.94 (0.91, 0.97) | 0.97 (0.95, 1) | 0.92 (0.89, 0.96) |
|  | **ALM disconnected** | 0.96 (0.93, 0.99) | 0.96 (0.94, 0.99) | 0.93 (0.90, 0.96) | 0.94 (0.90, 0.97) | 0.97 (0.95, 1) | 0.92 (0.89, 0.96) |
|  | **RP single** | 0.95 (0.93, 0.98) | 0.96 (0.94, 0.99) | 0.93 (0.90, 0.96) | 0.94 (0.91, 0.97) | 0.97 (0.95, 1) | 0.92 (0.89, 0.96) |
|  | **RP disconnected** | 0.95 (0.93, 0.98) | 0.96 (0.94, 0.99) | 0.92 (0.89, 0.96) | 0.94 (0.91, 0.97) | 0.97 (0.95, 1) | 0.92 (0.89, 0.96) |
| **Coverage on disconnected** $\boldsymbol{\gamma\neq0}$ | **ALM single** | 0.20 (0.15, 0.26) | 0.20 (0.14, 0.26) | 0.26 (0.19, 0.32) | 0.24 (0.179, 0.30) | 0.26 (0.20, 0.32) | 0.28 (0.214, 0.34) |
|  | **ALM disconnected** | 0.20 (0.13, 0.28) | 0.20 (0.13, 0.26) | 0.29 (0.21, 0.37) | 0.14 (0.084, 0.21) | 0.25 (0.17, 0.33) | 0.15 (0.095, 0.22) |
|  | **RP single** | 0.98 (0.96, 1.00) | 0.97 (0.95, 1.00) | 0.95 (0.92, 0.98) | 0.95 (0.920, 0.98) | 0.97 (0.95, 0.99) | 0.96 (0.933, 0.99) |
|  | **RP disconnected** | 1.00 (1.00, 1.00) | 1.00 (1.00, 1.00) | 1.00 (1.00, 1.00) | 1.00 (1.000, 1.00) | 1.00 (1.00, 1.00) | 1.00 (1.000, 1.00) |

* The $\beta$ represents impact of covariates included in aggregate level matching (ALM) and reference prediction (RP) regression. The $\gamma$ represents impact of covariates not included in ALM or RP regression. Scenarios are analysis on RCTs only, using single-arm studies (single), and disconnected RCTs (disconnected).
